# Supplementary material for: Structural and social determinants of health: The multi-ethnic study of atherosclerosis
Source: PLoS One. 2024 Nov 18;19(11):e0313625. doi: 10.1371/journal.pone.0313625 (PMC11573213; doi:10.1371/journal.pone.0313625)
Supplement: S1 Table — (DOCX) [file pone.0313625.s001.docx]

**S1 Table. Macrosocial/structural measures collected by MESA exam**

| **Questionnaire/item** | **1** | **2** | **3** | **4** | **5** | **6** | **7** |
| --- | --- | --- | --- | --- | --- | --- | --- |
| Language spoken (Screening form or Personal History Questionnaire) | X |  |  |  |  |  | X |
| Sex/gender (Household Enumeration form) | X |  |  |  |  |  |  |
| Racialized group (Screening form) | X |  |  |  |  |  |  |
| Hispanic ethnicity (Screening form) | X |  |  |  |  |  |  |
| Place of birth (participant, parent, and grandparent) and years living in US (Personal History form) | X |  |  |  |  |  |  |
| Neighborhood racial/ethnic segregation (GIS derived from Census and ACS) | X | X | X | X | X | * | * |
| * Planned as part of MESA Neighborhoods III Study, but not yet calculated; Exam calendar years: 1, 2000-2002; 2, 2002-2004; 3, 2004-2005; 4, 2005-2007; 5, 2010-2011; 6, 2016-2018; 7, 2022-2024. | | | | | | | |
