# Supplementary material for: Structural and social determinants of health: The multi-ethnic study of atherosclerosis
Source: PLoS One. 2024 Nov 18;19(11):e0313625. doi: 10.1371/journal.pone.0313625 (PMC11573213; doi:10.1371/journal.pone.0313625)
Supplement: S2 Table — (DOCX) [file pone.0313625.s002.docx]

**S2 Table. Papers with a focus on macrosocial/structural variables**

| **Macrosocial/ structural subcategories** | **Total paper**  **(col %)** | **Number of papers where SSDOH is:** | | |
| --- | --- | --- | --- | --- |
|  |  | **Exposure (row %)** | **Outcome**  **(row %)** | **Stratification/ effect modification variable**  **(row %)** |
| Racialized/ethnic group | 215 (88%) | 74 (34%) | 0 (0%) | 147 (68%) |
| Sex/gender | 74 (30%) | 21 (28%) | 0 (0%) | 53 (72%) |
| Segregation | 9 (4%) | 7 (78%) | 0 (0%) | 2 (22%) |
| Acculturation | 22 (9%) | 22 (100%) | 0 (0%) | 3 (14%) |
| Total (row %) | 245 (100%) | 103 (42%) | 0 (0%) | 162 (66%) |
| Note: Rows or columns are not mutually exclusive categories | | | | |
