# Supplementary material for: Structural and social determinants of health: The multi-ethnic study of atherosclerosis
Source: PLoS One. 2024 Nov 18;19(11):e0313625. doi: 10.1371/journal.pone.0313625 (PMC11573213; doi:10.1371/journal.pone.0313625)
Supplement: S3 Table — (DOCX) [file pone.0313625.s003.docx]

**S3 Table. Inequality measures collected by MESA exam**

| **Questionnaire/item** | **1** | **2** | **3** | **4** | **5** | **6** | **7** | **TFU 23** |
| --- | --- | --- | --- | --- | --- | --- | --- | --- |
| Employment status and type (Health & Life questionnaire and/or Personal History Questionnaire) | X | X | X | X | X | X | X |  |
| Household income and number of family members supported by income (Personal History Questionnaire) | X | X | X |  | X | X | X |  |
| Car ownership (Personal History form or Health & Life questionnaire) |  | X | X |  | X |  | X |  |
| Investments (Personal History form or Health & Life questionnaire) |  | X | X |  | X |  | X |  |
| Land/property ownership (Personal History form or Health & Life questionnaire) |  | X | X |  | X |  | X |  |
| Mother’s education (Personal History form) |  | X |  |  |  |  |  |  |
| Father’s education (Personal History form) |  | X |  |  |  |  |  |  |
| Health insurance (Personal History form) | X | X | X | X | X | X | X |  |
| Own or rent residence (Personal History form or Health & Life questionnaire) | X | X | X |  | X |  | X |  |
| Educational attainment (Personal History form) | X |  |  |  |  |  | X |  |
| Early-life educational quality (Telephone follow-up form) |  |  |  |  |  |  |  | X |
| TFU = Telephone follow-up 23 from August 2021 through August 2022. Exam calendar years: 1, 2000-2002; 2, 2002-2004; 3, 2004-2005; 4, 2005-2007; 5, 2010-2011; 6, 2016-2018; 7, 2022-2024. | | | | | | | | |
