# Supplementary material for: Structural and social determinants of health: The multi-ethnic study of atherosclerosis
Source: PLoS One. 2024 Nov 18;19(11):e0313625. doi: 10.1371/journal.pone.0313625 (PMC11573213; doi:10.1371/journal.pone.0313625)
Supplement: S4 Table — (DOCX) [file pone.0313625.s004.docx]

**S4 Table. Papers with a focus on inequalities**

| **Inequalities subcategories** | **Total papers**  **(col %)** | **Number of papers where SSDOH is:** | | |
| --- | --- | --- | --- | --- |
|  |  | **Exposure (col %)** | **Outcome (col %)** | **Stratification/ effect modification variable**  **(col %)** |
| Individual socioeconomic status (e.g., education, income, wealth, SES index) | 59 (87%) | 40 (68%) | 0 (0%) | 20 (34%) |
| Health insurance coverage | 4 (6%) | 4 (100%) | 0 (0%) | 0 (0%) |
| Employment status/ occupational role | 17 (26%) | 14 (78%) | 0 (0%) | 4 (22%) |
| Total (row %) | 68 (100%) | 50 (74%) | 0 (0%) | 20 (29%) |
| Note: Rows or columns are not mutually exclusive categories | | | | |
