# Supplementary material for: Structural and social determinants of health: The multi-ethnic study of atherosclerosis
Source: PLoS One. 2024 Nov 18;19(11):e0313625. doi: 10.1371/journal.pone.0313625 (PMC11573213; doi:10.1371/journal.pone.0313625)
Supplement: S5 Table — (DOCX) [file pone.0313625.s005.docx]

**S5 Table. Built environment measures collected by MESA exam**

| **Questionnaire/item** | **1** | **2** | **3** | **4** | **5** | **6** | **7** | **TFU 9** | **TFU 10** | **TFU 13** |
| --- | --- | --- | --- | --- | --- | --- | --- | --- | --- | --- |
| Distance from home to regular food shopping location (Neighborhood Activities Questionnaire) |  | X | |  |  |  |  |  |  |  |
| Distance from home to regular exercise location (Neighborhood Activities Questionnaire) |  | X | |  |  |  |  |  |  |  |
| Neighborhood walking environment (Health & Life questionnaire, Neighborhood Activities Questionnaire, or Neighborhood Questionnaire) |  | X | |  | X |  | X |  |  |  |
| Facilities/destinations within walking distance of home (e.g., park, pool/beach, bicycle path) (Neighborhood Activities Questionnaire or Neighborhood Questionnaire) |  | X | |  |  |  | X |  |  |  |
| Sidewalks/pedestrian amenities in neighborhood (Neighborhood Activities or Neighborhood Questionnaire) |  | X | |  |  |  | X |  |  |  |
| Neighborhood aesthetics (e.g., attractive) (Neighborhood Activities Questionnaire or Neighborhood Questionnaire) |  | X | |  | X |  | X |  |  |  |
| Food access (places to buy fruits/vegetables, fast food) (Neighborhood Activities Questionnaire or Neighborhood questionnaire) |  | X | |  | X |  | X |  |  |  |
| Residence building type (e.g., single family, manufactured home) and age of home (MESA Air Questionnaire, Home Information Questionnaire, Environmental Exposures Questionnaire or Telephone follow-up form) |  |  | X | | X |  | X | X | X | X |
| Indoor facilities related to air pollution exposure (exhaust fan, air conditioning, stove/range type) (MESA Air Questionnaire, Home Information Questionnaire or Environmental Exposures Questionnaire) |  |  | X | | X |  | X |  |  |  |
| Home windows facing alley/streets (Home Information Questionnaire) |  |  |  |  | X |  |  |  |  |  |
| Neighborhood aging environment / places for older adults (e.g., libraries, community centers)) (Neighborhood Questionnaire) |  |  |  |  |  |  | X |  |  |  |
| Neighborhood places to learn (e.g., library lectures) (Neighborhood Questionnaire) |  |  |  |  |  |  | X |  |  |  |
| Neighborhood has public art (Neighborhood Questionnaire) |  |  |  |  |  |  | X |  |  |  |
| Neighborhood has public spaces/places to gather (Neighborhood Questionnaire) |  |  |  |  |  |  | X |  |  |  |
| Residence type (single family home, assisted living, nursing home) (Personal History Questionnaire) |  |  |  |  |  |  | X |  |  |  |
| Neighborhood greenery (Neighborhood Questionnaire) |  |  |  |  |  |  | X |  |  |  |
| Neighborhood park space | X | X | X | X | X | * | * |  |  |  |
| Neighborhood greenness (NDVI) and NLCD greenspace (forest, open space) (GIS derived) | X | X | X | X | X | X |  |  |  |  |
| Neighborhood recreational facilities (GIS derived) | X | X | X | X | X | * | * |  |  |  |
| Neighborhood street connectivity (e.g. intersection density, network ratio) (GIS derived) | X | X | X | X | X | * | * |  |  |  |
| Neighborhood land uses (retail, residential, commercial) (GIS derived) | X | X | X | X | X | * | * |  |  |  |
| Neighborhood destinations (e.g. walking, for cognition) derived from business data (GIS derived) | X | X | X | X | X | * | * |  |  |  |
| WalkScore™ and TransitScore™ (GIS derived) |  |  | X |  | X |  |  |  |  |  |
| Neighborhood food environment (e.g. healthy food stores, supermarkets, fast food) derived from business data (GIS derived) | X | X | X | X | X | * | * |  |  |  |
| Neighborhood tobacco and alcohol environment (e.g. retailers) derived from business data (GIS derived) | X | X | X | X | X | * | * |  |  |  |
| Neighborhood medical facilities (e.g. hospitals, acute care, pharmacies, ambulatory care) derived from business data (GIS derived) | * | * | * | * | * | * | * |  |  |  |
| Neighborhood transportation (e.g. distance to bus stops, distance to train lines) derived from local data (GIS derived) | X | X | X | X | X | * | * |  |  |  |
| Neighborhood destinations for social engagement derived from business data (GIS derived) | X | X | X | X | X | * | * |  |  |  |
| Changes to neighborhood built environment over time (e.g., gentrification; more expensive grocery stores, new buildings on vacant lots) (PACER questionnaire within Neighborhood Questionnaire) |  |  |  |  |  |  | X |  |  |  |
| Neighborhood environment measures reported through questionnaire to community informants (“Community Survey”) |  | `` | X | X | X |  |  |  |  |  |
| TFU = Telephone Follow-up, where TFU 9: August 2008 – October 2010, TFU 10: May 2009 – September 2011, TFU 13: August 2012 – January 2014; GIS = Geographic Information System / objective measures  * Planned as part of MESA Neighborhoods III Study, but not yet calculated  NOTES: (1) The Neighborhood Activities Questionnaire includes the ancillary MESA Neighborhoods Study questions which were asked over the span of Exam 2 and 3 (i.e., asked at one time point during that span), (2) This table provides an overall summary of the major types of built environment data available by Exam; variables outside of these subcategories may also be available. Researchers wishing to use MESA data need to consult with the forms and exam-specific data dictionaries to determine the specific variables available by Exam. Exam calendar years: 1, 2000-2002; 2, 2002-2004; 3, 2004-2005; 4, 2005-2007; 5, 2010-2011; 6, 2016-2018; 7, 2022-2024. | | | | | | | | | | |
