# Supplementary material for: Structural and social determinants of health: The multi-ethnic study of atherosclerosis
Source: PLoS One. 2024 Nov 18;19(11):e0313625. doi: 10.1371/journal.pone.0313625 (PMC11573213; doi:10.1371/journal.pone.0313625)
Supplement: S6 Table — (DOCX) [file pone.0313625.s006.docx]

**S6 Table. Papers with a focus on built environment variables**

| **Built environment subcategories** | **Total papers**  **(col %)** | **Number of papers where SSDOH is:** | | |
| --- | --- | --- | --- | --- |
|  |  | **Exposure**  **(row %)** | **Outcome**  **(row %)** | **Stratification/ effect modification variable**  **(row %)** |
| PA/recreational environment (GIS or survey) | 31 (52%) | 25 (81%) | 3 (10%) | 4 (13%) |
| Food environment (GIS or survey) | 34 (57%) | 28 (82%) | 3 (9%) | 3 (9%) |
| Aesthetics (survey) | 4 (7%) | 3 (75%) | 0 (0%) | 2 (50%) |
| Population density/urbanicity (GIS) | 9 (15%) | 7 (78%) | 1 (11%) | 2 (22%) |
| Street connectivity (GIS) | 10 (17%) | 9 (90%) | 0 (0%) | 2 (20%) |
| Walking destinations/walkability (GIS) | 22 (37%) | 21 (95%) | 1 (5%) | 1 (5%) |
| Transit access (GIS) | 4 (7%) | 4 (100%) | 0 (0%) | 0 (0%) |
| Parks/greenspace (GIS) | 6 (10%) | 6 (100%) | 0 (0%) | 1 (17%) |
| Total (row %) | 60 (100%) | 53 (88%) | 3 (5%) | 6 (10%) |
| PA= physical activity  Note: Rows or columns are not mutually exclusive categories | | | | |
