# Supplementary material for: Structural and social determinants of health: The multi-ethnic study of atherosclerosis
Source: PLoS One. 2024 Nov 18;19(11):e0313625. doi: 10.1371/journal.pone.0313625 (PMC11573213; doi:10.1371/journal.pone.0313625)
Supplement: S7 Table — (DOCX) [file pone.0313625.s007.docx]

**S7 Table. Social context measures collected by MESA exam**

| **Questionnaire/item** | **1** | **2** | **3** | **4** | **5** | **6** | **7** |
| --- | --- | --- | --- | --- | --- | --- | --- |
| Neighborhood social cohesion (Health & Life Questionnaire or Neighborhood Questionnaire) | X |  |  |  | X |  | X |
| Neighborhood is friendly (Neighborhood Activities Questionnaire) |  | X | |  |  |  |  |
| Neighborhood racial/ethnic composition including ethnic enclaves (GIS derived from Census and ACS) | X | X | X | X | X | * | * |
| Neighborhood socioeconomic status and deprivation (GIS derived from Census and ACS) | X | X | X | X | X | * | * |
| Neighborhood Age composition and segregation (GIS derived) | X | X | X | X | X | * | * |
| Changes to neighborhood social environment over time (e.g., trust people moving into neighborhood, shift in neighborhood demographics) (PACER questionnaire within Neighborhood Questionnaire) |  |  |  |  |  |  | X |
| Neighborhood environment measures reported through questionnaire to community informants (“Community Survey”) |  |  | X | X | X |  |  |
| GIS = Geographic Information System/objective measures; ACS = American Community Survey  * Planned as part of MESA Neighborhoods III Study, but not yet calculated  NOTES: (1) The Neighborhood Activities Questionnaire includes the ancillary MESA Neighborhoods Study questions which were asked over the span of Exam 2 and 3 (i.e., asked at one time point during that span), (2) This table provides an overall summary of the major types of social context data available by Exam; variables outside of these subcategories may also be available. Researchers wishing to use MESA data need to consult with the forms and exam-specific data dictionaries to determine the specific variables available by Exam. Exam calendar years: 1, 2000-2002; 2, 2002-2004; 3, 2004-2005; 4, 2005-2007; 5, 2010-2011; 6, 2016-2018; 7, 2022-2024 | | | | | | | |
