# Supplementary material for: Structural and social determinants of health: The multi-ethnic study of atherosclerosis
Source: PLoS One. 2024 Nov 18;19(11):e0313625. doi: 10.1371/journal.pone.0313625 (PMC11573213; doi:10.1371/journal.pone.0313625)
Supplement: S8 Table — (DOCX) [file pone.0313625.s008.docx]

**S8 Table. Papers with focus on social context variables**

| **Social context subcategories** | **Total papers**  **(col %)** | **Number of papers where SSDOH is:** | | |
| --- | --- | --- | --- | --- |
|  |  | **Exposure**  **(row %)** | **Outcome**  **(row %)** | **Stratification/ effect modification variable**  **(row %)** |
| Neighborhood SES or distance from residence to wealthy area | 27 (60%) | 25 (93%) | 0 (0%) | 2 (7%) |
| Neighborhood social environment composite (social cohesion, safety, and/or aesthetics) | 13 (29%) | 10 (77%) | 0 (0%) | 2 (15%) |
| Neighborhood social cohesion | 16 (36%) | 15 (94%) | 2 (13%) | 1 (6%) |
| Neighborhood composition (by race, ethnicity, immigrants, or age) | 6 (13%) | 6 (100%) | 1 (17%) | 0 (0%) |
| Total (row %) | 45 (100%) | 40 (89%) | 3 (7%) | 5 (11%) |
| Note: Rows or columns are not mutually exclusive categories | | | | |
