# Supplementary material for: Structural and social determinants of health: The multi-ethnic study of atherosclerosis
Source: PLoS One. 2024 Nov 18;19(11):e0313625. doi: 10.1371/journal.pone.0313625 (PMC11573213; doi:10.1371/journal.pone.0313625)
Supplement: S9 Table — (DOCX) [file pone.0313625.s009.docx]

**S9 Table. Stressor measures collected by MESA exam**

| **Questionnaire/item** | **1** | **2** | **3** | **4** | **5** | **6** | **7** | **TFU**  **20** |
| --- | --- | --- | --- | --- | --- | --- | --- | --- |
| Neighborhood safety (Neighborhood Questionnaire, Neighborhood Activities Questionnaire, Health & Life Questionnaire) | X | X | |  | X |  | X |  |
| Days and hours per work working (Physical Activity Questionnaire) | X | X | X |  |  | X | X |  |
| Job demands, control, security (Health & Life Questionnaire) |  | X |  |  |  |  |  |  |
| Obtain medical care at emergency room (Personal History Questionnaire) | X | X | X | X | X | X | X |  |
| Environmental tobacco smoke in home (Residential History Supplement Questionnaire and/or MESA Air Questionnaire) |  |  | X | X |  |  |  |  |
| Environmental tobacco smoke at work (Residential History Supplement Questionnaire) |  |  | X |  |  |  |  |  |
| Environmental tobacco smoke exposure as child or adult (Personal History Questionnaire) | X |  |  | X | X | X | X |  |
| Chronic Burden Scale (health problem, job problem, financial strain, relationship problem) (Health & Life Questionnaire) | X |  | X | X |  |  |  |  |
| Traffic exposure (MESA Air Questionnaire) |  |  | X | |  |  |  |  |
| Neighborhood crime (GIS derived) | X | X | X | X | X |  |  |  |
| Neighborhood violence/crime (Neighborhood Activities Questionnaire or Neighborhood Questionnaire or Health & Life Questionnaire) | X | X | |  | X |  | X |  |
| Neighborhood traffic (Neighborhood questionnaire or Neighborhood Activities Questionnaire) | X | X | |  |  |  | X |  |
| Neighborhood psychosocial disorder (e.g., trash, litter, noise) (Neighborhood Activities Questionnaire, Health & Life Questionnaire or Neighborhood Questionnaire) |  | X | |  | X |  | X |  |
| Time spent on heavy traffic roads (Home Information Questionnaire or Environmental Exposures Questionnaire) |  |  |  |  | X |  | X |  |
| Use of coal, wood, etc. to heat home (indoor air pollutants) (Personal History Questionnaire) |  |  |  |  |  | X |  |  |
| Lifetime and Everyday Discrimination (Health & Life Questionnaire) | X |  |  |  |  |  | X |  |
| Discrimination experienced in last day (Daily Stress Survey) |  |  |  |  |  |  | X |  |
| Changes to neighborhood environment over time causing tension/stress (e.g., gentrification causing tension between neighbors) (PACER questionnaire within Neighborhood Questionnaire) |  |  |  |  |  |  | X |  |
| Noise inside or outside makes difficult to sleep (Sleep Study Questionnaire) |  |  |  |  |  |  | X |  |
| Air pollutants measured from air pollution modeling / MESA Air monitors (e.g., PM_2.5_, O_3_) | X | X | X | X | X | * | * |  |
| GIS= Geographic Information System/objective measures; PACER= Perceptions About Changes in Environments and Residents questionnaire; TFU = Telephone follow-up 20 from August 2018 – August 2019  * Planned as part of MESA Lung IV Study, but not yet calculated  NOTES: (1) The Neighborhood Activities Questionnaire includes the ancillary MESA Neighborhoods Study questions which were asked over the span of Exam 2 and 3 (i.e., asked at one time point during that span), (2) This table provides an overall summary of the major types of stressor data available by Exam; variables outside of these subcategories may also be available. Researchers wishing to use MESA data need to consult with the forms and exam-specific data dictionaries to determine the specific variables available by Exam. Exam calendar years: 1, 2000-2002; 2, 2002-2004; 3, 2004-2005; 4, 2005-2007; 5, 2010-2011; 6, 2016-2018; 7, 2022-2024. | | | | | | | | |
