# Supplementary material for: Structural and social determinants of health: The multi-ethnic study of atherosclerosis
Source: PLoS One. 2024 Nov 18;19(11):e0313625. doi: 10.1371/journal.pone.0313625 (PMC11573213; doi:10.1371/journal.pone.0313625)
Supplement: S10 Table — (DOCX) [file pone.0313625.s010.docx]

**S10 Table. Papers with focus on stressors**

| **Stressor subcategories** | **Total papers**  **(col %)** | **Number of papers where SSDOH variable is:** | | |
| --- | --- | --- | --- | --- |
|  |  | **Exposure**  **(row %)** | **Outcome (row %)** | **Stratification/ effect modification variable**  **(row %)** |
| Air pollution / Environmental Tobacco Smoke | 68 (47%) | 57 (84%) | 12 (18%) | 1 (1%) |
| Neighborhood problems (e.g., safety, crime, disorder) | 35 (24%) | 30 (89%) | 2 (6%) | 2 (9%) |
| Chronic stress | 27 (19%) | 23 (81%) | 0 (0%) | 5 (26%) |
| Occupational exposures or job demands | 14 (10%) | 14 (100%) | 0 (0%) | 2 (14%) |
| Discrimination | 13 (9%) | 11 (85%) | 0 (0%) | 2 (15%) |
| Total (row %) | 145 (100%) | 129 (89%) | 13 (9%) | 11 (8%) |
| Note: Rows or columns are not mutually exclusive categories | | | | |
