# Supplementary material for: Structural and social determinants of health: The multi-ethnic study of atherosclerosis
Source: PLoS One. 2024 Nov 18;19(11):e0313625. doi: 10.1371/journal.pone.0313625 (PMC11573213; doi:10.1371/journal.pone.0313625)
Supplement: S11 Table — (DOCX) [file pone.0313625.s011.docx]

**S11 Table. Social integration/support measures collected by MESA exam**

| **Questionnaire/item** | **1** | **2** | **3** | **4** | **5** | **6** | **7** |
| --- | --- | --- | --- | --- | --- | --- | --- |
| Days and hours per week in volunteer work (Physical Activity Questionnaire) | X | X | X |  |  | X | X |
| Volunteer work (Personal History Questionnaire) |  |  |  |  | X |  |  |
| Days and hours per week caring for a child/adult (Physical Activity Questionnaire) | X | X | X |  |  | X | X |
| Number in household (Household Enumeration Form and Personal History Questionnaire) | X |  |  |  |  |  |  |
| Frequency attending religious services (Health & Life Questionnaire) |  | X |  |  |  |  |  |
| Participation in neighborhood organizations (e.g., block association, religious groups, sports leagues, hobby club) (Neighborhood Activities Questionnaire or Neighborhood Questionnaire) |  | X | |  |  |  |  |
| Married/with partner (Health & Life Questionnaire or Personal History Questionnaire) | X |  | X | X | X |  | X |
| Social support (e.g., someone to count on, someone to give advice, someone to provide love and affection) (Health & Life Questionnaire) | X |  | X | X | X |  |  |
| Social isolation (Health & Life Questionnaire) |  |  |  | X | X |  |  |
| Living situation (alone, with others) (Personal History Questionnaire) |  |  |  |  |  |  | X |
| Provided or received emotional/unpaid assistance to or from others (Daily Stress Survey) |  |  |  |  |  |  | X |
| NOTES: (1) The Neighborhood Activities Questionnaire includes the ancillary MESA Neighborhoods Study questions which were asked over the span of Exam 2 and 3 (i.e., asked at one time point during that span), (2) This table provides an overall summary of the major types of social integration/support data available by Exam; variables outside of these subcategories may also be available. Researchers wishing to use MESA data need to consult with the forms and exam-specific data dictionaries to determine the specific variables available by Exam. Exam calendar years: 1, 2000-2002; 2, 2002-2004; 3, 2004-2005; 4, 2005-2007; 5, 2010-2011; 6, 2016-2018; 7, 2022-2024. | | | | | | | |
