# Supplementary material for: Structural and social determinants of health: The multi-ethnic study of atherosclerosis
Source: PLoS One. 2024 Nov 18;19(11):e0313625. doi: 10.1371/journal.pone.0313625 (PMC11573213; doi:10.1371/journal.pone.0313625)
Supplement: S12 Table — (DOCX) [file pone.0313625.s012.docx]

**S12 Table. Papers with a focus on social support/social integration**

| **Social support/social integration**  **Subcategories** | **Total papers**  **(col %)** | **Number of papers where SSDOH is:** | | |
| --- | --- | --- | --- | --- |
|  |  | **Exposure**  **(row %)** | **Outcome**  **(row %)** | **Stratification/ effect modification variable**  **(row %)** |
| Social participation | 1 (5%) | 1 (100%) | 0 (0%) | 0 (0%) |
| Social support | 16 (84%) | 13 (81%) | 0 (0%) | 5 (31%) |
| Social isolation/loneliness | 4 (21%) | 4 (100%) | 0 (0%) | 0 (0%) |
| Household characteristics (marital status, household size) | 5 (26%) | 2 (40%) | 0 (0%) | 3 (60%) |
| Volunteering/caring for others | 1 (5%) | 1 (100%) | 0 (0%) | 0 (0%) |
| Total (row %) | 19 (100%) | 16 (84%) | 0 (0%) | 7 (37%) |
| Note: Rows or columns are not mutually exclusive categories | | | | |
