# Supplementary material for: Structural and social determinants of health: The multi-ethnic study of atherosclerosis
Source: PLoS One. 2024 Nov 18;19(11):e0313625. doi: 10.1371/journal.pone.0313625 (PMC11573213; doi:10.1371/journal.pone.0313625)
Supplement: S13 Table — (DOCX) [file pone.0313625.s013.docx]

**S13 Table. Other social determinants of health-related measures collected by MESA exam**

| **Questionnaire/item** | **1** | **2** | **3** | **4** | **5** | **6** | **7** | **TFU 14** |
| --- | --- | --- | --- | --- | --- | --- | --- | --- |
| Years lived in neighborhood (Neighborhood Questionnaire or Neighborhood Activities questionnaire) | X | X | |  |  |  |  |  |
| Age/birth date (Screening form) | X |  |  |  |  |  |  |  |
| Family members’ sex/gender (Family History form) |  | X |  |  |  |  |  |  |
| Residential History back to 1980 (Residential History Questionnaire) |  |  | X |  |  |  |  | X |
| Secondary residence address (MESA Air Questionnaire) |  |  | X | | X |  | X |  |
| Work address/location (MESA Air Questionnaire) |  |  | X | |  |  |  |  |
| Time spent in neighborhood (Neighborhood Activities questionnaire or Neighborhood Questionnaire) |  | X | |  |  |  | X |  |
| TFU = Telephone follow-up 14 from May 2013 – December 2014  NOTES: (1) The Neighborhood Activities Questionnaire includes the ancillary MESA Neighborhoods Study questions which were asked over the span of Exam 2 and 3 (i.e., asked at one time point during that span), (2) Variables outside of the listed subcategories in this table may also be available. Researchers wishing to use MESA data need to consult with the forms and exam-specific data dictionaries to determine the specific variables available by Exam. Exam calendar years: 1, 2000-2002; 2, 2002-2004; 3, 2004-2005; 4, 2005-2007; 5, 2010-2011; 6, 2016-2018; 7, 2022-2024. | | | | | | | | |
