# Supplementary material for: Structural and social determinants of health: The multi-ethnic study of atherosclerosis
Source: PLoS One. 2024 Nov 18;19(11):e0313625. doi: 10.1371/journal.pone.0313625 (PMC11573213; doi:10.1371/journal.pone.0313625)
Supplement: S14 Table — (DOCX) [file pone.0313625.s014.docx]

**S14 Table. Papers that combined MESA with other cohorts**

| **First author, year** | **Study Title** | **Cohort(s) combined with MESA** |
| --- | --- | --- |
| D'Souza 2021 | Long-Term Exposures to Urban Noise and Blood Pressure Levels and Control Among Older Adults | CHAP |
| Patel 2021 | Hypertension guidelines and coronary artery calcification among South Asians: Results from MASALA and MESA | MASALA |
| Fujiyoshi 2014 | Cross-Sectional Comparison of Coronary Artery Calcium Scores Between Caucasian Men in the United States and Japanese Men in Japan The Multi-Ethnic Study of Atherosclerosis and the Shiga Epidemiological Study of Subclinical Atherosclerosis | SESSA |
| Yamagishi 2010 | Cross-cultural comparison of the sleep-disordered breathing prevalence among Americans and Japanese | CIRCS |
| Barfield 2019 | Epigenome-wide association analysis of daytime sleepiness in the Multi-Ethnic Study of Atherosclerosis reveals African-American-specific associations | CHS |
| Frazier-Wood 2013 | Genetic variants associated with VLDL, LDL and HDL particle size differ with race/ethnicity | GOLDN |
| Manichaikul 2014 | Genome-wide study of percent emphysema on computed tomography in the general population. The Multi-Ethnic Study of Atherosclerosis Lung/SNP Health Association Resource Study | SHARe |
| Wagenknecht 2011 | Correlates of coronary artery calcified plaque in blacks and whites with type 2 diabetes | Family Heart Study, Diabetes Heart Study |
| Park 2022 | Social support, psychosocial risks, and cardiovascular health: Using harmonized data from the Jackson Heart Study, Mediators of Atherosclerosis in South Asians Living in America Study, and Multi-Ethnic Study of Atherosclerosis | JHS, MASALA |
| Kanaya 2014 | Comparing coronary artery calcium among U.S. South Asians with four racial/ethnic groups: The MASALA and MESA studies (vol 234, pg 102, 2014) | MASALA |
| Zhao 2021 | Identification and Predictors for Cardiovascular Disease Risk Equivalents among Adults With Diabetes Mellitus | ARIC, JHS, FHS-Offspring |
| Kanaya 2014 | Understanding the high prevalence of diabetes in U.S. south Asians compared with four racial/ethnic groups: the MASALA and MESA studies | MASALA |
| Kalyani 2014 | Sex differences in diabetes and risk of incident coronary artery disease in healthy young and middle-aged adults | NHANES III Mortality Follow-up Study |
| Zhao 2017 | Interaction between Social/Psychosocial Factors and Genetic Variants on Body Mass Index: A Gene-Environment Interaction Analysis in a Longitudinal Setting | HRS |
| Flores Rosario 2021 | Performance of the Pooled Cohort Equations in Hispanic Individuals Across the United States: Insights From the Multi-Ethnic Study of Atherosclerosis and the Dallas Heart Study | Dallas Heart Study |
| Bancks 2021 | Association of Diabetes Subgroups With Race/Ethnicity, Risk Factor Burden and Complications: The MASALA and MESA Studies | MASALA |
| Smith 2017 | Gene-by-Psychosocial Factor Interactions Influence Diastolic Blood Pressure in European and African Ancestry Populations: Meta-Analysis of Four Cohort Studies | ARIC, JHS, HRS |
| Gutierrez 2022 | Association of Estimated GFR Calculated Using Race-Free Equations With Kidney Failure and Mortality by Black vs Non-Black Race | ARIC, CHS, NHANES III, REGARDS, AASK, MDRD, CRIC |
| Schmitz 2022 | The Socioeconomic Gradient in Epigenetic Ageing Clocks: Evidence from the Multi-Ethnic Study of Atherosclerosis and the Health and Retirement Study | HRS |
| Ware 2015 | Comparative genome-wide association studies of a depressive symptom phenotype in a repeated measures setting by race/ethnicity in the Multi-Ethnic Study of Atherosclerosis | HRS |
| Gujral 2017 | Cardiometabolic Abnormalities Among Normal-Weight Persons From Five Racial/Ethnic Groups in the United States A Cross-sectional Analysis of Two Cohort Studies | MASALA |
| Kanaya 2013 | Glycemic associations with endothelial function and biomarkers among 5 ethnic groups: the Multi-Ethnic Study of Atherosclerosis and the Mediators of Atherosclerosis in South Asians Living in America studies | MASALA |
| Yadlowsky 2018 | Clinical Implications of Revised Pooled Cohort Equations for Estimating Atherosclerotic Cardiovascular Disease Risk | ARIC, CHS, CARDIA, FHS offspring, JHS |
| Foster 2017 | Non-GFR Determinants of Low-Molecular-Weight Serum Protein Filtration Markers in the Elderly: AGES-Kidney and MESA-Kidney | AGES-Kidney |
| Garg 2016 | Ectopic Fat Depots and Coronary Artery Calcium in South Asians Compared With Other Race/Ethnic Groups | MASALA |
| Nazzal 2018 | Educational Inequalities in Cardiovascular Risk Factor and Blood Pressure Control in the Elderly Comparison of MESA Cohort and Chilean NHS Survey Outcome Measures | Chilean NHS Survey |
| Shah 2016 | Less favorable body composition and adipokines in South Asians compared with other US ethnic groups: results from the MASALA and MESA studies | MASALA |
| Hu 2022 | Differences in Metabolomic Profiles Between Black and White Women and Risk of Coronary Heart Disease: an Observational Study of Women From Four US Cohorts | WHI-OS/WHI-HT, JHS, NHS |
| Dulin 2022 | Examining relationships between perceived neighborhood social cohesion and ideal cardiovascular health and whether psychosocial stressors modify observed relationships among JHS, MESA, and MASALA participants | JHS, MASALA |
| Park 2021 | Examining Optimism, Psychosocial Risks, and Cardiovascular Health Using Life's Simple 7 Metrics in the Multi-Ethnic Study of Atherosclerosis and the Jackson Heart Study | JHS |
| Sachs 2020 | Expanded Demographic Norms for Version 3 of the Alzheimer Disease Centers' Neuropsychological Test Battery in the Uniform Data Set | UDS |
| Feinstein 2012 | Racial differences in risks for first cardiovascular events and noncardiovascular death: the Atherosclerosis Risk in Communities study, the Cardiovascular Health Study, and the Multi-Ethnic Study of Atherosclerosis | ARIC, CHS |
| Pandey 2018 | Sex and Race Differences in Lifetime Risk of Heart Failure With Preserved Ejection Fraction and Heart Failure With Reduced Ejection Fraction | CHS |
| Kanaya 2010 | Prevalence and correlates of diabetes in South asian indians in the United States: findings from the metabolic syndrome and atherosclerosis in South asians living in america study and the multi-ethnic study of atherosclerosis |  |
| Levine 2022 | Blood Pressure and Later-Life Cognition in Hispanic and White Adults (BP-COG): A Pooled Cohort Analysis of ARIC, CARDIA, CHS, FOS, MESA, and NOMAS | ARIC, CHS, CARDIA, FOS, NOMAS |
| Breeze 2021 | Epigenome-wide association study of kidney function identifies trans-ethnic and ethnic-specific loci | WHI, JHS, HyperGEN, Generation Scotland, CATHGEN |
| Rodriguez 2020 | Differences in Diet Quality Among Multiple US Racial/Ethnic Groups From the Mediators of Atherosclerosis in South Asians Living in America (MASALA) Study and the Multi-Ethnic Study of Atherosclerosis (MESA | MASALA |
| Mehta 2020 | Predictive Value of Coronary Artery Calcium Score Categories for Coronary Events Versus Strokes: Impact of Sex and Race: MESA and DHS | DHS |
| Gijsberts 2015 | Race/Ethnic Differences in the Associations of the Framingham Risk Factors with Carotid IMT and Cardiovascular Events | ARIC, CAPS, CHS, CIRCS, EAS, FATE, Hoorn, KHID, Malmo, NBS, NOMAS, OSACA2, Tromso, Whitehall |
| Segar 2021 | Development and Validation of Machine Learning-Based Race-Specific Models to Predict 10-Year Risk of Heart Failure A Multicohort Analysis | ARIC, DHS, JHS |
| Hisamatsu 2019 | Coronary Artery Calcium Progression Among the US and Japanese Men | SESSA |
| Nonterah 2022 | Racial and Ethnic Differences in the Association Between Classical Cardiovascular Risk Factors and Common Carotid Intima-Media Thickness: An Individual Participant Data Meta-Analysis | H3Africa AWI‐Gen study, USE‐IMT, FATE, ARIC, NOMA, KIHD, Malmö, Tromsø, NBS |
| Kanaya 2019 | Incidence and Progression of Coronary Artery Calcium in South Asians Compared With 4 Race/Ethnic Groups | MASALA |
| Cameron 2021 | Quantifying the Sex-Race/Ethnicity-Specific Burden of Obesity on Incident Diabetes Mellitus in the United States, 2001 to 2016: MESA and NHANES | NHANES |
| AlRifai 2021 | Distribution of calcium volume, density, number, and type of coronary vessel with calcified plaque in South Asians in the US and other race/ethnic groups: The MASALA and MESA studies | MASALA |
| AlRifai 2018 | Discordance between 10-year cardiovascular risk estimates using the ACC/AHA 2013 estimator and coronary artery calcium in individuals from 5 racial/ethnic groups: Comparing MASALA and MESA | MASALA |
| DeFilippis 2017 | Risk score overestimation: the impact of individual cardiovascular risk factors and preventive therapies on the performance of the American Heart Association-American College of Cardiology-Atherosclerotic Cardiovascular Disease risk score in a modern multi-ethnic cohort | NHANES |
| Abbreviations:  AASK: African American Study of Kidney Disease and Hypertension  ARIC: Atherosclerosis Risk in Communities  AWI-Gen: African-Wits-INDEPTH(International Network for the Demographic Evaluation of Populations and Their Health in Low- and Middle-Income Countries) Genomic Studies  CAPS: Carotid Atherosclerosis Progressions Study  CARDIA: Coronary Artery Development in Young Adults Study  CHAP: Chicago Health and Aging Project  CHS: Cardiovascular Heart Study  CIRCS: Circulatory Risk in Communities Study  CNHS: Chilean National Health Survey  CRIC: Chronic Renal Insufficiency Cohort  DHS: Dallas Heart Study  DHS: Diabetes Heart Study  EAS: Edinburgh Artery Study  FATE: The Firefighters and Their Endothelium Study  FHS: Family Heart Study  FHS-Offspring: Framingham Heart Study Offspring Cohort  GeneSTAR: Genetic Study of Atherosclerosis  GOLDN: Genetics of Lipid Lowering Drugs and Diet Network  H3Africa: Human Hereditary and Health Africa  Hoorn: The Hoorn Study  HRS: Health and Retirement Study  JHS: Jackson Heart Study  KHID: Kuopio Ischaemic Heart Disease Risk Factor Study  Malmo: Malmo Diet and Cancer Study  MASALA: Mediators of Atherosclerosis in South Asians Living in America  MDRD: Modification of Diet in Renal Disease  NBS: Nihmegen Biomedical Study 2  NHANES III: National Health and Nutrition Examination Survey III Mortality Follow-up Study  NHANES: The National Health and Nutrition Examination Survey  NHS: Nurses’ Health Study  NOMAS: Northern Manhattan Study  OSACA2: Osaka Follow-Up Study of Atherosclerosis  REGARDS: Reasons for Geographic and Racial Differences in Stroke  SESSA: Shia Epidemiological Study of Subclinical Atherosclerosis  SHARe: SNP Health Association Resource  Tromsø: Tromsø Study  UDS: Uniform Data Set  USE-IMT: Use Intima-Media Thickness  WHI-OS/WHI-HT: Women’s Health Initiative – Observational Study/Hormone Therapy  Whitehall: Whitehall II Study | | |
