# Supplementary material for: Structural and social determinants of health: The multi-ethnic study of atherosclerosis
Source: PLoS One. 2024 Nov 18;19(11):e0313625. doi: 10.1371/journal.pone.0313625 (PMC11573213; doi:10.1371/journal.pone.0313625)
Supplement: Attachment — Submitted filename: MESA_SDOH_Paper_July26_noendnote.docx [file pone.0313625.s016.docx]

**Structural and Social Determinants of Health: The Multi-Ethnic Study of Atherosclerosis**

Lilah M. Besser^a^, Sarah N. Forrester^b^, Milla Arabadjian^c^, Michael P. Bancks^d^, Margaret Culkin^e^, Kathleen M. Hayden^e^, Elaine T. Le^a^, Isabelle Pierre-Louis^b^, Jana A. Hirsch^f^

^a^ Department of Neurology, Comprehensive Center for Brain Health, University of Miami, Boca Raton, Florida, United States of America

^b^ Division of Epidemiology, Department of Population and Quantitative Health Sciences, University of Massachusetts Chan Medical School, Worcester, Massachusetts, United States of America

^c^ Department of Foundations of Medicine, NYU Grossman Long Island School of Medicine, Mineola, New York, United States of America

^d^ Department of Epidemiology and Prevention, Wake Forest University School of Medicine, Winston-Salem, North Carolina, United States of America

^e^Department of Social Sciences and Health Policy, Wake Forest University School of Medicine, Winston-Salem, North Carolina, United States of America

^f^ Urban Health Collaborative and Department of Epidemiology and Biostatistics, Dornsife School of Public Health, Drexel University, Philadelphia, Pennsylvania, United States of America

**Corresponding author:**

Email: [lmb9767@miami.edu](mailto:lmb9767@miami.edu) (LB)

**Abstract**

**Background**: Researchers have increasingly recognized the importance of structural and social determinants of health (SSDOH) as key drivers of a multitude of diseases and health outcomes. The Multi-Ethnic Study of Atherosclerosis (MESA) is an ongoing, longitudinal cohort study of subclinical cardiovascular disease (CVD) that has followed geographically and racially/ethnically diverse participants starting in 2000. Since its inception, MESA has incorporated numerous SSDOH assessments and instruments to study in relation to CVD and aging outcomes. In this paper, we describe the SSDOH data available in MESA, systematically review published papers using MESA that were focused on SSDOH and provide a roadmap for future SSDOH-related studies.

**Methods and Findings**: The study team reviewed all published papers using MESA data (n=2,125) through January 23, 2023. Two individuals systematically reviewed titles, abstracts, and full text to determine the final number of papers (n=431) that focused on at least one SSDOH variable as an exposure, outcome, or stratifying/effect modifier variable of main interest (discrepancies resolved by a third individual). Fifty-seven percent of the papers focused on racialized/ethnic groups or other macrosocial/structural factors (e.g., segregation), 16% focused on individual-level inequalities (e.g. income), 14% focused on the built environment (e.g., walking destinations), 10% focused on social context (e.g., neighborhood socioeconomic status), 34% focused on stressors (e.g., discrimination, air pollution), and 4% focused on social support/integration (e.g., social participation). Forty-seven (11%) of the papers combined MESA with other cohorts for cross-cohort comparisons and replication/validation (e.g., validating algorithms).

**Conclusions**: Overall, MESA has made significant contributions to the field and the published literature, with 20% of its published papers focused on SSDOH. Future SSDOH studies using MESA would benefit by using recently added instruments/data (e.g., early life educational quality), linking SSDOH to biomarkers to determine underlying causal mechanisms linking SSDOH to CVD and aging outcomes, and by focusing on intersectionality, understudied SSDOH (i.e., social support, social context), and understudied outcomes in relation to SSDOH (i.e., sleep, respiratory health, cognition/dementia).

**INTRODUCTION**

Social determinants of health (SDOH) are the “conditions in the environments where people are born, live, learn, work, play, worship, and age that affect a wide range of health, functioning, and quality-of-life outcomes and risks” [1]. SDOH have been categorized and conceptualized using a wide range of conceptual frameworks, but in common, these frameworks outline how SDOH have multiple spheres of influence (e.g., individual, interpersonal, community, and societal levels) and encompass multiple domains (e.g., sociocultural environment and neighborhood physical/built environment) [2]. Numerous health conditions and behaviors have been associated with SDOH, including but not limited to physical activity, diet, cardiovascular and cerebrovascular disease, cognitive function and dementia risk, and mortality (e.g., [3-6]). Differences in numerous health outcomes have been observed by minoritized racial and ethnic groups, which are socially constructed identities that have experienced longstanding systemic racism that continues to impose on these groups lower access to socioeconomic and health-promoting opportunities and resources [7, 8]. Residents in zip codes with greater racial/ethnic segregation and lower socioeconomic status have shorter life expectancies. For instance, cities such as Chicago and Washington, D.C. experience significant gaps in life expectancy (i.e., 28 to 30 years difference) depending on the zip code of residence [9].

SDOH are influenced by the overarching structural determinants of health, which are the “social and political mechanisms that generate … stratification and social class divisions in society and that define individual socioeconomic position within hierarchies of power, prestige and access to resources” [10]. Considered together, structural and social determinants of health (SSDOH) are major causes of poorer health outcomes and disparities for economically disadvantaged, racialized, and minoritized individuals and communities [11]. Targeted interventions (e.g., policies, programs, new infrastructure) are needed to reduce socioeconomic and racialized/ethnic disparities in a wide range of health outcomes, but these interventions must be evidence-based and informed by scientifically rigorous research that has been conducted and replicated across the populations that would benefit the most from such interventions.

Despite the extant literature, gaps remain in the evidence for associations between key SSDOH (e.g., structural racism, policies, discrimination, neighborhood-level determinants) and cardiovascular disease and aging-related outcomes. Longitudinal cohort studies of middle to older age adults such as the Multi-Ethnic Study of Atherosclerosis (MESA) are well-poised to address these gaps. Since 2000, MESA has been collecting rich data on SSDOH and cardiovascular and aging outcomes and biomarkers. Yet, many published MESA studies have not taken full advantage of these data to conduct more nuanced studies of SSDOH [12]. The aims of this paper are to: 1) describe the SSDOH measures and instruments available in MESA, 2) summarize MESA’s contributions to the SSDOH field via a literature review of published papers as of January 23, 2023, and 3) identify gaps with respect to SSDOH measures and instruments and SSDOH-related publications that could be targeted for future MESA studies. We categorize SSDOH using the Schulz framework of SSDOH and environmental health promotion: 1) macrosocial/structural factors (e.g., residential segregation and ideologies such as racism); 2) individual-level inequalities (e.g., employment, income, and education); 3) built environment (e.g., land use, transportation access, and park space); 4) social context (e.g., neighborhood social cohesion and neighborhood socioeconomic status); 5) stressors (e.g., air pollution, chronic stress, violence and crime); 6) social integration and social support (e.g., social participation and social support) [13]. The overarching goals of the paper are to provide a guide on SSDOH for existing and new researchers using MESA data, and more broadly, to inform the larger research community on measures that could be added and SSDOH studies that could be conducted in other cohorts.

**METHODS**

MESA is an ongoing, population-based, longitudinal cohort study of subclinical cardiovascular disease (CVD) and progression to clinical disease among individuals enrolled at six US locations (Forsyth County, North Carolina; New York, New York; Baltimore, Maryland; Saint Paul, Minnesota; Chicago, Illinois; Los Angeles, California). Individuals were excluded if they had prevalent CVD at baseline. Details about MESA objectives and design are published elsewhere [12]. Briefly, the study was designed to be racially/ethnically diverse (28% Black, 12% Chinese, 22% Hispanic, and 39% White at Exam 1, based on self-report) and has conducted 7 in-person exams (Exam 1, 2000-2002; Exam 2, 2002-2004; Exam 3, 2004-2005; Exam 4, 2005-2007; Exam 5, 2010-2011; Exam 6, 2016-2018; Exam 7, 2022-2024) and 25 telephone follow-ups between the exams from 2000 to 2023. While CVD outcomes are of primary interest for MESA, numerous biomarkers and aging related outcomes (e.g., magnetic resonance imaging, inflammation markers, polysomnography, cognitive testing) have been collected during participant follow-up. The current study, as a literature review, used de-identified data from published papers and thus did not require Institutional Review Board review and did not require informed consent.

Our review began with an initial list of 2,125 papers identified by the MESA Coordinating Center that had been published using MESA data as of January 23, 2023. This list includes all papers (excluding a small portion of consortium papers) submitted to MESA’s Publications and Presentations Committee for review and approval prior to journal submission (a requirement when using MESA data). The list was imported into Covidence systematic review software (Veritas Health Innovation) so that we could perform a systematic review of papers focused on SSDOH. Six papers were eliminated due to duplication, leaving 2,119 abstracts for initial review (Figure 1). Eight individuals reviewed the abstracts and papers moved from abstract screening to data extraction if the paper met any of the following criteria: 1) a SSDOH was a primary exposure variable; 2) a SSDOH was a primary outcome variable; 3) a primary aim (stated in the title, abstract, or introduction section) was to stratify analyses by an SSDOH or examine effect modification by an SSDOH (e.g., testing statistical interactions); or 4) the paper was descriptive or methodological in nature (i.e., not testing a hypothesis) but one or more SSDOH were the main focus. Two reviewers were required for each paper and each step of the abstract and data extraction stages.

**Figure 1**. Flowchart of literature review resulting in 431 studies using Mult-Ethnic Study of Atherosclerosis to study structural/social determinants of health

After abstract screening, 465 papers initially met eligibility criteria. Data were then extracted using a pre-determined template, which collected basic study data (title, author, year), whether the paper was focused only on methodology or only on air pollution modeling methods, whether the study stratified analyses by or examined effect modification by an SSDOH; whether MESA data were combined with other cohorts (e.g., MASALA); and the major categories of SSDOH included based on the major categories of determinants included in the Schulz framework (e.g., macrosocial/structural, individual-level inequalities, etc.) [13]. During the abstract screening and data extraction process, discrepancies between reviewers were resolved via a third-party tiebreaker/consensus. The full-text review was conducted at the same time as the data extraction, such that papers (n=34) were subsequently excluded during extraction if found to not meet our inclusion criteria listed above. Our review in Covidence resulted in a final sample of 431 papers with a SSDOH focus (20% of screened).

We then used the extracted data on the SSDOH categories to develop figures and tables outlining the number, timing, and categories of SSDOH measures used in MESA papers published as of January 23, 2023. Health outcomes were also extracted in 9 categories: (1) biomarkers (e.g., carotid ultrasound, magnetic resonance imaging), (2) cardiovascular disease (CVD) outcomes and risk factors (e.g., myocardial infarction, atrial fibrillation, hypertension), (3) lifestyle behaviors (e.g., diet and physical activity), (4) mental health (depression, anxiety), (5) cognition/dementia (e.g., process speed and global cognition test scores), (6) sleep outcomes (e.g., apnea, daytime sleepiness, actigraphy), (7) other health outcomes (e.g., mortality, obesity, cancer), (8) genetics (e.g., single nucleotide polymorphisms (SNP)), and (9) no health outcomes (e.g., air pollution methodology papers). Reported SSDOH and health outcome categories are not mutually exclusive because some papers include multiple SSDOH and/or outcomes.

**RESULTS**

Table 1 provides the categories of SSDOH data collected at each of the seven completed MESA exams conducted from 2000-2023. SSDOH data were also collected at some telephone follow-up (FU) calls, which are conducted in between the in-person MESA exams and typically ask questions on general health, medical conditions and procedures, medications, admissions, and death. See the Supplemental Tables for more detailed descriptions of the SSDOH data collected, including SSDOH collected at FU calls.

**Table 1. Variables collected by structural and social determinants of health category**

| **SSDOH category** | **Variable/instrument collected (data source)** | **Exam** | | | | | | |
| --- | --- | --- | --- | --- | --- | --- | --- | --- |
|  |  | **1** | **2** | **3** | **4** | **5** | **6** | **7** |
| Macrosocial factors | Racialized / ethnic group (questionnaire) | X |  |  |  |  |  |  |
|  | Sex/gender (questionnaire) | X |  |  |  |  |  |  |
|  | Acculturation (e.g., place of birth) (questionnaire) | X |  |  |  |  |  | X |
|  | Neighborhood racial/ethnic segregation (GIS) | X | X | X` | X | X | * | * |
| Inequalities | Employment and health insurance (questionnaire) | X | X | X | X | X | X | X |
|  | Income (questionnaire) | X | X | X |  | X | X | X |
|  | Investments, car, and property ownership (questionnaire) |  | X | X |  | X |  | X |
|  | Participant’s education (questionnaire) | X |  |  |  |  |  | X |
|  | Mother and father’s education (questionnaire) |  | X |  |  |  |  |  |
|  | Own or rent residence (questionnaire) | X | X | X |  | X |  | X |
| Built environment | Physical activity/recreational environment (GIS or questionnaire) | X | X | X | X | X | * | * |
|  | Food environment (GIS or questionnaire) | X | X | X | X | X | * | * |
|  | Aesthetics (questionnaire) |  | X | |  | X |  | X |
|  | Population density/urbanicity (GIS) | X | X | X | X | X | * | * |
|  | Street connectivity (GIS) | X | X | X | X | X | X | X |
|  | Walking destinations/walkability (GIS) | X | X | X | X | X | * | * |
|  | Transit access (GIS) | X | X | X | X | X | * | * |
|  | Parks/greenspace (GIS) | X | X | X | X | X | X | * |
| Social context | Neighborhood social cohesion (questionnaire) | X | X | |  | X |  | X |
|  | Changes to neighborhood social environment (e.g., demographics) (questionnaire) |  |  |  |  |  |  | X |
|  | Neighborhood socioeconomic status (GIS) | X | X | X | X | X | * | * |
|  | Neighborhood racialized/ethnic group composition (GIS) | X | X | X | X | X | * | * |
|  | Neighborhood age composition/segregation (GIS) | X | X | X | X | X | * | * |
| Stressors | Neighborhood problems (safety, crime, violence, disorder, traffic, noise) (questionnaire) | X | X | |  | X |  | X |
|  | Job demands (questionnaire) | X | X | X |  |  | X | X |
|  | Emergency room medical care (questionnaire) | X | X | X | X | X | X | X |
|  | Environmental tobacco smoke exposure (questionnaire) | X |  | X | X | X | X | X |
|  | Chronic burden/stress (questionnaire) | X |  | X | X |  |  |  |
|  | Indoor or outdoor air pollutant exposure (e.g., use of wood to heat home, time spent on heavy traffic roads) (questionnaire) |  |  |  |  | X | X | X |
|  | Discrimination (questionnaire) | X |  |  |  |  |  | X |
|  | Changes to neighborhood built and social environments suggestive of gentrification (questionnaire) |  |  |  |  |  |  | X |
|  | Neighborhood crime (GIS) | X | X | X | X | X |  |  |
|  | Outdoor air pollutants (e.g., PM_2.5_, O_3_) (GIS) | X | X | X | X | X | * | ***** |
| Social support/ social integration | Social participation (questionnaire) |  | X | X |  |  |  | X |
|  | Household characteristics (e.g., number, marital status) | X |  | X | X | X |  | X |
|  | Volunteer work/caring for others | X | X | X |  | X | X | X |
|  | Social support (e.g., someone to count on) | X |  | X | X | X |  |  |
|  | Social isolation |  |  |  | X | X |  |  |
| * Planned but not yet calculated  GIS = Geographic Information System (i.e., objective measures); SSDOH =Structural and social determinants of health  Note: Major categories reported in this table, with additional categories and more specific measures reported in the supplemental tables. Exam calendar years: 1, 2000-2002; 2, 2002-2004; 3, 2004-2005; 4, 2005-2007; 5, 2010-2011; 6, 2016-2018; 7, 2022-2024. | | | | | | | | |

**Macrosocial/structural measures**

*Data captured in MESA*

Macrosocial/structural measures captured in MESA include sex/gender, racialized/ethnic group, language spoken, and acculturation measures (place of birth, years living in US), which were based on Exam 1 questionnaires (Table 1, Supplemental Table 1). In addition, neighborhood measures of racial and ethnic segregation (i.e., Getis-Ord statistic) were calculated using geographic information systems (GIS) and are/will be available for addresses at Exams 1-7. We use the term “racialized” to indicate that race is socially constructed, and ethnic group refers to self-identified ethnicity (Hispanic or Chinese). Neither of these variables are biological.

*Macrosocial/structural papers*

Two hundred and forty-five papers (57% of total SSDOH sample) have been published using macrosocial/structural measures in MESA (Figure 2) [14-258]. Macrosocial/structural variables were most often (66%) stratification/effect modifier variables (versus exposures or outcomes) and racialized/ethnic group was the most common stratification variable (91%), followed by sex/gender (33%) (Supplemental Table 2). Both racialized/ethnic group and sex/gender were used together as stratifying variables in 23% of papers. Macrosocial/structural variables were exposures in 42% of papers (within those: racialized/ethnic group=72%; acculturation=21%; sex/gender=20%, segregation=7%). Macrosocial/structural factors were examined with biomarkers as the outcome 49% of the time, followed by CVD (26%), other health outcomes (18%), lifestyle behaviors (4%), cognition/dementia (4%), mental health (2%), sleep outcomes (3%), and respiratory outcomes (1%).

**Figure 2.** Papers in each structural and social determinants of health (Abbreviations: SES = socioeconomic status; PA = physical activity; ETS = environmental tobacco smoke)

Papers using MESA data have contributed to the literature on acculturation, most often measured by language spoken at home, place of birth, and years lived in the U.S. These papers measured associations with subclinical CVD [67, 97], CVD risk factors [65, 75, 186], kidney function [59], hypertension [173], depression symptoms [49], cognitive test scores [83], and incident diabetes [188]. Overall, these studies have shown that greater acculturation is associated with worse health outcomes. Studies using data from MESA have also examined the hypothesized healthy immigrant effect by examining health risk factors such as BMI, waist circumference, and health behaviors and found mixed results that are dependent on factors such neighborhood environment, SES, and ethnic subgroup [19, 20, 215].

*Stratification by racialized/ethnic group*

The multi-ethnic nature of the MESA cohort allows for significant contributions to understanding various diseases and health processes both within and between racialized/ethnic groups. One hundred and forty-seven papers stratified by racialized/ethnic group. Of these, 45 (31%) examined racialized/ethnic differences in CVD outcomes, such as coronary heart disease, heart failure, atrial fibrillation, and stroke and CVD risk factors such as diabetes and hypertension. Racialized/ethnic group stratification has been used to examine the differential associations between residential segregation and neighborhood environment on cardiovascular outcomes such as incident CVD [139] and incident hypertension [100], health behaviors such as physical activity, diet quality, and alcohol consumption [41, 42, 68, 99], cardiometabolic risk [163] as well as on cognitive outcomes [33, 259, 260]. For example, Gao *et al*, 2022 [99] found that a composite measure of healthy neighborhood food environment was associated with better diet quality for all participants but that the association was twice as high within participants racialized as Black as it was within participants racialized as White. Three recent studies in MESA have shown that residential segregation is associated with worse health including cognitive decline [34], hypertension [100], and cardiometabolic risk [163]. Using stratification, two reports found that the associations were more pronounced among participants racialized as Black and Hispanic and one found this among only participants racialized as Black. Another study found that living in areas that were historically redlined was associated with worse cardiovascular health, and that the association was more pronounced among participants racialized as Black [175].

Research using MESA has also contributed to literature attempting to disentangle racialized/ethnic group effects and socioeconomic effects, a longstanding issue in health disparities research. One study showed that the expected racialized/ethnic differences were not found but differences by income were pronounced [42]. Another study found inverse socioeconomic patterning of outcomes such as diabetes and hypertension among women racialized as Black and White but mixed relationships among men as well as differing associations depending on the definition of SES (income vs. education) [40].

**Individual-level inequality measures**

*Data captured in MESA*

Inequality measures include numerous measures of individual-level socioeconomic status (e.g., income, education of participant and parents, occupation, home, property, and car ownership) (Table 1, Supplemental Table 3). Health insurance type, number of people supported by household income, and owning/renting a home are also collected. In addition, at telephone follow-up 23, MESA participants are asked a series of questions on early life educational quality (e.g., single room schoolhouse, segregated school) (Supplemental Table 3) [261, 262].

*Inequality papers*

A total of 68 papers (16% of total) focused on individual-level inequalities in three domains: individual socioeconomic status (87%), health care coverage (6%), and employment status/occupational role (26%) (Figure 2, Supplemental Table 4) [5, 23, 35, 39-41, 47, 50, 51, 53, 82, 89, 92, 95, 108, 111, 116, 127, 156, 179, 195, 202, 222, 233, 239, 253, 263-304]. These were modeled as the primary exposure in 74% of the inequalities focused papers and as a stratifying factor in 29% of papers, while no papers included it as an outcome. The three inequality domains were most frequently assessed in relation to biomarkers (43%), lifestyle factors (18%) and other health outcomes (24%) (Table 2). No papers examined associations between inequality measures and sleep outcomes.

**Table 2. Health outcomes examined in MESA studies of structural and social determinants of health**

| **Health outcome** | **Number (%) of studies by SSDOH category** | | | | | | |
| --- | --- | --- | --- | --- | --- | --- | --- |
|  | **Row total (col %)** | **Macrosocial/structural (col %)** | **Inequalities (col %)** | **Built environment (col %)** | **Social context (col %)** | **Stressors (col %)** | **Social support/ integration (col %)** |
| Biomarkers | 204 (47%) | 119 (49%) | 29 (43%) | 3 (5%) | 12 (27%) | 75 (52%) | 8 (42%) |
| CVD outcomes/risk factors | 82 (19%) | 63 (26%) | 6 (9%) | 9 (15%) | 6 (13%) | 16 (11%) | 4 (21%) |
| Lifestyle behaviors | 38 (9%) | 12 (4%) | 12 (18%) | 22 (37%) | 6 (13%) | 8 (6%) | 2 (11%) |
| Respiratory outcomes | 6 (1%) | 3 (1%) | 1 (1%) | 0 (0%) | 0 (0%) | 3 (2%) | 0 (0%) |
| Mental health | 12 (3%) | 6 (2%) | 3 (4%) | 5 (8%) | 7 (16%) | 5 (3%) | 1 (5%) |
| Cognition/dementia | 13 (3%) | 9 (4%) | 4 (6%) | 5 (8%) | 0 (0%) | 1 (1%) | 1 (5%) |
| Sleep outcomes | 13 (3%) | 8 (3%) | 0 (0%) | 3 (5%) | 2 (4%) | 3 (2%) | 0 (0%) |
| Other health outcomes | 71 (16%) | 44 (18%) | 16 (24%) | 14 (23%) | 14 (31%) | 13 (9%) | 4 (21%) |
| Genetics | 9 (2%) | 1 (0%) | 4 (6%) | 0 (0%) | 1 (2%) | 5 (3%) | 2 (11%) |
| No health outcomes* | 31 (7%) | 6 (2%) | 5 (7%) | 5 (8%) | 3 (7%) | 25 (17%) | 0 (0%) |
| Total papers (row %) | 431 (100%) | 245 (57%) | 68 (16%) | 60 (14%) | 45 (10%) | 145 (34%) | 19 (4%) |
| CVD = cardiovascular disease; SSDOH = Structural and social determinants of health  Note: SSDOH and health outcome categories are not mutually exclusive  * e.g., focused on methods | | | | | | | |

Greater individual and parental educational attainment and household income were frequently associated with better clinical, biological, and environmental health measures, positive health behaviors, and lower rates of disease for MESA participants. Consistently, access to health insurance was associated with better health outcomes [82, 195, 278, 284]. Generally, greater wealth was associated with better health outcomes, though a few studies did observe unexpected worse health outcomes with indicators of greater wealth [291, 299]. The transition to retirement was associated with decreases in overall moderate/vigorous physical activity, but increases in leisure walking, all of which may differ by accrued income [127, 288].

**Built environment**

*Data captured in MESA*

As part of the grant-funded MESA Neighborhood ancillary studies, built environment measures in MESA were captured through three primary channels: (1) derived through Geographic Information Systems (GIS); (2) questionnaires to participants; and (3) a series of companion questionnaires to residents (i.e. community informants) living in the same neighborhoods as participants but not part of MESA (known as the “Community Survey”) which were then processed to create Bayesian estimates for MESA neighborhoods (Table 1, Supplemental Table 5). The GIS measures broadly capture land uses (e.g., retail, residential and park space), density (e.g. population density, urbanicity), connectivity for travel (e.g. street connectivity, transit access), and destinations (e.g. walkability, destinations for social engagement, resources for recreation, food stores). GIS data exist across varied neighborhood definitions and scales including census tract and both Euclidean/network buffers of many distances. Data collected via questionnaires (both MESA and Community Survey) include access to places and amenities in the neighborhood (e.g., grocery stores, exercise facilities, parks, places to learn, places for older adults, public art), presence of neighborhood walking supports (e.g. sidewalks and pedestrian amenities such as crosswalks), neighborhood aesthetics, residence (e.g., single family, assisted living) and building type (e.g., manufactured home, condo), and indoor home facilities (e.g., air conditioning, window locations, stove/range type). Several validated scales have been constructed from these questionnaires with the most prominent being walkability, healthy food access, and aesthetics as well as three outside of built environment (i.e. safety, disorder, and social cohesion). Many built environment measures through Exam 5 are available for work addresses in addition to primary residential addresses. Additionally, during telephone follow-up call 14, MESA participants were asked their residential history focused on primary addresses.

*Built environment papers*

Sixty papers (14% of total SSDOH papers) included at least one measure of the built environment (Figure 2) [20, 33, 35, 41, 42, 68, 99, 100, 111, 116, 123, 127, 158, 175, 187, 227, 259, 260, 271, 305-345]. A majority of papers modeled built environment as exposures (88%) with only 5% examining them as outcomes and 10% using them for effect measure modification (Supplemental Table 6). Food environment and physical activity or recreation environment (either GIS or survey-based measures) were the most common built environment metrics, appearing in 57% and 52% of papers, respectively. Walking destinations and walkability (via GIS) were similarly well represented, with 37% of papers examining these measures. While less common, many studies used street connectivity (17%), density/urbanicity (15%), parks/greenspace (10%), aesthetics (7%), and transit access (7%). The built environment domain measures were most frequently assessed in relation to lifestyle factors such as physical activity or nutrition (37%) and cardiovascular disease outcomes or risk (15%) (Table 2). However, MESA built environment work covers a variety of outcomes including mental health (8%), cognition/dementia (8%), sleep (5%), or biomarkers (5%). Although some papers combined built environment with genetic data, no papers examined built environment with genetic outcomes (e.g., DNA methylation). Similarly, no work examined built environment with respiratory outcomes.

The measurable impact of MESA built environment data on the field of neighborhoods and disease have been discussed elsewhere [346]. However, briefly, MESA has found robust evidence of the positive relationship between complementary measures of healthy food environments (individual perceptions, neighborhood informants, store densities) and a healthy diet (e.g., [271, 334, 335, 347, 348]). Similarly, MESA has provided strong evidence that participants who live in supportive neighborhood environments (higher walkability, more recreation destinations, higher density) report higher levels of walking and other physical activity (e.g., [276, 322, 341, 349]). Residents of neighborhoods with worse walking environments had higher odds of severe sleep apnea (CIT), although neighborhood characteristics were not associated with insomnia, sleep efficiency, or sleep fragmentation [314, 316, 350, 351]. Lower social engagement destinations and poorer aesthetic quality have been associated with depressive symptoms and incident depression [330, 333]. Related to lifestyle factors, built environment measures in MESA have been associated with lower hypertension, lower body mass index, and lower insulin resistance and impaired fasting glucose [323, 352, 353]. Evidence on built environments and cognitive function have been mixed: additional greenness has been associated with better cognitive outcomes while increasing density (destination density, street density) were associated with worse overall cognition (e.g., [35, 260, 354, 355]). Similarly, evidence between built environments and cognitive function have been mixed depending on outcome (i.e. global cognitive function, processing speed). Clarifying these associations and competing pathways is the emphasis of the most recent MESA Neighborhood ancillary study [111].

**Social context measures**

*Data captured in MESA*

*Social context* measures collected via questionnaire include neighborhood friendliness, neighborhood social cohesion (e.g., close knit neighborhood, trust in neighbors), and changes to the neighborhood social environment (i.e., Perceptions About Changes in Environments and Residents questionnaire (PACER [356])) (Table 1, Supplemental Table 7). GIS-based measures derived for the MESA Neighborhoods ancillary studies include neighborhood SES, neighborhood racialized/ethnic group, and age group composition. In addition, informants living in the neighborhoods where MESA participants lived completed surveys at Exams 3, 4, and 5 on various aspects of the neighborhood social environment (e.g., neighbors willing to help one another).

*Social context papers*

Forty-five papers (10% of total SSDOH papers) have been published using MESA’s social context measures (Figure 2) [5, 41, 60, 70, 74, 111, 116, 175, 187, 265, 266, 269, 286, 289, 295-298, 302, 303, 316, 327, 330, 332, 333, 336, 344, 351, 357-373]. Of those, 89% investigated social context as the exposure variable (of those: 63% on NSES, 25% on neighborhood social environment, 38% on neighborhood social cohesion, and 15% on neighborhood composition) (Supplemental Table 8). Fewer studies examined social context as an outcome (7%) or as a stratifying variable/effect modifier (11%). Papers investigated associations between social context and biomarkers (27%), CVD outcomes or risk factors (13%), lifestyle factors (13%), mental health (16%), and sleep outcomes (4%), while no studies examined associations with respiratory or cognitive/dementia outcomes.

Recently published papers evidenced the significant contribution of MESA to not only to the field of SSDOH research, but also in going beyond traditional cardiovascular/cerebrovascular health outcomes to link SSDOH to aging biomarkers. For example, these include studies of the socioeconomic gradient in epigenetic aging clocks; social support, psychosocial risks, and cardiovascular health; and discrimination, neighborhood social cohesion, and telomere length [296, 298, 374]. In the first study, in which MESA data were combined with the Health and Retirement Study (HRS), the authors found that individuals living in neighborhoods with higher socioeconomic disadvantage demonstrated accelerated biological aging measured via the GrimAge and DunedinPoAm epigenetic clocks [298]. Social support was not associated with 7 CVH indicators in the second study, which combined data from MESA with the Jackson Heart Study and the Mediators of Atherosclerosis in South Asians Living in America Study (MASALA) [296]. In the third study, neighborhood social cohesion modified the association between discrimination and telomere length attrition over time (i.e., those in low cohesion neighborhoods experiencing more discrimination had faster telomere attrition) [374].

**Stressor measures**

*Data captured in MESA*

Stressor measures collected by questionnaire include neighborhood safety (e.g., violence, crime); neighborhood psychosocial disorder (e.g., trash, noise); job demands, job control, and job security; environmental tobacco smoke at home, work, and during childhood; obtaining medical care at emergency rooms; chronic stress burden (e.g., health problem, job problem, financial strain); traffic exposure; lifetime and everyday discrimination; noise inside and outside of the home that interrupts sleep; and changes to the neighborhood built and social environment (e.g., demographic shifts suggesting gentrification) (Table 1, Supplemental Table 9). During telephone follow-ups 9, 10, and 13, MESA participants answered a questionnaire about residence type (e.g., single family, manufactured mobile home) and in-home and travel behaviors that would affect indoor and outdoor air pollution exposure (e.g., air filter and air conditioning use, locations out of the home spent ≥2 hours/day). Community informants living in MESA participants’ neighborhoods completed surveys at Exams 3, 4, and 5 on stressors such as feeling safe walking in the neighborhood day or night and a lot of graffiti in the neighborhood. In addition, exposure to early life adversity (ELA) was examined at telephone follow-up 20. Items were classified as a measure of ELA under the following domains: parental support/affect; emotional abuse; parental physical affection; physical abuse; household substance abuse; household organization; and parental monitoring.

*Stressor papers*

One hundred and forty-five papers (34% of total SSDOH papers) have been published using MESA’s stressor measures (Figure 2)[5, 25, 30, 39, 50-53, 57, 66, 74, 79, 87, 92-95, 110, 111, 116, 124, 125, 149, 164, 174, 193, 223, 226, 227, 255, 263, 267, 279, 280, 282, 286, 299, 300, 315, 316, 319, 326, 327, 330, 333, 336, 338, 343, 344, 351, 357, 367, 372, 375-466]. Of those, 47% included air pollution and/or environmental traffic measures, 24% included measures of neighborhood stressors (problems, safety, crime, disorder), 19% included measures of chronic stress, 10% included measures of occupational exposures or job demand, and 9% included measures of discrimination (Supplemental Table 10). Most papers (89%) included the stressor variables as the exposure or predictor and far fewer as the outcome (9%) or as a stratification/effect modification variable (8%). Over half (52%) of the papers studied stressor measures in relation to biomarker outcomes, with smaller percentages dedicated to CVD-related outcomes (11%), other health outcomes (9%), lifestyle behaviors (6%), or the other SSDOH categories (i.e., 2% on respiratory outcomes, 3% mental health, 1% cognition/dementia, 2% sleep, 3% genetics, 17% did not include health outcomes (e.g., air pollution methodology)) (Table 2).

The greatest percentage of the papers on stressors (47%) were on air pollution or environmental tobacco smoke (ETS). Twenty-five papers (17% of total focused on stressors) did not study air pollution or other SSDOH as predictors, outcomes, or stratifying/effect modifier variables, but solely reported on air pollution estimate methodology. Importantly, the EPA-funded MESA Air ancillary study led to new spatiotemporal techniques for improved estimates of outdoor air pollutants (e.g., PM_2.5_, O_3_) [429, 467]. These models aimed at capturing pollutant variation in metropolitan areas with a focus on small temporal and spatial scales, and over time the methodology led to the ability to estimate fine-scale exposures across the US, which in turn can be studied in relation to numerous health outcomes.

Recent MESA studies interested in associations with air pollution have shown that short term exposure to air pollution is associated with increased respiratory infections [428] , increased biomarkers for vascular disease [442], increased risk of emphysema [464], and increased likelihood of sleep apnea [390]. One study found that exposure to air pollution accounted for some of the racialized/ethnic group disparities in systolic blood pressure among men [226] while another found no association between air pollution and blood pressure when accounting age and calendar time [376]. Overall, MESA studies mostly show that exposure to short- and long-term air pollution is detrimental to health. Recent MESA papers of ETS exposure found that secondhand smoke exposure is associated with higher risk of atrial fibrillation in non-smokers [433] and that ETS in childhood is associated with more emphysema in adulthood [436]. One study found that working in blue collar workplaces was associated with heavy smoking and with ETS.

Papers that included chronic stress (measured by the chronic burden scale) as a predictor for health-related outcomes reveal mixed findings. Chronic stress was positively associated with incident ASCVD and mortality [382], incident stroke and TIA [404], CVD events [397], endothelial dysfunction [424], central and subcutaneous adiposity [400], and inflammation [450]. Other papers found negative associations between chronic stress and inflammation [223], urinary stress hormones [50], coronary artery calcification [401], and atrial fibrillation [405]. Associations between chronic stress and health-related outcomes may depend on type and length of exposure to stress.

**Social support/social integration**

*Data captured in MESA*

Social support and social integration measures collected via questionnaire include marital status; number in household; living situation (alone, with others); time spent caring for a child/adult; volunteer work; frequency attending religious services; participation in neighborhood organizations (e.g., block association, sport leagues); social support (e.g., someone to give advice, someone to provide love and affection); social isolation; and provided or received emotional/unpaid assistance to or from others (Table 1, Supplemental Table 11).

*Social support papers*

Nineteen papers (4% of total SSDOH papers) have been published using MESA’s social support and social integration measures (Figure 2) [83, 116, 127, 185, 267, 268, 296, 361, 407, 416, 440, 468-475]. Eighty-four percent of publications used these measures as the primary exposure and 37% used social support/social integration measures as stratifying/effect modifier variables (Supplemental Table 12). Primary exposures included social support (84%), social isolation/loneliness (21%), household size or marital status (26%), caregiving (5%), and social participation (5%). Among papers that stratified by social support/integration variables (n=7), 71% stratified by social support measures and 43% of stratified by household characteristics (e.g., marital status). No other measures were used for stratification/effect modification analyses.

Notably, MESA social support and social integration measures were most frequently examined for associations with mechanistic factors, specifically biomarkers (42%), followed by CVD outcomes/risk factors (21%). With respect to biomarkers, Brown *et al*. (2020) found that loneliness was associated with inflammatory biomarker gene expression [268]. Low social support was associated with C-reactive protein (CRP) elevation in men but not in women, though high social support buffered the relationship between stress and CRP among middle-aged women [473]. In addition, a positive association was found between social support and telomere length [473]. Four publications examined social support and social integration measures in the context of CVD risk or CVD outcomes, with only one publication finding a positive association between social support and time delay in CVD disease onset [468].

Eleven percent of publications examined the association of social support with other health outcomes. Lower social support was associated with higher rates of retinopathy, social support was not associated with metabolic syndrome prevalence, and social support was inversely associated with heart rate but not heart rate variability [185, 416, 475]. Another study found a significant association between household size and cognitive test scores, with those living in households with 5 members or more scoring lower on cognitive testing [83]. Lastly, no association was found between social support, change in caregiver status, or marital status, and walking post-retirement [127]. Overall, few MESA studies have examined social support/integration measures in relation to health-related outcomes.

**Additional MESA measures associated with SSDOH**

MESA has also collected data that help provide further details regarding the aforementioned SSDOH measures or that allow for the derivation of new neighborhood GIS measures (Supplemental Table 13). This includes years lived in the neighborhood, age/birth date (can determine birth cohort/cohort effects); family members’ sex/gender including spouse/partner; residential history back to 1980 and secondary residence address history (e.g., snowbirds); and time spent doing activities in the neighborhood. The residential history data allows interested investigators to create new neighborhood measures by linking geocoded addresses (latitudes and longitudes) to environmental data (e.g., satellite imagery, google earth imagery, maps).

**Combination of MESA with other cohorts**

Forty-seven papers (11% of total SSDOH papers) combined MESA with 41 US and non-US based cohorts [5, 31, 44, 61, 80, 85, 88, 90, 102, 107, 112, 130-132, 134, 135, 152, 159, 168, 180, 191, 216, 220, 245, 256, 293, 295, 296, 298, 300, 304, 399, 476-490] (Supplemental Table 14). The most common cohorts combined with MESA include the Mediators of Atherosclerosis in South Asians Living in America Study (MASALA), Atherosclerosis Risk in Communities Study (ARIC), Jackson Heart Study (JHS), and the Health and Retirement Study (HRS) (Supplemental Table 8). For instance, in a recently published paper (2022), MESA data were combined with 14 other cohorts in Africa, Asia, Europe, and North America to investigate whether associations between common cardiovascular risk factors and common carotid intima-media thickness (CIMT) varied by racialized/ethnic group [180]. The study found that high density lipoprotein levels (HDL) were protective only for African American and African individuals. For Asian populations, smoking and glucose displayed the strongest associations with CIMT. Overall, the study noted variations in the strength of associations between the risk factors and CIMT depending on racialized/ethnic group, suggesting that primary prevention strategies need to be tailored to each group. Overall, MESA’s comprehensive data collected on a diverse sample allows for these pooled and comparison studies that have contributed significantly to the literature.

**DISCUSSION**

MESA has contributed over 430 SSDOH papers to the extant literature since its inception. Consistent with MESA’s aim of assembling and following a diverse, multi-ethnic cohort that allows for analyses of specific racialized/ethnic subgroups, approximately half of the studies involved racialized/ethnic group as an exposure or stratifying/effect modifying variable. Moreover, hundreds of published MESA papers focused on a wide range of SSDOH, investigating macrosocial/structural factors such as acculturation and neighborhood racialized/ethnic segregation, individual-level inequalities such as health insurance coverage and retirement status, built environment factors such as greenspace and destinations, social context factors such as neighborhood SES and social cohesion, stressors such as discrimination and air pollution, and social support/integration factors such has loneliness and social participation. MESA’s SSDOH data has been combined with 41 other cohorts across 47 papers for pooled analyses, cross-cohort comparisons, and replication or validation of instruments and algorithms/indices. Over time, additional self-reported SSDOH instruments (e.g., PACER, age-related neighborhood characteristics) questionnaires have been incorporated into MESA exams. These additions are evidence of MESA’s acknowledged importance of understanding how SSDOH impact CVD and aging-related outcomes.

**Gaps in SSDOH related papers to target in future research**

The least studied SSDOH category was social support/integration, suggesting avenues for future research. In particular, only four papers focused on social isolation and loneliness, which was named as a US epidemic and as risk factors for numerous poor health outcomes, including mortality, cardiovascular disease, and cognitive decline in the 2023 US Surgeon General’s report [491]. New research could investigate MESA’s social support/integration measures such as isolation and loneliness as risk factors for CVD and aging outcomes and as mediators or outcomes in relation to other SSDOH risk factors such as retirement status and urbanicity.

Sleep behaviors and patterns can be altered in older age [492]. Older adults can experience sleep apnea, increased daytime sleepiness, and more frequent naps, as well as shorter length of time slept or disrupted sleep at night. While MESA has collected several self-reported (e.g., snoring, nap frequency) and objective measures of sleep (e.g., actigraphy), only 13 of the reviewed studied examined associations between SSDOH and sleep outcomes. Six of these focused on racialized/ethnic group or other demographic comparisons of sleep outcomes, and four examined associations with neighborhood built and social environments. Future MESA studies could center on understudied SSDOH such as noise and social engagement and whether these impact sleep outcomes, in addition to whether the sleep measures mediate or moderate associations between risk factors (e.g., physical activity) and CVD and aging outcomes.

Respiratory outcomes such as COPD and asthma were the least studied health outcomes among MESA’s SSDOH studies (n=6). These papers studied outdoor air pollution, smoking, and SES as predictors of respiratory outcomes, occupational risk for COPD, and lung function prediction equations (i.e., spirometry). None examined indoor air pollution, possible structural (e.g., segregation) or neighborhood determinants of respiratory disease (e.g., neighborhood SES, neighborhood greenspace), which would be fruitful targets for future research.

Up to 40% of dementias including Alzheimer’s disease have been attributed to modifiable risk factors (e.g., physical activity, diet, obesity, diabetes, depression, smoking, and hearing loss) that are heavily influenced by SSDOH [3]. To date, only 13 of MESA’s SSDOH studies centered on cognitive/dementia outcomes. With its numerous SSDOH measures, MESA is well poised to investigate nuanced relationships between SSDOH, modifiable risk factors, and cognitive and dementia outcomes, to better elucidate the contribution of SSDOH to dementia risk.

The importance of intersectionality has been increasingly emphasized in SSDOH and health research. Individuals who have multiple, historically disadvantaged and minoritized identities (e.g., Black women or Hispanic individuals without a high school education) can be at increased risk for numerous diseases and health conditions including CVD and dementia [493, 494]. Yet, no MESA studies as of January 2023 have investigated and framed their studies based on the concept of intersectionality. This is an important next step for SSDOH and health research generally and future MESA studies specifically.

Structural determinants of health include laws, economic and social policies, and governing practices at local, regional, and national scales that determine the distribution of economic and health promoting resources and opportunities. To date, only two MESA studies examined social/economic policies in relation to health outcomes. The first investigated neighborhood cigarette prices and smoking ban policies on smoking behavior, finding that a $1 increase in price was associated with a 20% increased risk of smoking cessation [162]. MESA’s residential address history data provides numerous opportunities for future studies to determine associations between policies at the participants’ neighborhood, county, and state level (e.g., historic redlining) and CVD and aging outcomes. The second paper studied historic redlining (discriminatory mortgage lending practices) and cardiovascular health, finding that Black individuals living in historically redlined areas had worse cardiovascular risk scores based on blood pressure, fasting glucose, cholesterol, body mass index, diet, physical activity, and smoking [175].

Recent SSDOH additions in MESA will become available to researchers in the coming years. This includes self-reported data from the PACER, ELA, and early-life educational quality questionnaires, as well as objectively measured (i.e., GIS-derived) data on google street view (GSV) greenery (i.e., grasses, trees, shrubs) calculated for 2007-on (NIA 5R00AG066949, PI: Pescador), and measures of neighborhood greenness and greenspace (i.e., normalized difference vegetation index and developed open space, forest, and total greenspace derived from the National Land Cover Dataset) that were calculated annually for participants’ addresses from 1980-2019 (NIA R21AG075291, PI: Besser). In addition, the ongoing ancillary MESA Neighborhoods III study (NIA R01AG072634, PI: Hirsch) is developing new survey measures on whether MESA respondents have neighborhood supports for aging as well GIS measures such as age segregation and American Association of Retired Persons (AARP) Livability Index domains (e.g., affordable housing, transportation safety and convenience, clean air and water) [356, 495]. The newly added measures will offer researchers numerous opportunities to collaborate with MESA investigators and investigate novel research hypotheses related to community-level measures of social and built environments.

**Strengths and limitations**

Beyond the contributions and strengths noted above, MESA displays several strengths including its population-based recruitment, the multi-ethnic and geographically diverse sample, and the comprehensive data collected at each exam on self-reported and objectively measured SSDOH as well as on clinical and biomarkers outcomes. While the strengths of MESA’s SSDOH data far outweigh any weaknesses, some common limitations must be noted. The administration timing for some SSDOH instruments (e.g., PACER) limits the analytic sample size because they were added during later exams. As with any longitudinal cohort, MESA has experienced attrition due to death and drop out. For instance, at Exam 7 (2022- 2023), 34% (n=2,317) of the original cohort of 6,814 from Exam 1 (2000-2002) remained. Focusing on SSDOH collected at Exam 7 may result in biased findings if analyses using these data do not account for selection/attrition bias, particularly for differential follow-up by SSDOH factors. Any self-reported SSDOH measures (e.g., questions on psychosocial disorder) may be subject to misclassification or recall bias. Although the sample is geographically and racially/ethnically diverse, any associations found using MESA may not be generalizable to other racialized/ethnic groups or other regions in the US. MESA originally collected data on gender (i.e., male and female), but data are not collected on third/non-binary gender, biological sex assigned at birth, or sexual orientation. Data on secondary/additional racialized/ethnic identities are also not collected (e.g., self-identifying as Black and White). Lastly, the strong correlation between some SSDOH variables (e.g., racial/ethnic segregation and neighborhood SES) should be carefully weighed to determine if statistically controlling for the highly correlated variable or examining effect modification by that variable is feasible.

In this review, we evaluated the major categories of SSDOH collected and investigated in MESA to date, identified the scientific gaps, and outlined numerous possibilities for researchers to pursue in future SSDOH-related studies. New MESA studies can investigate previously unexplored areas by incorporating measures of social support/integration; centering on sleep, respiratory, and cognitive/dementia outcomes; examining structural determinants as well as SSDOH as mediators and moderators; and studying health outcomes with an intersectionality lens. Overall, MESA has substantially added to the extant literature on SSDOH and CVD and aging-related outcomes and provides a valuable resource for researchers interested in pursuing studies on SSDOH and older adults.

**Acknowledgements**

The MESA cohort study was supported by contracts 75N92020D00001, HHSN268201500003I, N01-HC-95159, 75N92020D00005, N01-HC-95160, 75N92020D00002, N01-HC-95161, 75N92020D00003, N01-HC-95162, 75N92020D00006, N01-HC-95163, 75N92020D00004, N01-HC-95164, 75N92020D00007, N01-HC-95165, N01-HC-95166, N01-HC-95167, N01-HC-95168 and N01-HC-95169 from the National Heart, Lung, and Blood Institute, and by grants UL1-TR-000040, UL1-TR-001079, and UL1-TR-001420 from the National Center for Advancing Translational Sciences (NCATS). The authors thank the other investigators, the staff, and the participants of the MESA study for their valuable contributions. A full list of participating MESA investigators and institutions can be found at <http://www.mesa-nhlbi.org>. This paper has been reviewed and approved by the MESA Publications and Presentations Committee.

**References**

1.	Centers for Disease Control and Prevention. About Social Determinants of Health (SDOH) 2021 [01/31/2022]. Available from: https://www.cdc.gov/socialdeterminants/about.html.

2.	Committee on Educating Health Professionals to Address the Social Determinants of Health; Board on Global Health; Institute of Medicine; National Academies of Sciences E, and Medicine. A Framework for Educating Health Professionals to Address the Social Determinants of Health. Washington, DC: National Academies Press (US); 2016.

3.	Adkins-Jackson PB, George KM, Besser LM, Hyun J, Lamar M, Hill-Jarrett TG, et al. The structural and social determinants of Alzheimer's disease related dementias. Alzheimers Dement. 2023;19(7):3171-85. Epub 2023/04/19. doi: 10.1002/alz.13027. PubMed PMID: 37074203.

4.	Hunt JFV, Vogt NM, Jonaitis EM, Buckingham WR, Koscik RL, Zuelsdorff M, et al. Association of Neighborhood Context, Cognitive Decline, and Cortical Change in an Unimpaired Cohort. Neurology. 2021;96(20):e2500-e12. Epub 2021/04/16. doi: 10.1212/WNL.0000000000011918. PubMed PMID: 33853894; PubMed Central PMCID: PMCPMC8205478.

5.	Dulin AJ, Park JW, Scarpaci MM, Dionne LA, Sims M, Needham BL, et al. Examining relationships between perceived neighborhood social cohesion and ideal cardiovascular health and whether psychosocial stressors modify observed relationships among JHS, MESA, and MASALA participants. BMC Public Health. 2022;22(1):1890. Epub 20221011. doi: 10.1186/s12889-022-14270-x. PubMed PMID: 36221065; PubMed Central PMCID: PMCPMC9552445.

6.	Ahmed HM, Blaha MJ, Nasir K, Jones SR, Rivera JJ, Agatston A, et al. Low-risk lifestyle, coronary calcium, cardiovascular events, and mortality: results from MESA. Am J Epidemiol. 2013;178(1):12-21. Epub 20130602. doi: 10.1093/aje/kws453. PubMed PMID: 23733562; PubMed Central PMCID: PMCPMC3698994.

7.	Besser LM, Mitsova D, Williams CL, Wiese L. Redlining and Neighborhood Walking in Older Adults: The 2017 National Household Travel Survey. Am J Prev Med. 2022;63(6):926-34. Epub 2022/08/20. doi: 10.1016/j.amepre.2022.06.010. PubMed PMID: 35985900; PubMed Central PMCID: PMCPMC9691519.

8.	Locke DH, Hall B, Grove JM, Pickett STA, Ogden LA, Aokis C, et al. Residential housing segregation and urban tree canopy in 37 US cities. NPJ Urban Sustain. 2021;1(15).

9.	Ducharme J WE. Your ZIP Code Might Determine How Long You Live—and the Difference Could Be Decades. Time. 2019.

10.	WOrld Health Organization. A conceptual framework for action on the social determinants of health. 2010.

11.	Gomez CA, Kleinman DV, Pronk N, Wrenn Gordon GL, Ochiai E, Blakey C, et al. Addressing Health Equity and Social Determinants of Health Through Healthy People 2030. J Public Health Manag Pract. 2021;27(Suppl 6):S249-S57. Epub 2021/03/18. doi: 10.1097/PHH.0000000000001297. PubMed PMID: 33729197; PubMed Central PMCID: PMCPMC8478299.

12.	Bild DE, Bluemke DA, Burke GL, Detrano R, Diez Roux AV, Folsom AR, et al. Multi-Ethnic Study of Atherosclerosis: objectives and design. Am J Epidemiol. 2002;156(9):871-81. PubMed PMID: 12397006.

13.	Schulz A, Northridge ME. Social determinants of health: implications for environmental health promotion. Health Educ Behav. 2004;31(4):455-71. Epub 2004/08/07. doi: 10.1177/1090198104265598. PubMed PMID: 15296629.

14.	Aboyans V, Criqui MH, McClelland RL, Allison MA, McDermott MM, Goff DC, Jr., et al. Intrinsic contribution of gender and ethnicity to normal ankle-brachial index values: the Multi-Ethnic Study of Atherosclerosis (MESA). J Vasc Surg. 2007;45(2):319-27. doi: 10.1016/j.jvs.2006.10.032. PubMed PMID: 17264011.

15.	Akbar A, Liu K, Michos ED, Brubaker L, Markossian T, Bancks MP, et al. Racial differences in urinary incontinence prevalence and associated bother: the Multi-Ethnic Study of Atherosclerosis. Am J Obstet Gynecol. 2021;224(1):80 e1- e9. doi: 10.1016/j.ajog.2020.07.031. PubMed PMID: 32697955.

16.	Akbar A, Liu K, Michos ED, Brubaker L, Markossian T, Bancks MP, et al. Racial Differences in Urinary Incontinence Prevalence, Overactive Bladder and Associated Bother among Men: The Multi-Ethnic Study of Atherosclerosis. Journal of Urology. 2021;205(2):524-31. doi: 10.1097/Ju.0000000000001353. PubMed PMID: WOS:000606842900051.

17.	Al Rifai M, Cainzos-Achirca M, Kanaya AM, Kandula NR, Dardardi Z, Joshi PH, et al. Discordance between 10-year cardiovascular risk estimates using the ACC/AHA 2013 estimator and coronary artery calcium in individuals from 5 racial/ethnic groups: Comparing MASALA and MESA. Atherosclerosis. 2018;279:122–9.

18.	Al Rifai M, Kanaya AM, Kandula NR, Cainzos-Achirica M, Patel J, Budoff M, et al. Distribution of calcium volume, density, number, and type of coronary vessel with calcified plaque in South Asians in the US and other race/ethnic groups: The MASALA and MESA studies. Atherosclerosis. 2021;317:16–21.

19.	Albrecht SS, Diez Roux AV, Kandula NR, Osypuk TL, Ni H, Shrager S. Immigration assimilation and BMI and waist size: a longitudinal examination among hispanic and chinese participants in the multi-ethnic study of atherosclerosis. Obesity. 2013;21(8):1695-703.

20.	Albrecht SS, Osypuk TL, Kandula NR, Gallo LC, Le-Scherban F, Shrager S, et al. Change in waist circumference with longer time in the United States among Hispanic and Chinese immigrants: the modifying role of the neighborhood built environment. Annals of Epidemiology. 2015;25(10):767-72. doi: 10.1016/j.annepidem.2015.07.003. PubMed PMID: WOS:000361419000008.

21.	Allison MA, Budoff MJ, Nasir K, Wong ND, Detrano R, Kronmal R, et al. Ethnic-specific risks for atherosclerotic calcification of the thoracic and abdominal aorta (from the Multi-Ethnic Study of Atherosclerosis). Am J Cardiol. 2009;104(6):812-7. doi: 10.1016/j.amjcard.2009.05.004. PubMed PMID: 19733716; PubMed Central PMCID: PMCPMC2755558.

22.	Allison MA, Budoff MJ, Wong ND, Blumenthal RS, Schreiner PJ, Criqui MH. Prevalence of and risk factors for subclinical cardiovascular disease in selected US Hispanic ethnic groups: the Multi-Ethnic Study of Atherosclerosis. Am J Epidemiol. 2008;167(8):962-9. Epub 20080217. doi: 10.1093/aje/kwm402. PubMed PMID: 18283034; PubMed Central PMCID: PMCPMC4107279.

23.	Allison MA, Criqui MH, McClelland RL, Scott JM, McDermott MM, Liu K, et al. The effect of novel cardiovascular risk factors on the ethnic-specific odds for peripheral arterial disease in the Multi-Ethnic Study of Atherosclerosis (MESA). J Am Coll Cardiol. 2006;48(6):1190-7. Epub 20060828. doi: 10.1016/j.jacc.2006.05.049. PubMed PMID: 16979004.

24.	Allison MA, Cushman M, Solomon C, Aboyans V, McDermott MM, Goff DC, Jr., et al. Ethnicity and risk factors for change in the ankle-brachial index: the Multi-Ethnic Study of Atherosclerosis. J Vasc Surg. 2009;50(5):1049-56. Epub 20090722. doi: 10.1016/j.jvs.2009.05.061. PubMed PMID: 19628357; PubMed Central PMCID: PMCPMC2783523.

25.	Allison PJ, Jorgensen NW, Fekedulegn D, Landsbergis P, Andrew ME, Foy C, et al. Current work hours and coronary artery calcification (CAC): The Multi-Ethnic Study of Atherosclerosis (MESA). Am J Ind Med. 2020;63(4):348-58. Epub 20191217. doi: 10.1002/ajim.23084. PubMed PMID: 31845385; PubMed Central PMCID: PMCPMC7054127.

26.	Anderson MD, Merkin SS, Everson-Rose SA, Widome R, Seeman T, Magnani JW, et al. Health Literacy Within a Diverse Community-Based Cohort: The Multi-Ethnic Study of Atherosclerosis (vol 41, pg 815, 2020). Journal of Immigrant and Minority Health. 2021;23(4):668-. doi: 10.1007/s10903-020-01137-9. PubMed PMID: WOS:000607486000001.

27.	Austin TR, Nasrallah IM, Erus G, Desiderio LM, Chen LY, Greenland P, et al. Association of Brain Volumes and White Matter Injury With Race, Ethnicity, and Cardiovascular Risk Factors: The Multi-Ethnic Study of Atherosclerosis. J Am Heart Assoc. 2022;11(7):e023159. Epub 20220330. doi: 10.1161/jaha.121.023159. PubMed PMID: 35352569; PubMed Central PMCID: PMCPMC9075451.

28.	Bahrami H, Kronmal R, Bluemke DA, Olson J, Shea S, Liu K, et al. Differences in the incidence of congestive heart failure by ethnicity: the multi-ethnic study of atherosclerosis. Arch Intern Med. 2008;168(19):2138-45. doi: 10.1001/archinte.168.19.2138. PubMed PMID: 18955644; PubMed Central PMCID: PMCPMC3038918.

29.	Bakker JP, Weng J, Wang R, Redline S, Punjabi NM, Patel SR. Associations between Obstructive Sleep Apnea, Sleep Duration, and Abnormal Fasting Glucose The Multi-Ethnic Study of Atherosclerosis. American Journal of Respiratory and Critical Care Medicine. 2015;192(6):745-53. doi: 10.1164/rccm.201502-0366OC. PubMed PMID: WOS:000361420400017.

30.	Bakshis E, Smith JA, Mukherjee B, Lee S, Kardia SL, Diez-Roux AV. Applying Novel Methods for Assessing Individual- and Neighborhood-Level Social and Psychosocial Environment Interactions with Genetic Factors in the Prediction of Depressive Symptoms in the Multi-Ethnic Study of Atherosclerosis. Behav Genet. 2016;46(1):89–99.

31.	Bancks MP, Bertoni AG, Carnethon M, Chen H, Cotch MF, Gujral UP, et al. Association of Diabetes Subgroups With Race/Ethnicity, Risk Factor Burden and Complications: The MASALA and MESA Studies. J Clin Endocrinol Metab. 2021;106(5):e2106-e15. doi: 10.1210/clinem/dgaa962. PubMed PMID: 33502458; PubMed Central PMCID: PMCPMC8063247.

32.	Baron KG, Liu K, Chan C, Shahar E, Hasnain-Wynia R, Zee P. Race and ethnic variation in excessive daytime sleepiness: the multi-ethnic study of atherosclerosis. Behav Sleep Med. 2010;8(4):231-45. doi: 10.1080/15402002.2010.509247. PubMed PMID: 20924836.

33.	Besser LM, Chang LC, Evenson KR, Hirsch JA, Michael YL, Galvin JE, et al. Associations Between Neighborhood Park Access and Longitudinal Change in Cognition in Older Adults: The Multi-Ethnic Study of Atherosclerosis. Journal of Alzheimers Disease. 2021;82(1):221-33. doi: 10.3233/Jad-210370. PubMed PMID: WOS:000670297900016.

34.	Besser LM, Meyer OL, Jones MR, Tran D, Booker M, Mitsova D, et al. Neighborhood segregation and cognitive change: Multi-Ethnic Study of Atherosclerosis. Alzheimers Dement. 2022. Epub 2022/07/24. doi: 10.1002/alz.12705. PubMed PMID: 35869977.

35.	Besser LM, Rodriguez DA, McDonald N, Kukull WA, Fitzpatrick AL, Rapp SR, et al. Neighborhood built environment and cognition in non-demented older adults: The Multi-Ethnic Study of Atherosclerosis. Soc Sci Med. 2018;200:27-35. Epub 2018/01/23. doi: 10.1016/j.socscimed.2018.01.007. PubMed PMID: 29355828.

36.	Bielinski SJ, Berardi C, Decker PA, Larson NB, Bell EJ, Pankow JS, et al. Hepatocyte growth factor demonstrates racial heterogeneity as a biomarker for coronary heart disease. Heart. 2017;103(15):1185-93. doi: 10.1136/heartjnl-2016-310450. PubMed PMID: WOS:000405486600011.

37.	Bild DE, Detrano R, Peterson D, Guerci A, Liu K, Shahar E, et al. Ethnic differences in coronary calcification: the Multi-Ethnic Study of Atherosclerosis (MESA). Circulation. 2005;111(10):1313-20. doi: 10.1161/01.Cir.0000157730.94423.4b. PubMed PMID: 15769774.

38.	Borker PV, Reid M, Sofer T, Butler MP, Azarbarzin A, Wang H, et al. Non-REM Apnea and Hypopnea Duration Varies across Population Groups and Physiologic Traits. Am J Respir Crit Care Med. 2021;203(9):1173-82. doi: 10.1164/rccm.202005-1808OC. PubMed PMID: 33285084; PubMed Central PMCID: PMCPMC8314913.

39.	Borrell LN, Diez Roux AV, Jacobs DR, Jr., Shea S, Jackson SA, Shrager S, et al. Perceived racial/ethnic discrimination, smoking and alcohol consumption in the Multi-Ethnic Study of Atherosclerosis (MESA). Prev Med. 2010;51(3-4):307-12. Epub 20100608. doi: 10.1016/j.ypmed.2010.05.017. PubMed PMID: 20609433; PubMed Central PMCID: PMCPMC2939242.

40.	Boykin S, Diez-Roux AV, Carnethon M, Shrager S, Ni H, Whitt-Glover M. Racial/ethnic heterogeneity in the socioeconomic patterning of CVD risk factors: in the United States: the multi-ethnic study of atherosclerosis. J Health Care Poor Underserved. 2011;22(1):111-27. doi: 10.1353/hpu.2011.0001. PubMed PMID: 21317510; PubMed Central PMCID: PMCPMC3312013.

41.	Brenner AB, Borrell LN, Barrientos-Gutierrez T, Diez Roux AV. Longitudinal associations of neighborhood socioeconomic characteristics and alcohol availability on drinking: Results from the Multi-Ethnic Study of Atherosclerosis (MESA). Soc Sci Med. 2015;145:17-25. Epub 20150930. doi: 10.1016/j.socscimed.2015.09.030. PubMed PMID: 26439763; PubMed Central PMCID: PMCPMC4630120.

42.	Brenner AB, Diez Roux AV, Barrientos-Gutierrez T, Borrell LN. Association of Alcohol Availability and Neighborhood Socioeconomic Characteristics With Drinking: Cross-Sectional Results From the Multi-Ethnic Study of Atherosclerosis (MESA. Subst Use Misuse. 2015;50(12):1606–17.

43.	Budoff MJ, Nasir K, McClelland RL, Detrano R, Wong N, Blumenthal RS, et al. Coronary Calcium Predicts Events Better With Absolute Calcium Scores Than Age-Sex-Race/Ethnicity Percentiles: MESA (Multi-Ethnic Study of Atherosclerosis. J Am Coll Cardiol. 2009;53(4):345–52.

44.	Cameron NA, Petito LC, McCabe M, Allen NB, O'Brien MJ, Carnethon MR, et al. Quantifying the Sex-Race/Ethnicity-Specific Burden of Obesity on Incident Diabetes Mellitus in the United States, 2001 to 2016: MESA and NHANES. J Am Heart Assoc. 2021;10(4):e018799. Epub 20210210. doi: 10.1161/jaha.120.018799. PubMed PMID: 33563002; PubMed Central PMCID: PMCPMC7955335.

45.	Cao J, Steffen BT, Budoff M, Post WS, Thanassoulis G, Kestenbaum B, et al. Lipoprotein(a) Levels Are Associated With Subclinical Calcific Aortic Valve Disease in White and Black Individuals: The Multi-Ethnic Study of Atherosclerosis. Arterioscler Thromb Vasc Biol. 2016;36(5):1003-9. Epub 20160303. doi: 10.1161/atvbaha.115.306683. PubMed PMID: 26941019; PubMed Central PMCID: PMCPMC4850093.

46.	Carnethon MR, Bertoni AG, Shea S, Greenland P, Ni HY, Jacobs DR, et al. Racial/ethnic differences in subclinical atherosclerosis among adults with diabetes - The multiethnic study of atherosclerosis. Diabetes Care. 2005;28(11):2768-70. doi: DOI 10.2337/diacare.28.11.2768. PubMed PMID: WOS:000234548300028.

47.	Carroll JE, Diez-Roux AV, Adler NE, Seeman TE. Socioeconomic factors and leukocyte telomere length in a multi-ethnic sample: findings from the multi-ethnic study of atherosclerosis (MESA). Brain Behav Immun. 2013;28:108-14. Epub 20121109. doi: 10.1016/j.bbi.2012.10.024. PubMed PMID: 23142704; PubMed Central PMCID: PMCPMC3544984.

48.	Carson AP, Howard G, Burke GL, Shea S, Levitan EB, Muntner P. Ethnic Differences in Hypertension Incidence Among Middle-Aged and Older Adults The Multi-Ethnic Study of Atherosclerosis. Hypertension. 2011;57(6):1101-7. doi: 10.1161/Hypertensionaha.110.168005. PubMed PMID: WOS:000290710800022.

49.	Casillas A, Leng M, Liu K, Hernandez A, Shrager S, Kanaya A. A Long Way from Home: Comparing Mental Health Measures between Foreign and U.S. –born Latinos in the Multi-Ethnic Study of Atherosclerosis (MESA. J Health Care Poor Undeserved. 2012;23(4):1719–32.

50.	Castro-Diehl C, Diez Roux AV, Seeman T, Shea S, Shrager S, Tadros S. Associations of socioeconomic and psychosocial factors with urinary measures of cortisol and catecholamines in the Multi-Ethnic Study of Atherosclerosis (MESA). Psychoneuroendocrinology. 2014;41:132-41. Epub 20131227. doi: 10.1016/j.psyneuen.2013.12.013. PubMed PMID: 24495614; PubMed Central PMCID: PMCPMC3985093.

51.	Charles LE, Fekedulegn D, Burchfiel CM, Fujishiro K, Al Hazzouri AZ, Fitzpatrick AL, et al. Work Hours and Cognitive Function: The Multi-Ethnic Study of Atherosclerosis. Saf Health Work. 2020;11(2):178-86. Epub 20200423. doi: 10.1016/j.shaw.2020.02.004. PubMed PMID: 32596013; PubMed Central PMCID: PMCPMC7303534.

52.	Charles LE, Fekedulegn D, Burchfiel CM, Fujishiro K, Landsbergis P, Diez Roux AV, et al. Associations of work hours with carotid intima-media thickness and ankle-brachial index: the Multi-Ethnic Study of Atherosclerosis (MESA). Occup Environ Med. 2012;69(10):713-20. Epub 20120705. doi: 10.1136/oemed-2012-100665. PubMed PMID: 22767870; PubMed Central PMCID: PMCPMC4084707.

53.	Charles LE, Fekedulegn D, Landsbergis P, Burchfiel CM, Baron S, Kaufman JD, et al. Associations of work hours, job strain, and occupation with endothelial function: the Multi-Ethnic Study of Atherosclerosis (MESA). J Occup Environ Med. 2014;56(11):1153-60. doi: 10.1097/jom.0000000000000311. PubMed PMID: 25376409; PubMed Central PMCID: PMCPMC4225634.

54.	Chen X, Wang R, Zee P, Lutsey PL, Javaheri S, Alcántara C, et al. Racial/Ethnic Differences in Sleep Disturbances: The Multi-Ethnic Study of Atherosclerosis (MESA). Sleep. 2015;38(6):877-88. Epub 20150601. doi: 10.5665/sleep.4732. PubMed PMID: 25409106; PubMed Central PMCID: PMCPMC4434554.

55.	Chung J, Goodman M, Huang T, Wallace ML, Johnson DA, Bertisch S, et al. Racial-ethnic Differences in Actigraphy, Questionnaire, and Polysomnography Indicators of Healthy Sleep: The Multi-Ethnic Study of Atherosclerosis. Am J Epidemiol. 2021. Epub 20210909. doi: 10.1093/aje/kwab232. PubMed PMID: 34498675.

56.	Czarny MJ, Shah SJ, Whelton SP, Blaha MJ, Tsai MY, Denis R, et al. Race/Ethnicity and Prevalence of Aortic Stenosis by Echocardiography in the Multi-Ethnic Study of Atherosclerosis. J Am Coll Cardiol. 2021;78(2):195-7. Epub 20210511. doi: 10.1016/j.jacc.2021.04.078. PubMed PMID: 33989712; PubMed Central PMCID: PMCPMC8282359.

57.	D'Souza JC, Kawut SM, Elkayam LR, Sheppard L, Thorne PS, Jacobs DR, Jr., et al. Ambient Coarse Particulate Matter and the Right Ventricle: The Multi-Ethnic Study of Atherosclerosis. Environ Health Perspect. 2017;125(7):077019. Epub 20170727. doi: 10.1289/ehp658. PubMed PMID: 28760719; PubMed Central PMCID: PMCPMC5744657.

58.	Daniel GD, Chen H, Bertoni AG, Hughes TM, Hayden KM. High visit-to-visit blood pressure variability predicts global cognitive decline: The Multi-Ethnic Study of Atherosclerosis. Alzheimers Dement (N Y). 2022;8(1):e12342. Epub 20220725. doi: 10.1002/trc2.12342. PubMed PMID: 35898668; PubMed Central PMCID: PMCPMC9310191.

59.	Day EC, Li Y, Diez-Roux A, Kandula N, Moran A, Rosas S, et al. Associations of acculturation and kidney dysfunction among Hispanics and Chinese from the Multi-Ethnic Study of Atherosclerosis (MESA. Nephrol Dial Transplant. 2011;26(6):1909–16.

60.	De Moraes ACF, Carvalho HB, McClelland RL, Diez-Roux AV, Szklo M. Sex and ethnicity modify the associations between individual and contextual socioeconomic indicators and ideal cardiovascular health: MESA study. J Public Health (Oxf). 2019;41(3):e237-e44. doi: 10.1093/pubmed/fdy145. PubMed PMID: 30137558.

61.	DeFilippis AP, Young R, McEvoy JW, Michos ED, Sandfort V, Kronmal RA, et al. Risk score overestimation: the impact of individual cardiovascular risk factors and preventive therapies on the performance of the American Heart Association-American College of Cardiology-Atherosclerotic Cardiovascular Disease risk score in a modern multi-ethnic cohort. Eur Heart J. 2017;38(8):598-608. doi: 10.1093/eurheartj/ehw301. PubMed PMID: 27436865; PubMed Central PMCID: PMCPMC5837662.

62.	Delaney JA, Biggs ML, Kronmal RA, Psaty BM. Demographic, medical, and behavioral characteristics associated with over the counter non-sterodial anti-inflammatory drug use in a population-based cohort: results from the Multi-Ethnic Study of Atherosclerosis. Pharmacoepidemiol Drug Saf. 2011;20(1):83–9.

63.	Delaney JA, Oddson BE, McClelland RL, Psaty BM. Estimating ethnic differences in self-reported new use of antidepressant medications: results from the Multi-Ethnic Study of Atherosclerosis. Pharmacoepidemiol Drug Saf. 2009;18(7):545-53. doi: 10.1002/pds.1751. PubMed PMID: 19399919; PubMed Central PMCID: PMCPMC2844249.

64.	Detrano R, Guerci AD, Carr JJ, Bild DE, Burke G, Folsom AR, et al. Coronary calcium as a predictor of coronary events in four racial or ethnic groups. New England Journal of Medicine. 2008;358(13):1336-45. doi: DOI 10.1056/NEJMoa072100. PubMed PMID: WOS:000254308400004.

65.	Diep CS, Lemaitre RN, Chen TA, Baranowski T, Lutsey PL, Manichaikul AW, et al. Acculturation and Plasma Fatty Acid Concentrations in Hispanic and Chinese-American Adults: The Multi-Ethnic Study of Atherosclerosis. PLoS One. 2016;11(2):e0149267. Epub 20160212. doi: 10.1371/journal.pone.0149267. PubMed PMID: 26872329; PubMed Central PMCID: PMCPMC4752232.

66.	Diez Roux AV, Auchincloss AH, Franklin TG, Raghunathan T, Barr RG, Kaufman J, et al. Long-term Exposure to Ambient Particulate Matter and Prevalence of Subclinical Atherosclerosis in the Multi-Ethnic Study of Atherosclerosis. Am J Epidemiol. 2008;167(6):667–75.

67.	Diez Roux AV, Detrano R, Jackson S, Jacobs Jr DR, Schreiner PJ, Shea S, et al. Acculturation and socioeconomic position as predictors of coronary calcification in a multiethnic sample. Circulation. 2005;112(11):1557–65.

68.	Diez Roux AV, Evenson KR, McGinn AP, Brown DG, Moore L, Brines S, et al. Availability of recreational resources and physical activity in adults. Am J Public Health. 2007;97(3):493–9.

69.	Diez Roux AV, Ranjit N, Jenny NS, Shea S, Cushman M, Fitzpatrick A, et al. Race/ethnicity and telomere length in the Multi-Ethnic Study of Atherosclerosis. Aging Cell. 2009;8(3):251–7.

70.	Do DP, Moore K, Barber S, Diez Roux A. Neighborhood racial/ethnic segregation and BMI: A longitudinal analysis of the Multi-ethnic Study of Atherosclerosis. Int J Obes (Lond). 2019;43(8):1601-10. Epub 2019/01/24. doi: 10.1038/s41366-019-0322-3. PubMed PMID: 30670849; PubMed Central PMCID: PMCPMC6646102.

71.	Dubin R, Shlipak M, Li Y, Ix J, de Boer IH, Jenny N, et al. Racial differences in the association of pentraxin-3 with kidney dysfunction: the Multi-Ethnic Study of Atherosclerosis. Nephrol Dial Transplant. 2011;26(6):1903-8. Epub 20101115. doi: 10.1093/ndt/gfq648. PubMed PMID: 21079193; PubMed Central PMCID: PMCPMC3145399.

72.	Duprez DA, Dr J, Jr., Lutsey PL, Herrington D, Prime D, Ouyang P, et al. Race/ethnic and sex differences in large and small artery elasticity -- results of the multi-ethnic study of atherosclerosis (MESA. Ethn Dis. 2009;19(3):243–50.

73.	Eamranond PP, Legedza ATR, Diez-Roux AV, Kandula NR, Palmas W, Siscovick DS, et al. Association between language and risk factor levels among Hispanic adults with hypertension, hypercholesterolemia, or diabetes. American Heart Journal. 2009;157(1):53-9. doi: 10.1016/j.ahj.2008.08.015. PubMed PMID: WOS:000261851700007.

74.	Echeverría S, Diez-Roux AV, Shea S, Borrell LN, Jackson S. Associations of neighborhood problems and neighborhood social cohesion with mental health and health behaviors: the Multi-Ethnic Study of Atherosclerosis. Health Place. 2008;14(4):853-65. Epub 20080126. doi: 10.1016/j.healthplace.2008.01.004. PubMed PMID: 18328772.

75.	Effoe VS, Chen HY, Moran A, Bertoni AG, Bluemke DA, Seeman T, et al. Acculturation is associated with left ventricular mass in a multiethnic sample: the Multi-Ethnic Study of Atherosclerosis. Bmc Cardiovascular Disorders. 2015;15(161). doi: ARTN 161 10.1186/s12872-015-0157-3. PubMed PMID: WOS:000365842400002.

76.	El Khoudary SR, Samargandy S, Zeb I, Foster T, de Boer IH, Li D, et al. Serum 25-hydroxyvitamin-D and nonalcoholic fatty liver disease: Does race/ethnicity matter? Findings from the MESA cohort. Nutr Metab Cardiovasc Dis. 2020;30(1):114–22.

77.	Elmaleh-Sachs A, Balte P, Oelsner EC, Allen NB, Baugh A, Bertoni AG, et al. Race/Ethnicity, Spirometry Reference Equations, and Prediction of Incident Clinical Events: The Multi-Ethnic Study of Atherosclerosis (MESA) Lung Study. Am J Respir Crit Care Med. 2022;205(6):700-10. doi: 10.1164/rccm.202107-1612OC. PubMed PMID: 34913853.

78.	Elmariah S, Budoff MJ, Delaney JA, Hamirani Y, Eng J, Fuster V, et al. Risk factors associated with the incidence and progression of mitral annulus calcification: the multi-ethnic study of atherosclerosis. Am Heart J. 2013;166(5):904-12. Epub 20130926. doi: 10.1016/j.ahj.2013.08.015. PubMed PMID: 24176447; PubMed Central PMCID: PMCPMC3978772.

79.	Everson-Rose SA, Lutsey PL, Roetker NS, Lewis TT, Kershaw KN, Alonso A, et al. Perceived Discrimination and Incident Cardiovascular Events: The Multi-Ethnic Study of Atherosclerosis. Am J Epidemiol. 2015;182(3):225-34. Epub 20150617. doi: 10.1093/aje/kwv035. PubMed PMID: 26085044; PubMed Central PMCID: PMCPMC4517694.

80.	Feinstein M, Ning H, Kang J, Bertoni A, Carnethon M, Lloyd-Jones DM. Racial differences in risks for first cardiovascular events and noncardiovascular death: the Atherosclerosis Risk in Communities study, the Cardiovascular Health Study, and the Multi-Ethnic Study of Atherosclerosis. Circulation. 2012;126(1):50-9. Epub 20120612. doi: 10.1161/circulationaha.111.057232. PubMed PMID: 22693351; PubMed Central PMCID: PMCPMC3437934.

81.	Fernandes VR, Cheng S, Cheng YJ, Rosen B, Agarwal S, McClelland RL, et al. Racial and ethnic differences in subclinical myocardial function: the Multi-Ethnic Study of Atherosclerosis. Heart. 2011;97(5):405-10. Epub 20110121. doi: 10.1136/hrt.2010.209452. PubMed PMID: 21258000; PubMed Central PMCID: PMCPMC3034784.

82.	Fisher DE, Shrager S, Shea SJ, Burke GL, Klein R, Wong TY, et al. Visual Impairment in White, Chinese, Black, and Hispanic Participants from the Multi-Ethnic Study of Atherosclerosis Cohort. Ophthalmic Epidemiol. 2015;22(5):321-32. doi: 10.3109/09286586.2015.1066395. PubMed PMID: 26395659; PubMed Central PMCID: PMCPMC4768912.

83.	Fitzpatrick AL, Rapp SR, Luchsinger J, Hill-Briggs F, Alonso A, Gottesman R, et al. Sociodemographic Correlates of Cognition in the Multi-Ethnic Study of Atherosclerosis (MESA). Am J Geriatr Psychiatry. 2015;23(7):684-97. Epub 20150120. doi: 10.1016/j.jagp.2015.01.003. PubMed PMID: 25704999; PubMed Central PMCID: PMCPMC4465027.

84.	Fleishman MW, Budoff M, Zeb I, Li D, Foster T. NAFLD prevalence differs among hispanic subgroups: The multi-ethnic study of atherosclerosis. World J Gastroenterol. 2014;20(17):4987–93.

85.	Flores Rosario K, Mehta A, Ayers C, Engel Gonzalez P, Pandey A, Khera R, et al. Performance of the Pooled Cohort Equations in Hispanic Individuals Across the United States: Insights From the Multi-Ethnic Study of Atherosclerosis and the Dallas Heart Study. J Am Heart Assoc. 2021;10(9):e018410. Epub 20210417. doi: 10.1161/jaha.120.018410. PubMed PMID: 33870702; PubMed Central PMCID: PMCPMC8200750.

86.	Forbang NI, Criqui MH, Allison MA, Ix JH, Steffen BT, Cushman M, et al. Sex and ethnic differences in the associations between lipoprotein(a) and peripheral arterial disease in the Multi-Ethnic Study of Atherosclerosis. Journal of Vascular Surgery. 2016;63(2):453-8. doi: 10.1016/j.jvs.2015.08.114. PubMed PMID: WOS:000370779100027.

87.	Forde AT, Lewis TT, Kershaw KN, Bellamy SL, Diez Roux AV. Perceived Discrimination and Hypertension Risk Among Participants in the Multi-Ethnic Study of Atherosclerosis. J Am Heart Assoc. 2021;10(5):e019541. Epub 20210218. doi: 10.1161/jaha.120.019541. PubMed PMID: 33596667; PubMed Central PMCID: PMCPMC8174295.

88.	Foster MC, Levey AS, Inker LA, Shafi T, Fan L, Gudnason V, et al. Non-GFR Determinants of Low-Molecular-Weight Serum Protein Filtration Markers in the Elderly: AGES-Kidney and MESA-Kidney. Am J Kidney Dis. 2017;70(3):406-14. Epub 20170524. doi: 10.1053/j.ajkd.2017.03.021. PubMed PMID: 28549536; PubMed Central PMCID: PMCPMC5572311.

89.	Foster T, Anania FA, Li D, Katz R, Budoff M. The prevalence and clinical correlates of nonalcoholic fatty liver disease (NAFLD) in African Americans: the multiethnic study of atherosclerosis (MESA). Dig Dis Sci. 2013;58(8):2392-8. Epub 20130402. doi: 10.1007/s10620-013-2652-7. PubMed PMID: 23546700; PubMed Central PMCID: PMCPMC3780774.

90.	Frazier-Wood AC, Manichaikul A, Aslibekyan S, Borecki IB, Goff DC, Hopkins PN, et al. Genetic variants associated with VLDL, LDL and HDL particle size differ with race/ethnicity. Hum Genet. 2013;132(4):405-13. Epub 20121222. doi: 10.1007/s00439-012-1256-1. PubMed PMID: 23263444; PubMed Central PMCID: PMCPMC3600091.

91.	Frazier-Wood AC, Wang Z, Manichukal A, Dc G, Jr., Mora S, Ordovas JM, et al. Genetic associations with lipoprotein subfraction measures differ by ethnicity in the multi-ethnic study of atherosclerosis (MESA. Hum Genet. 2017;136(6):715–26.

92.	Fujishiro K, Diez Roux AV, Landsbergis P, Kaufman JD, Korcarz CE, Stein JH. Occupational characteristics and the progression of carotid artery intima-media thickness and plaque over 9 years: the Multi-Ethnic Study of Atherosclerosis (MESA). Occup Environ Med. 2015;72(10):690-8. Epub 20140912. doi: 10.1136/oemed-2014-102311. PubMed PMID: 25217203; PubMed Central PMCID: PMCPMC4560665.

93.	Fujishiro K, Hajat A, Landsbergis PA, Meyer JD, Schreiner PJ, Kaufman JD. Explaining racial/ethnic differences in all-cause mortality in the Multi-Ethnic Study of Atherosclerosis (MESA): Substantive complexity and hazardous working conditions as mediating factors. SSM Popul Health. 2017;3:497–505.

94.	Fujishiro K, Landsbergis PA, Diez-Roux AV, Stukovsky KH, Shrager S, Baron S. Factorial invariance, scale reliability, and construct validity of the job control and job demands scales for immigrant workers: the multi-ethnic study of atherosclerosis. J Immigr Minor Health. 2011;13(3):533-40. doi: 10.1007/s10903-010-9364-2. PubMed PMID: 20582720; PubMed Central PMCID: PMCPMC4068014.

95.	Fujishiro K, Needham BL, Landsbergis PA, Seeman T, Jenny NS, Diez Roux AV. Selected occupational characteristics and change in leukocyte telomere length over 10 years: The Multi-Ethnic Study of Atherosclerosis (MESA). PLoS One. 2018;13(9):e0204704. Epub 20180927. doi: 10.1371/journal.pone.0204704. PubMed PMID: 30261026; PubMed Central PMCID: PMCPMC6160145.

96.	Fujiyoshi A, Miura K, Ohkubo T, Kadowaki T, Kadowaki S, Zaid M, et al. Cross-Sectional Comparison of Coronary Artery Calcium Scores Between Caucasian Men in the United States and Japanese Men in Japan The Multi-Ethnic Study of Atherosclerosis and the Shiga Epidemiological Study of Subclinical Atherosclerosis. American Journal of Epidemiology. 2014;180(6):590-8. doi: 10.1093/aje/kwu169. PubMed PMID: WOS:000343033400006.

97.	Gallo LC, Los Monteros KE, Allison M, Roux AD, Polak JF, Watson KE, et al. Do socioeconomic gradients in subclinical atherosclerosis vary according to acculturation level? Analyses of mexican-americans in the multi-ethnic study of atherosclerosis. Psychosom Med. 2009;71(7):756–62.

98.	Gao SK, Fitzpatrick AL, Psaty B, Jiang R, Post W, Cutler J, et al. Suboptimal nutritional intake for hypertension control in 4 ethnic groups. Arch Intern Med. 2009;169(7):702-7. doi: 10.1001/archinternmed.2009.17. PubMed PMID: 19365000.

99.	Gao X, Engeda J, Moore LV, Auchincloss AH, Moore K, Mujahid MS. Longitudinal associations between objective and perceived healthy food environment and diet: The Multi-Ethnic Study of Atherosclerosis. Soc Sci Med. 2022;292(114542):114542. doi: 10.1016/j.socscimed.2021.114542. PubMed PMID: 34802783.

100.	Gao X, Kershaw KN, Barber S, Schreiner PJ, Do DP, Diez Roux AV, et al. Associations Between Residential Segregation and Incident Hypertension: The Multi-Ethnic Study of Atherosclerosis. J Am Heart Assoc. 2022;11(3):e023084. Epub 20220120. doi: 10.1161/jaha.121.023084. PubMed PMID: 35048712; PubMed Central PMCID: PMCPMC9238487.

101.	Garg SK, Lin F, Kandula N, Ding J, Carr J, Allison M, et al. Ectopic Fat Depots and Coronary Artery Calcium in South Asians Compared With Other Race/Ethnic Groups. J Am Heart Assoc. 2016;5(11).

102.	Gijsberts CM, Groenewegen KA, Hoefer IE, Eijkemans MJ, Asselbergs FW, Anderson TJ, et al. Race/Ethnic Differences in the Associations of the Framingham Risk Factors with Carotid IMT and Cardiovascular Events. PLoS One. 2015;10(7):e0132321. Epub 2015/07/03. doi: 10.1371/journal.pone.0132321. PubMed PMID: 26134404; PubMed Central PMCID: PMCPMC4489855.

103.	Goff DC, Jr., Bertoni AG, Kramer H, Bonds D, Blumenthal RS, Tsai MY, et al. Dyslipidemia prevalence, treatment, and control in the Multi-Ethnic Study of Atherosclerosis (MESA): gender, ethnicity, and coronary artery calcium. Circulation. 2006;113(5):647-56. doi: 10.1161/circulationaha.105.552737. PubMed PMID: 16461837.

104.	Guan WH, Cao J, Steffen BT, Post WS, Stein JH, Tattersall MC, et al. Race Is a Key Variable in Assigning Lipoprotein(a) Cutoff Values for Coronary Heart Disease Risk Assessment The Multi-Ethnic Study of Atherosclerosis. Arteriosclerosis Thrombosis and Vascular Biology. 2015;35(4):996-1001. doi: 10.1161/Atvbaha.114.304785. PubMed PMID: WOS:000351709200032.

105.	Gujral UP, Vittinghoff E, Mongraw-Chaffin M, Vaidya D, Kandula NR, Allison M, et al. Cardiometabolic Abnormalities Among Normal-Weight Persons From Five Racial/Ethnic Groups in the United States A Cross-sectional Analysis of Two Cohort Studies. Annals of Internal Medicine. 2017;166(9):628-+. doi: 10.7326/M16-1895. PubMed PMID: WOS:000400395500007.

106.	Gupta DK, Daniels LB, Cheng S, deFilippi CR, Criqui MH, Maisel AS, et al. Differences in Natriuretic Peptide Levels by Race/Ethnicity (From the Multi-Ethnic Study of Atherosclerosis). Am J Cardiol. 2017;120(6):1008-15. Epub 20170630. doi: 10.1016/j.amjcard.2017.06.030. PubMed PMID: 28750825; PubMed Central PMCID: PMCPMC5575954.

107.	Gutierrez OM, Sang Y, Grams ME, Ballew SH, Surapaneni A, Matsushita K, et al. Association of Estimated GFR Calculated Using Race-Free Equations With Kidney Failure and Mortality by Black vs Non-Black Race. JAMA. 2022;327(23):2306-16. Epub 2022/06/07. doi: 10.1001/jama.2022.8801. PubMed PMID: 35667006; PubMed Central PMCID: PMCPMC9171658 Akebia, Amgen, and GlaxoSmithKline and personal fees from AstraZeneca, Reata, and Ardelyx, and serving on a data monitoring committee for QED outside the submitted work. Dr Grams reported receiving grants from the National Institutes of Health (NIH) and National Kidney Foundation (NKF) during the conduct of the study; receiving grants from the NIH and NKF and nonfinancial support from Kidney Disease: Improving Global Outcomes (KDIGO) outside the submitted work; and being a member of the USRDS Scientific Advisory Committee and NKF Scientific Advisory Committee and KDIGO Executive Committee. Dr Matsushita reported receiving grants from the NIH during the conduct of the study and personal fees from Akebia and Kyowa Kirin and grants from Kyowa Kirin outside the submitted work. Dr Go reported receiving grants from the National Institute of Diabetes, Digestive, and Kidney Diseases during the conduct of the study. Dr Shlipak reported receiving personal fees from Cricket Health, Intercept Pharmaceuticals, Bayer Pharmaceuticals, AstraZeneca, and Boehringer Ingelheim and grants from Bayer Pharmaceuticals outside the submitted work. Dr Inker reported funding to Tufts Medical Center for research, consulting, or contracts with the NIH, NKF, Reata, Omeros, Chinnocks Tricida, and Goldfinch Bio. Dr Inker also reported receiving consulting fees from Diametrix and being a member of the NKF-American Society of Nephrology (ASN) Task Force on Reassessing the Inclusion of Race in Diagnosing Kidney Disease; the views presented here are hers and the authors' and do not reflect the views of the NKF-ASN Task Force. Dr Crews reported receiving grants from Somatus and Baxter International outside the submitted work and being a member of the NKF-ASN Task Force on Reassessing the Inclusion of Race in Reassessing the Inclusion of Race in Diagnosing Kidney Disease. Dr Levey reported receiving grants from the NIH, NKF (grant support for Chronic Kidney Disease Epidemiology Collaboration and Chronic Kidney Disease Prognosis Consortium [CKD-PC]) during the conduct of the study and personal fees from AstraZeneca for participation in data and safety monitoring boards for dapagliflozin clinical trials. Dr Coresh reported receiving grants from the NIH and NKF during the conduct of the study and being a consultant and owning stock options of Healthy.io outside the submitted work. No other disclosures were reported.

108.	Hajat A, Diez-Roux A, Franklin TG, Seeman T, Shrager S, Ranjit N, et al. Socioeconomic and race/ethnic differences in daily salivary cortisol profiles: the multi-ethnic study of atherosclerosis. Psychoneuroendocrinology. 2010;35(6):932-43. doi: 10.1016/j.psyneuen.2009.12.009. PubMed PMID: 20116177.

109.	Heckbert SR, Austin TR, Jensen PN, Chen LY, Post WS, Floyd JS, et al. Differences by Race/Ethnicity in the Prevalence of Clinically Detected and Monitor-Detected Atrial Fibrillation: MESA. Circ Arrhythm Electrophysiol. 2020;13(1):e007698. Epub 20200114. doi: 10.1161/circep.119.007698. PubMed PMID: 31934795; PubMed Central PMCID: PMCPMC7204495.

110.	Hicken MT, Adar SD, Hajat A, Kershaw KN, Do DP, Barr RG, et al. Air Pollution, Cardiovascular Outcomes, and Social Disadvantage: The Multi-ethnic Study of Atherosclerosis. Epidemiology. 2016;27(1):42-50. doi: 10.1097/ede.0000000000000367. PubMed PMID: 26618771; PubMed Central PMCID: PMCPMC4937221.

111.	Hirsch JA, Michael YL, Moore KA, Melly S, Hughes TM, Hayden K, et al. Longitudinal neighbourhood determinants with cognitive health and dementia disparities: protocol of the Multi-Ethnic Study of Atherosclerosis Neighborhoods and Aging prospective cohort study. BMJ Open. 2022;12(11):e066971. Epub 20221111. doi: 10.1136/bmjopen-2022-066971. PubMed PMID: 36368762; PubMed Central PMCID: PMCPMC9660618.

112.	Hisamatsu T, Liu K, Chan C, Krefman AE, Fujiyoshi A, Budoff MJ, et al. Coronary Artery Calcium Progression Among the US and Japanese Men. Circ Cardiovasc Imaging. 2019;12(2):e008104. Epub 2019/02/14. doi: 10.1161/CIRCIMAGING.118.008104. PubMed PMID: 30755051; PubMed Central PMCID: PMCPMC6482858.

113.	Hsu S, Hoofnagle AN, Gupta DK, Gutierrez OM, Peralta CA, Shea S, et al. Race, Ancestry, and Vitamin D Metabolism: The Multi-Ethnic Study of Atherosclerosis. J Clin Endocrinol Metab. 2020;105(12):e4337-50. doi: 10.1210/clinem/dgaa612. PubMed PMID: 32869845; PubMed Central PMCID: PMCPMC7526733.

114.	Hu J, Yao J, Dseng SL, Balasubramanian R, Jimenez MC, Li J, et al. Differences in Metabolomic Profiles Between Black and White Women and Risk of Coronary Heart Disease: an Observational Study of Women From Four US Cohorts. Circulation Research. 2022;131(7):601-15. doi: 10.1161/Circresaha.121.320134. PubMed PMID: WOS:000853194600006.

115.	Huang CC, Lloyd-Jones DM, Guo XQ, Rajamannan NM, Lin S, Du P, et al. Gene expression variation between African Americans and whites is associated with coronary artery calcification: the multiethnic study of atherosclerosis. Physiological Genomics. 2011;43(13):836-43. doi: 10.1152/physiolgenomics.00243.2010. PubMed PMID: WOS:000292735600006.

116.	Hussein M, Diez Roux AV, Mujahid MS, Hastert TA, Kershaw KN, Bertoni AG, et al. Unequal Exposure or Unequal Vulnerability? Contributions of Neighborhood Conditions and Cardiovascular Risk Factors to Socioeconomic Inequality in Incident Cardiovascular Disease in the Multi-Ethnic Study of Atherosclerosis. Am J Epidemiol. 2018;187(7):1424-37. doi: 10.1093/aje/kwx363. PubMed PMID: 29186311; PubMed Central PMCID: PMCPMC6030985.

117.	Inker LA, Levey AS, Tighiouart H, Shafi T, Eckfeldt JH, Johnson C, et al. Performance of glomerular filtration rate estimating equations in a community-based sample of Blacks and Whites: the multiethnic study of atherosclerosis. Nephrol Dial Transplant. 2018;33(3):417-25. doi: 10.1093/ndt/gfx042. PubMed PMID: 28505377; PubMed Central PMCID: PMCPMC6018782.

118.	Inker LA, Shafi T, Okparavero A, Tighiouart H, Eckfeldt JH, Katz R, et al. Effects of Race and Sex on Measured GFR: The Multi-Ethnic Study of Atherosclerosis. Am J Kidney Dis. 2016;68(5):743-51. Epub 20160820. doi: 10.1053/j.ajkd.2016.06.021. PubMed PMID: 27555103.

119.	Inoue K, Horwich T, Bhatnagar R, Bhatt K, Goldwater D, Seeman T, et al. Urinary Stress Hormones, Hypertension, and Cardiovascular Events: The Multi-Ethnic Study of Atherosclerosis. Hypertension. 2021;78(5):1640-7. Epub 20210913. doi: 10.1161/hypertensionaha.121.17618. PubMed PMID: 34510914.

120.	Jackson CL, Patel SR, Jackson WB, 2nd, Lutsey PL, Redline S. Agreement between self-reported and objectively measured sleep duration among white, black, Hispanic, and Chinese adults in the United States: Multi-Ethnic Study of Atherosclerosis. Sleep. 2018;41(6). doi: 10.1093/sleep/zsy057. PubMed PMID: 29701831; PubMed Central PMCID: PMCPMC5995218.

121.	Jain A, Tandri H, Dalal D, Chahal H, Soliman EZ, Prineas RJ, et al. Diagnostic and prognostic utility of electrocardiography for left ventricular hypertrophy defined by magnetic resonance imaging in relationship to ethnicity: the Multi-Ethnic Study of Atherosclerosis (MESA). Am Heart J. 2010;159(4):652-8. doi: 10.1016/j.ahj.2009.12.035. PubMed PMID: 20362725; PubMed Central PMCID: PMCPMC2856691.

122.	Javaheri S, Sharma RK, Wang R, Weng J, Rosen BD, Bluemke DA, et al. Association between Obstructive Sleep Apnea and Left Ventricular Structure by Age and Gender: the Multi-Ethnic Study of Atherosclerosis. Sleep. 2016;39(3):523-9. doi: PII sp-00337-15 10.5665/sleep.5518. PubMed PMID: WOS:000371115800006.

123.	Johnson DA, Hirsch JA, Moore KA, Redline S, Roux AVD. Associations Between the Built Environment and Objective Measures of Sleep The Multi-Ethnic Study of Atherosclerosis. American Journal of Epidemiology. 2018;187(5):941-50. doi: 10.1093/aje/kwx302. PubMed PMID: WOS:000432045400007.

124.	Jones MR, Diez-Roux AV, Hajat A, Kershaw KN, O'Neill MS, Guallar E, et al. Race/ethnicity, residential segregation, and exposure to ambient air pollution: the Multi-Ethnic Study of Atherosclerosis (MESA). Am J Public Health. 2014;104(11):2130-7. Epub 20140911. doi: 10.2105/ajph.2014.302135. PubMed PMID: 25211756; PubMed Central PMCID: PMCPMC4202969.

125.	Jones MR, Diez-Roux AV, O'Neill MS, Guallar E, Sharrett AR, Post W, et al. Ambient air pollution and racial/ethnic differences in carotid intima-media thickness in the Multi-Ethnic Study of Atherosclerosis (MESA). J Epidemiol Community Health. 2015;69(12):1191-8. Epub 20150703. doi: 10.1136/jech-2015-205588. PubMed PMID: 26142402; PubMed Central PMCID: PMCPMC4763882.

126.	Jones MR, Tellez-Plaza M, Vaidya D, Grau-Perez M, Post WS, Kaufman JD, et al. Ethnic, geographic and dietary differences in arsenic exposure in the multi-ethnic study of atherosclerosis (MESA). J Expo Sci Environ Epidemiol. 2019;29(3):310-22. Epub 20180524. doi: 10.1038/s41370-018-0042-0. PubMed PMID: 29795237; PubMed Central PMCID: PMCPMC6252166.

127.	Jones SA, Li Q, Aiello AE, O'Rand AM, Evenson KR. Correlates of changes in walking during the retirement transition: The Multi-Ethnic Study of Atherosclerosis. Prev Med Rep. 2018;11:221-30. Epub 20180704. doi: 10.1016/j.pmedr.2018.07.002. PubMed PMID: 30210994; PubMed Central PMCID: PMCPMC6129965.

128.	Joseph JJ, Echouffo-Tcheugui JB, Golden SH, Chen H, Jenny NS, Carnethon MR, et al. Physical activity, sedentary behaviors and the incidence of type 2 diabetes mellitus: the Multi-Ethnic Study of Atherosclerosis (MESA). BMJ Open Diabetes Res Care. 2016;4(1):e000185. Epub 20160623. doi: 10.1136/bmjdrc-2015-000185. PubMed PMID: 27403323; PubMed Central PMCID: PMCPMC4932325.

129.	Kaiser P, Peralta CA, Kronmal R, Shlipak MG, Psaty BM, Odden MC. Racial/ethnic heterogeneity in associations of blood pressure and incident cardiovascular disease by functional status in a prospective cohort: the Multi-Ethnic Study of Atherosclerosis. BMJ Open. 2018;8(2):e017746. Epub 20180223. doi: 10.1136/bmjopen-2017-017746. PubMed PMID: 29476026; PubMed Central PMCID: PMCPMC5855482.

130.	Kalyani RR, Lazo M, Ouyang P, Turkbey E, Chevalier K, Brancati F, et al. Sex differences in diabetes and risk of incident coronary artery disease in healthy young and middle-aged adults. Diabetes Care. 2014;37(3):830-8. Epub 20131031. doi: 10.2337/dc13-1755. PubMed PMID: 24178997; PubMed Central PMCID: PMCPMC3931379.

131.	Kanaya AM, Dobrosielski DA, Ganz P, Creasman J, Gupta R, Nelacanti V, et al. Glycemic associations with endothelial function and biomarkers among 5 ethnic groups: the Multi-Ethnic Study of Atherosclerosis and the Mediators of Atherosclerosis in South Asians Living in America studies. J Am Heart Assoc. 2013;2(1):e004283. Epub 20130206. doi: 10.1161/jaha.112.004283. PubMed PMID: 23525433; PubMed Central PMCID: PMCPMC3603246.

132.	Kanaya AM, Herrington D, Vittinghoff E, Ewing SK, Liu K, Blaha MJ, et al. Understanding the high prevalence of diabetes in U.S. south Asians compared with four racial/ethnic groups: the MASALA and MESA studies. Diabetes Care. 2014;37(6):1621-8. Epub 20140404. doi: 10.2337/dc13-2656. PubMed PMID: 24705613; PubMed Central PMCID: PMCPMC4030091.

133.	Kanaya AM, Kandula NR, Ewing SK, Herrington D, Liu K, Blaha MJ, et al. Comparing coronary artery calcium among U.S. South Asians with four racial/ethnic groups: The MASALA and MESA studies (vol 234, pg 102, 2014). Atherosclerosis. 2014;235(1):36-7. doi: 10.1016/j.atherosclerosis.2014.04.017. PubMed PMID: WOS:000339395500005.

134.	Kanaya AM, Vittinghoff E, Lin F, Kandula NR, Herrington D, Liu K, et al. Incidence and Progression of Coronary Artery Calcium in South Asians Compared With 4 Race/Ethnic Groups. J Am Heart Assoc. 2019;8(2):e011053. doi: 10.1161/jaha.118.011053. PubMed PMID: 30630376; PubMed Central PMCID: PMCPMC6497354.

135.	Kanaya AM, Wassel CL, Mathur D, Stewart A, Herrington D, Budoff MJ, et al. Prevalence and correlates of diabetes in South asian indians in the United States: findings from the metabolic syndrome and atherosclerosis in South asians living in america study and the multi-ethnic study of atherosclerosis. Metab Syndr Relat Disord. 2010;8(2):157-64. doi: 10.1089/met.2009.0062. PubMed PMID: 19943798; PubMed Central PMCID: PMCPMC3139526.

136.	Kandula NR, Diez-Roux AV, Chan C, Daviglus ML, Jackson SA, Ni H, et al. Association of acculturation levels and prevalence of diabetes in the multi-ethnic study of atherosclerosis (MESA). Diabetes Care. 2008;31(8):1621-8. Epub 20080505. doi: 10.2337/dc07-2182. PubMed PMID: 18458142; PubMed Central PMCID: PMCPMC2494621.

137.	Kawut SM, Lima JA, Barr RG, Chahal H, Jain A, Tandri H, et al. Sex and race differences in right ventricular structure and function: the multi-ethnic study of atherosclerosis-right ventricle study. Circulation. 2011;123(22):2542-51. doi: 10.1161/circulationaha.110.985515. PubMed PMID: 21646505; PubMed Central PMCID: PMCPMC3111939.

138.	Kershaw KN, Diez Roux AV, Bertoni A, Carnethon MR, Everson-Rose SA, Liu K. Associations of chronic individual-level and neighbourhood-level stressors with incident coronary heart disease: the Multi-Ethnic Study of Atherosclerosis. J Epidemiol Community Health. 2015;69(2):136-41. Epub 20140930. doi: 10.1136/jech-2014-204217. PubMed PMID: 25271247; PubMed Central PMCID: PMCPMC4806779.

139.	Kershaw KN, Osypuk TL, Do DP, De Chavez PJ, Diez Roux AV. Neighborhood-level racial/ethnic residential segregation and incident cardiovascular disease: the multi-ethnic study of atherosclerosis. Circulation. 2015;131(2):141-8. Epub 2014/12/03. doi: 10.1161/CIRCULATIONAHA.114.011345. PubMed PMID: 25447044; PubMed Central PMCID: PMCPMC4293329.

140.	Kershaw KN, Roux AVD, Carnethon M, Darwin C, Goff DC, Post W, et al. Geographic Variation in Hypertension Prevalence Among Blacks and Whites: The Multi-Ethnic Study of Atherosclerosis. American Journal of Hypertension. 2010;23(1):46-53. doi: 10.1038/ajh.2009.211. PubMed PMID: WOS:000272831400008.

141.	Kianoush S, DeFilippis AP, Rodriguez CJ, Al Rifai M, Benjamin EJ, Hall ME, et al. Race/Ethnicity-Specific Associations between Smoking, Serum Leptin, and Abdominal Fat: The Multi-Ethnic Study of Atherosclerosis. Ethnicity & Disease. 2018;28(4):531-8. doi: 10.18865/ed.28.4.531. PubMed PMID: WOS:000458403700004.

142.	Kim C, Diez-Roux AV, Nettleton JA, Polak JF, Post WS, Siscovick DS, et al. Sex differences in subclinical atherosclerosis by race/ethnicity in the multi-ethnic study of atherosclerosis. Am J Epidemiol. 2011;174(2):165-72. Epub 20110617. doi: 10.1093/aje/kwr088. PubMed PMID: 21685409; PubMed Central PMCID: PMCPMC3167681.

143.	Kim JY, Choi J, Vella CA, Criqui MH, Allison MA, Kim NH. Associations between Weight-Adjusted Waist Index and Abdominal Fat and Muscle Mass: Multi-Ethnic Study of Atherosclerosis. Diabetes & Metabolism Journal. 2022;46(5):747-55. doi: 10.4093/dmj.2021.0294. PubMed PMID: WOS:000910538100009.

144.	Klein R, Klein BE, Knudtson MD, Wong TY, Cotch MF, Liu K, et al. Prevalence of age-related macular degeneration in 4 racial/ethnic groups in the multi-ethnic study of atherosclerosis. Ophthalmology. 2006;113(3):373-80. doi: 10.1016/j.ophtha.2005.12.013. PubMed PMID: 16513455.

145.	Klein R, Li XH, Kuo JZ, Klein BEK, Cotch MF, Wong TY, et al. Associations of Candidate Genes to Age-Related Macular Degeneration Among Racial/Ethnic Groups in the Multi-Ethnic Study of Atherosclerosis. American Journal of Ophthalmology. 2013;156(5):1010-20. doi: 10.1016/j.ajo.2013.06.004. PubMed PMID: WOS:000326859200021.

146.	Koska J, Furtado J, Hu Y, Sinari S, Budoff MJ, Billheimer D, et al. Plasma proteoforms of apolipoproteins C-I and C-II are associated with plasma lipids in the Multi-Ethnic Study of Atherosclerosis. J Lipid Res. 2022;63(9):100263. Epub 20220809. doi: 10.1016/j.jlr.2022.100263. PubMed PMID: 35952903; PubMed Central PMCID: PMCPMC9494236.

147.	Kramer H, Han C, Post W, Goff D, Diez-Roux A, Cooper R, et al. Racial/ethnic differences in hypertension and hypertension treatment and control in the multi-ethnic study of atherosclerosis (MESA). Am J Hypertens. 2004;17(10):963-70. doi: 10.1016/j.amjhyper.2004.06.001. PubMed PMID: 15485761.

148.	Kramer H, Palmas W, Kestenbaum B, Cushman M, Allison M, Astor B, et al. Chronic kidney disease prevalence estimates among racial/ethnic groups: The Multi-Ethnic Study of Atherosclerosis. Clinical Journal of the American Society of Nephrology. 2008;3(5):1391-7. doi: 10.2215/Cjn.04160907. PubMed PMID: WOS:000258757500028.

149.	Landsbergis PA, Diez-Roux AV, Fujishiro K, Baron S, Kaufman JD, Meyer JD, et al. Job Strain, Occupational Category, Systolic Blood Pressure, and Hypertension Prevalence: The Multi-Ethnic Study of Atherosclerosis. J Occup Environ Med. 2015;57(11):1178-84. doi: 10.1097/jom.0000000000000533. PubMed PMID: 26539765; PubMed Central PMCID: PMCPMC4636023.

150.	Le-Scherban F, Albrecht SS, Bertoni A, Kandula N, Mehta N, Diez Roux AV. Immigrant status and cardiovascular risk over time: results from the Multi-Ethnic Study of Atherosclerosis. Ann Epidemiol. 2016;26(6):429-35 e1. doi: 10.1016/j.annepidem.2016.04.008. PubMed PMID: 27221804.

151.	Le-Scherban F, Albrecht SS, Osypuk TL, Sanchez BN, Roux AVD. Long-term neighborhood ethnic composition and weight-related outcomes among immigrants: The Multi-Ethnic Study of Atherosclerosis. Health & Place. 2019;58(102147):102147. doi: ARTN 102147 10.1016/j.healthplace.2019.102147. PubMed PMID: WOS:000482101600013.

152.	Levine DA, Gross AL, Briceño EM, Tilton N, Whitney R, Han D, et al. Blood Pressure and Later-Life Cognition in Hispanic and White Adults (BP-COG): A Pooled Cohort Analysis of ARIC, CARDIA, CHS, FOS, MESA, and NOMAS. J Alzheimers Dis. 2022;89(3):1103-17. doi: 10.3233/jad-220366. PubMed PMID: 35964190.

153.	Li AE, Kamel I, Rando F, Anderson M, Kumbasar B, Lima JA, et al. Using MRI to assess aortic wall thickness in the multiethnic study of atherosclerosis: distribution by race, sex, and age. AJR Am J Roentgenol. 2004;182(3):593-7. doi: 10.2214/ajr.182.3.1820593. PubMed PMID: 14975953.

154.	Lin SX, Carnethon M, Szklo M, Bertoni A. Racial/ethnic differences in the association of triglycerides with other metabolic syndrome components: the Multi-Ethnic Study of Atherosclerosis. Metab Syndr Relat Disord. 2011;9(1):35-40. Epub 20101019. doi: 10.1089/met.2010.0050. PubMed PMID: 20958206; PubMed Central PMCID: PMCPMC3125569.

155.	Lutsey PL, Cushman M, Steffen LM, Green D, Barr RG, Herrington D, et al. Plasma hemostatic factors and endothelial markers in four racial/ethnic groups: the MESA study. J Thromb Haemost. 2006;4(12):2629-35. Epub 20060926. doi: 10.1111/j.1538-7836.2006.02237.x. PubMed PMID: 17002663.

156.	Lutsey PL, Diez Roux AV, Jacobs DR, Jr., Burke GL, Harman J, Shea S, et al. Associations of acculturation and socioeconomic status with subclinical cardiovascular disease in the multi-ethnic study of atherosclerosis. Am J Public Health. 2008;98(11):1963-70. Epub 20080529. doi: 10.2105/ajph.2007.123844. PubMed PMID: 18511718; PubMed Central PMCID: PMCPMC2575668.

157.	Lutsey PL, Pereira MA, Bertoni AG, Kandula NR, Jacobs JDR. Interactions Between Race/Ethnicity and Anthropometry in Risk of Incident Diabetes: The Multi-Ethnic Study of Atherosclerosis. Am J Epidemiol. 2010;172(2):197–204.

158.	Mair C, Diez Roux AV, Osypuk TL, Rapp SR, Seeman T, Watson KE. Is neighborhood racial/ethnic composition associated with depressive symptoms? The multi-ethnic study of atherosclerosis. Soc Sci Med. 2010;71(3):541–50.

159.	Manichaikul A, Hoffman EA, Smolonska J, Gao W, Cho MH, Baumhauer H, et al. Genome-wide study of percent emphysema on computed tomography in the general population. The Multi-Ethnic Study of Atherosclerosis Lung/SNP Health Association Resource Study. Am J Respir Crit Care Med. 2014;189(4):408-18. doi: 10.1164/rccm.201306-1061OC. PubMed PMID: 24383474; PubMed Central PMCID: PMCPMC3977717.

160.	Manichaikul A, Rich SS, Allison MA, Guagliardo NA, Bayliss DA, Carey RM, et al. KCNK3 Variants Are Associated With Hyperaldosteronism and Hypertension. Hypertension. 2016;68(2):356-64. Epub 20160613. doi: 10.1161/hypertensionaha.116.07564. PubMed PMID: 27296998; PubMed Central PMCID: PMCPMC4945430.

161.	Manolio TA, Arnold AM, Post W, Bertoni AG, Schreiner PJ, Sacco RL, et al. Ethnic differences in the relationship of carotid atherosclerosis to coronary calcification: the Multi-Ethnic Study of Atherosclerosis. Atherosclerosis. 2008;197(1):132-8. Epub 20070406. doi: 10.1016/j.atherosclerosis.2007.02.030. PubMed PMID: 17412347; PubMed Central PMCID: PMCPMC2314669.

162.	Mayne SL, Auchincloss AH, Stehr MF, Kern DM, Navas-Acien A, Kaufman JD, et al. Longitudinal Associations of Local Cigarette Prices and Smoking Bans with Smoking Behavior in the Multi-Ethnic Study of Atherosclerosis. Epidemiology. 2017;28(6):863-71. doi: 10.1097/ede.0000000000000736. PubMed PMID: 28817468; PubMed Central PMCID: PMCPMC5731651.

163.	Mayne SL, Hicken MT, Merkin SS, Seeman TE, Kershaw KN, Do DP, et al. Neighbourhood racial/ethnic residential segregation and cardiometabolic risk: the multiethnic study of atherosclerosis. J Epidemiol Community Health. 2019;73(1):26-33. Epub 20180929. doi: 10.1136/jech-2018-211159. PubMed PMID: 30269056; PubMed Central PMCID: PMCPMC6398328.

164.	Mayne SL, Moore KA, Powell-Wiley TM, Evenson KR, Block R, Kershaw KN. Longitudinal Associations of Neighborhood Crime and Perceived Safety With Blood Pressure: The Multi-Ethnic Study of Atherosclerosis (MESA). Am J Hypertens. 2018;31(9):1024-32. doi: 10.1093/ajh/hpy066. PubMed PMID: 29897398; PubMed Central PMCID: PMCPMC6077783.

165.	McClelland RL, Chung H, Detrano R, Post W, Kronmal RA. Distribution of coronary artery calcium by race, gender, and age: results from the Multi-Ethnic Study of Atherosclerosis (MESA). Circulation. 2006;113(1):30-7. Epub 20051219. doi: 10.1161/circulationaha.105.580696. PubMed PMID: 16365194.

166.	McClelland RL, Jorgensen NW, Post WS, Szklo M, Kronmal RA. Methods for estimation of disparities in medication use in an observational cohort study: results from the Multi-Ethnic Study of Atherosclerosis. Pharmacoepidemiol Drug Saf. 2013;22(5):533-41. Epub 20130204. doi: 10.1002/pds.3406. PubMed PMID: 23382107; PubMed Central PMCID: PMCPMC3652567.

167.	Medrano-Gracia P, Cowan BR, Ambale-Venkatesh B, Bluemke DA, Eng J, Finn JP, et al. Left ventricular shape variation in asymptomatic populations: the Multi-Ethnic Study of Atherosclerosis. J Cardiovasc Magn Reson. 2014;16(1):56. Epub 20140730. doi: 10.1186/s12968-014-0056-2. PubMed PMID: 25160814; PubMed Central PMCID: PMCPMC4145340.

168.	Mehta A, Pandey A, Ayers CR, Khera A, Sperling LS, Szklo MS, et al. Predictive Value of Coronary Artery Calcium Score Categories for Coronary Events Versus Strokes: Impact of Sex and Race: MESA and DHS. Circ Cardiovasc Imaging. 2020;13(8):e010153. Epub 20200818. doi: 10.1161/circimaging.119.010153. PubMed PMID: 32806939; PubMed Central PMCID: PMCPMC7440773.

169.	Merkin SS, Karlamangla A, Roux AD, Shrager S, Watson K, Seeman T. Race/ethnicity, neighborhood socioeconomic status and cardio-metabolic risk. SSM Popul Health. 2020;11(100634):100634. doi: 10.1016/j.ssmph.2020.100634. PubMed PMID: 32775593.

170.	Michos ED, Vaidya D, Gapstur SM, Schreiner PJ, Golden SH, Wong ND, et al. Sex hormones, sex hormone binding globulin, and abdominal aortic calcification in women and men in the multi-ethnic study of atherosclerosis (MESA). Atherosclerosis. 2008;200(2):432-8. Epub 20080208. doi: 10.1016/j.atherosclerosis.2007.12.032. PubMed PMID: 18262187; PubMed Central PMCID: PMCPMC2607033.

171.	Mongraw-Chaffin M, Golden SH, Allison MA, Ding J, Ouyang P, Schreiner PJ, et al. The Sex and Race Specific Relationship between Anthropometry and Body Fat Composition Determined from Computed Tomography: Evidence from the Multi-Ethnic Study of Atherosclerosis. PLoS One. 2015;10(10):e0139559. Epub 20151008. doi: 10.1371/journal.pone.0139559. PubMed PMID: 26448048; PubMed Central PMCID: PMCPMC4598154.

172.	Mongraw-Chaffin ML, Anderson CAM, Allison MA, Ouyang P, Szklo M, Vaidya D, et al. Association Between Sex Hormones and Adiposity: Qualitative Differences in Women and Men in the Multi-Ethnic Study of Atherosclerosis. Journal of Clinical Endocrinology & Metabolism. 2015;100(4):E596-E600. doi: 10.1210/jc.2014-2934. PubMed PMID: WOS:000353361500011.

173.	Moran A, Roux AVD, Jackson SA, Kramer H, Manolio TA, Shrager S, et al. Acculturation is associated with hypertension in a multiethnic sample. American Journal of Hypertension. 2007;20(4):354-63. doi: 10.1016/j.amjhyper.2006.09.025. PubMed PMID: WOS:000245686200003.

174.	Mujahid MS, Diez Roux AV, Cooper RC, Shea S, Williams DR. Neighborhood stressors and race/ethnic differences in hypertension prevalence (the Multi-Ethnic Study of Atherosclerosis). Am J Hypertens. 2011;24(2):187-93. Epub 20100916. doi: 10.1038/ajh.2010.200. PubMed PMID: 20847728; PubMed Central PMCID: PMCPMC3319083.

175.	Mujahid MS, Gao X, Tabb LP, Morris C, Lewis TT. Historical redlining and cardiovascular health: The Multi-Ethnic Study of Atherosclerosis. Proc Natl Acad Sci U S A. 2021;118(51). Epub 2021/12/15. doi: 10.1073/pnas.2110986118. PubMed PMID: 34903653; PubMed Central PMCID: PMCPMC8713797.

176.	Mujahid MS, Moore LV, Petito LC, Kershaw KN, Watson K, Diez Roux AV. Neighborhoods and racial/ethnic differences in ideal cardiovascular health (the Multi-Ethnic Study of Atherosclerosis). Health Place. 2017;44:61-9. Epub 2017/02/09. doi: 10.1016/j.healthplace.2017.01.005. PubMed PMID: 28167269; PubMed Central PMCID: PMCPMC5354104.

177.	Nasir K, Katz R, Takasu J, Shavelle DM, Detrano R, Lima JA, et al. Ethnic differences between extra-coronary measures on cardiac computed tomography: Multi-ethnic study of atherosclerosis (MESA. Atherosclerosis. 2008;198(1):104–14.

178.	Natori S, Lai S, Finn JP, Gomes AS, Hundley WG, Jerosch-Herold M, et al. Cardiovascular function in multi-ethnic study of atherosclerosis: normal values by age, sex, and ethnicity. AJR Am J Roentgenol. 2006;186(6 Suppl 2):S357-65. doi: 10.2214/ajr.04.1868. PubMed PMID: 16714609.

179.	Needham BL, Wang X, Carroll JE, Barber S, Sánchez BN, Seeman TE, et al. Sociodemographic correlates of change in leukocyte telomere length during mid- to late-life: The Multi-Ethnic Study of Atherosclerosis. Psychoneuroendocrinology. 2019;102:182-8. Epub 20181207. doi: 10.1016/j.psyneuen.2018.12.007. PubMed PMID: 30576944; PubMed Central PMCID: PMCPMC6445551.

180.	Nonterah EA, Crowther NJ, Klipstein-Grobusch K, Oduro AR, Kavousi M, Agongo G, et al. Racial and Ethnic Differences in the Association Between Classical Cardiovascular Risk Factors and Common Carotid Intima-Media Thickness: An Individual Participant Data Meta-Analysis. J Am Heart Assoc. 2022;11(15):e023704. Epub 2022/07/26. doi: 10.1161/JAHA.121.023704. PubMed PMID: 35876421; PubMed Central PMCID: PMCPMC9375511.

181.	Novak NL, Wang X, Clarke PJ, Hajat A, Needham BL, Sánchez BN, et al. Diurnal salivary cortisol and nativity/duration of residence in Latinos: The Multi-Ethnic Study of Atherosclerosis. Psychoneuroendocrinology. 2017;85:179-89. Epub 20170818. doi: 10.1016/j.psyneuen.2017.08.018. PubMed PMID: 28886460; PubMed Central PMCID: PMCPMC5623131.

182.	Oh SW, Wood AC, Hwang SS, Allison M. Racial and Ethnic Differences in the Association of Low-Carbohydrate Diet With Mortality in the Multi-Ethnic Study of Atherosclerosis. JAMA Netw Open. 2022;5(10):e2237552. Epub 20221003. doi: 10.1001/jamanetworkopen.2022.37552. PubMed PMID: 36264576; PubMed Central PMCID: PMCPMC9585425.

183.	Ohira T, Diez Roux AV, Polak JF, Homma S, Iso H, Wasserman BA. Associations of anger, anxiety, and depressive symptoms with carotid arterial wall thickness: the multi-ethnic study of atherosclerosis. Psychosom Med. 2012;74(5):517-25. Epub 20120417. doi: 10.1097/PSY.0b013e31824f6267. PubMed PMID: 22511725; PubMed Central PMCID: PMCPMC4966673.

184.	Ong KL, Ding J, McClelland RL, Cheung BM, Criqui MH, Barter PJ, et al. Relationship of pericardial fat with biomarkers of inflammation and hemostasis, and cardiovascular disease: the Multi-Ethnic Study of Atherosclerosis. Atherosclerosis. 2015;239(2):386-92. Epub 20150131. doi: 10.1016/j.atherosclerosis.2015.01.033. PubMed PMID: 25682037; PubMed Central PMCID: PMCPMC4361311.

185.	Ortiz MS, Myers HF, Dunkel Schetter C, Rodriguez CJ, Seeman TE. Psychosocial Predictors of Metabolic Syndrome among Latino Groups in the Multi-Ethnic Study of Atherosclerosis (MESA). PLoS One. 2015;10(4):e0124517. Epub 20150423. doi: 10.1371/journal.pone.0124517. PubMed PMID: 25906072; PubMed Central PMCID: PMCPMC4408074.

186.	Osibogun O, Ogunmoroti O, Mathews L, Okunrintemi V, Tibuakuu M, Michos ED. Greater Acculturation is Associated With Poorer Cardiovascular Health in the Multi-Ethnic Study of Atherosclerosis. J Am Heart Assoc. 2021;10(8):e019828. Epub 20210409. doi: 10.1161/jaha.120.019828. PubMed PMID: 33834848; PubMed Central PMCID: PMCPMC8174160.

187.	Osypuk TL, Diez Roux AV, Hadley C, Kandula NR. Are immigrant enclaves healthy places to live? The Multi-ethnic Study of Atherosclerosis. Soc Sci Med. 2009;69(1):110-20. Epub 20090508. doi: 10.1016/j.socscimed.2009.04.010. PubMed PMID: 19427731; PubMed Central PMCID: PMCPMC2750873.

188.	Oza-Frank R, Chan C, Liu K, Burke G, Kanaya AM. Incidence of type 2 diabetes by place of birth in the Multi-Ethnic Study of Atherosclerosis (MESA). J Immigr Minor Health. 2013;15(5):918-24. doi: 10.1007/s10903-012-9683-6. PubMed PMID: 22833256; PubMed Central PMCID: PMCPMC4039384.

189.	Palmas W, Ma S, Jacobs DR, Jr., Arnett D, Jackson S, Olson J, et al. Ethnicity and sex modify the association of serum c-reactive protein with microalbuminuria. Ethn Dis. 2008;18(3):324-9. PubMed PMID: 18785447; PubMed Central PMCID: PMCPMC4089959.

190.	Pan CW, Klein BE, Cotch MF, Shrager S, Klein R, Folsom A, et al. Racial variations in the prevalence of refractive errors in the United States: the multi-ethnic study of atherosclerosis. Am J Ophthalmol. 2013;155(6):1129-38 e1. doi: 10.1016/j.ajo.2013.01.009. PubMed PMID: 23453694.

191.	Pandey A, Omar W, Ayers C, LaMonte M, Klein L, Allen NB, et al. Sex and Race Differences in Lifetime Risk of Heart Failure With Preserved Ejection Fraction and Heart Failure With Reduced Ejection Fraction. Circulation. 2018;137(17):1814-23. Epub 20180119. doi: 10.1161/circulationaha.117.031622. PubMed PMID: 29352072; PubMed Central PMCID: PMCPMC6417883.

192.	Paramsothy P, Knopp R, Bertoni AG, Tsai MY, Rue T, Heckbert SR. Combined hyperlipidemia in relation to race/ethnicity, obesity, and insulin resistance in the Multi-Ethnic Study of Atherosclerosis. Metabolism. 2009;58(2):212-9. doi: 10.1016/j.metabol.2008.09.016. PubMed PMID: 19154954; PubMed Central PMCID: PMCPMC2677914.

193.	Park SK, Adar SD, O'Neill MS, Auchincloss AH, Szpiro A, Bertoni AG, et al. Long-term exposure to air pollution and type 2 diabetes mellitus in a multiethnic cohort. Am J Epidemiol. 2015;181(5):327-36. Epub 20150217. doi: 10.1093/aje/kwu280. PubMed PMID: 25693777; PubMed Central PMCID: PMCPMC4339386.

194.	Patel J, Mehta A, Rifai MA, Blaha MJ, Nasir K, McEvoy JW, et al. Hypertension guidelines and coronary artery calcification among South Asians: Results from MASALA and MESA. Am J Prev Cardiol. 2021;6(100158):100158. doi: 10.1016/j.ajpc.2021.100158. PubMed PMID: 34327495.

195.	Patel RJS, Ding J, Marvel FA, Shan R, Plante TB, Blaha MJ, et al. Associations of Demographic, Socioeconomic, and Cognitive Characteristics With Mobile Health Access: MESA (Multi-Ethnic Study of Atherosclerosis). J Am Heart Assoc. 2022;11(17):e024885. Epub 20220903. doi: 10.1161/jaha.121.024885. PubMed PMID: 36056720; PubMed Central PMCID: PMCPMC9496404.

196.	Patton KK, Heckbert SR, Alonso A, Bahrami H, Lima JAC, Burke G, et al. N-terminal pro-B-type natriuretic peptide as a predictor of incident atrial fibrillation in the Multi-Ethnic Study of Atherosclerosis: the effects of age, sex and ethnicity. Heart. 2013;99(24):1832-6. doi: 10.1136/heartjnl-2013-304724. PubMed PMID: WOS:000327249800008.

197.	Peplinski B, McClelland R, Szklo M. Associations between socioeconomic status markers and depressive symptoms by race and gender: results from the Multi-Ethnic Study of Atherosclerosis (MESA. Ann Epidemiol. 2018;28(8):535–42.

198.	Peralta CA, Li YM, Wassel C, Choudhry S, Palmas W, Seldin MF, et al. Differences in Albuminuria Between Hispanics and Whites: An Evaluation by Genetic Ancestry and Country of Origin The Multi-Ethnic Study of Atherosclerosis. Circulation-Cardiovascular Genetics. 2010;3(3):240-7. doi: 10.1161/Circgenetics.109.914499. PubMed PMID: WOS:000278799900005.

199.	Pike MM, Larson NB, Wassel CL, Cohoon KP, Tsai MY, Pankow JS, et al. ABO blood group is associated with peripheral arterial disease in African Americans: The Multi-Ethnic Study of Atherosclerosis (MESA. Thromb Res. 2017;153:1–6.

200.	Post WS, Watson KE, Hansen S, Folsom AR, Szklo M, Shea S, et al. Racial and Ethnic Differences in All-Cause and Cardiovascular Disease Mortality: The MESA Study. Circulation. 2022;146(3):229-39. Epub 20220718. doi: 10.1161/circulationaha.122.059174. PubMed PMID: 35861763; PubMed Central PMCID: PMCPMC9937428.

201.	Powell R, Davidson D, Divers J, Manichaikul A, Carr JJ, Detrano R, et al. Genetic ancestry and the relationship of cigarette smoking to lung function and per cent emphysema in four race/ethnic groups: a cross-sectional study. Thorax. 2013;68(7):634-42. doi: 10.1136/thoraxjnl-2012-202116. PubMed PMID: 23585509.

202.	Ranjit N, Diez-Roux AV, Shea S, Cushman M, Ni H, Seeman T. Socioeconomic position, race/ethnicity, and inflammation in the multi-ethnic study of atherosclerosis. Circulation. 2007;116(21):2383-90. doi: 10.1161/circulationaha.107.706226. PubMed PMID: 18025402.

203.	Rasmussen-Torvik LJ, Wassel CL, Ding J, Carr J, Cushman M, Jenny N, et al. Associations of body mass index and insulin resistance with leptin, adiponectin, and the leptin-to-adiponectin ratio across ethnic groups: the Multi-Ethnic Study of Atherosclerosis (MESA). Ann Epidemiol. 2012;22(10):705-9. Epub 20120825. doi: 10.1016/j.annepidem.2012.07.011. PubMed PMID: 22929534; PubMed Central PMCID: PMCPMC3459265.

204.	Reddy VK, Gapstur SM, Prineas R, Colangelo LA, Ouyang P, Kadish AH. Ethnic differences in ST height in the multiethnic study of atherosclerosis. Ann Noninvasive Electrocardiol. 2008;13(4):341-51. doi: 10.1111/j.1542-474X.2008.00252.x. PubMed PMID: 18973491; PubMed Central PMCID: PMCPMC2637518.

205.	Reis Geovanini G, Wang R, Weng J, Jenny NS, Shea S, Allison M, et al. Association between Obstructive Sleep Apnea and Cardiovascular Risk Factors: Variation by Age, Sex and Race. The Multi-Ethnic Study of Atherosclerosis. Ann Am Thorac Soc. 2018;15(8):970–7.

206.	Remigio-Baker RA, Allison MA, Forbang NI, Loomba R, Anderson CAM, Budoff M, et al. Race/ethnic and sex disparities in the non-alcoholic fatty liver disease-abdominal aortic calcification association: The Multi-Ethnic Study of Atherosclerosis. Atherosclerosis. 2017;258:89-96. Epub 20161117. doi: 10.1016/j.atherosclerosis.2016.11.021. PubMed PMID: 28235711; PubMed Central PMCID: PMCPMC5502083.

207.	Remigio-Baker RA, Allison MA, Schreiner PJ, Carnethon MR, Nettleton JA, Mujahid MS, et al. Sex and race/ethnic disparities in the cross-sectional association between depressive symptoms and muscle mass: the Multi-ethnic Study of Atherosclerosis. BMC Psychiatry. 2015;15(1):221. doi: 10.1186/s12888-015-0604-9. PubMed PMID: 26384322.

208.	Remigio-Baker RA, Allison MA, Schreiner PJ, Szklo M, Crum RM, Leoutsakos JM, et al. Difference by sex but not by race/ethnicity in the visceral adipose tissue-depressive symptoms association: the Multi-Ethnic Study of Atherosclerosis. Psychoneuroendocrinology. 2014;47:78-87. Epub 20140513. doi: 10.1016/j.psyneuen.2014.05.004. PubMed PMID: 25001957; PubMed Central PMCID: PMCPMC4134940.

209.	Rifkin DE, Khaki AR, Jenny NS, McClelland RL, Budoff M, Watson K, et al. Association of renin and aldosterone with ethnicity and blood pressure: the Multi-Ethnic Study of Atherosclerosis. Am J Hypertens. 2014;27(6):801-10. Epub 20140116. doi: 10.1093/ajh/hpt276. PubMed PMID: 24436325; PubMed Central PMCID: PMCPMC4017931.

210.	Robinson-Cohen C, Hoofnagle AN, Ix JH, Sachs MC, Tracy RP, Siscovick DS, et al. Racial differences in the association of serum 25-hydroxyvitamin D concentration with coronary heart disease events. Jama. 2013;310(2):179-88. doi: 10.1001/jama.2013.7228. PubMed PMID: 23839752; PubMed Central PMCID: PMCPMC4150653.

211.	Robinson-Cohen C, Shlipak M, Sarnak M, Katz R, Peralta C, Young B, et al. Impact of Race on the Association of Mineral Metabolism With Heart Failure: the Multi-Ethnic Study of Atherosclerosis. J Clin Endocrinol Metab. 2020;105(4):e1144-51. doi: 10.1210/clinem/dgz218. PubMed PMID: 31760429; PubMed Central PMCID: PMCPMC7064305.

212.	Robinson-Cohen C, Zelnick LR, Hoofnagle AN, Lutsey PL, Burke G, Michos ED, et al. Associations of Vitamin D-Binding Globulin and Bioavailable Vitamin D Concentrations With Coronary Heart Disease Events: The Multi-Ethnic Study of Atherosclerosis (MESA. J Clin Endocrinol Metab. 2017;102(8):3075–84.

213.	Rodriguez CJ, Diez-Roux AV, Moran A, Jin Z, Kronmal RA, Lima J, et al. Left ventricular mass and ventricular remodeling among Hispanic subgroups compared with non-Hispanic blacks and whites: MESA (Multi-ethnic Study of Atherosclerosis. J Am Coll Cardiol. 2010;55(3):234–42.

214.	Rodriguez LA, Bradshaw PT, Shiboski SC, Fernandez A, Vittinghoff E, Herrington D, et al. Examining if the relationship between BMI and incident type 2 diabetes among middle-older aged adults varies by race/ethnicity: evidence from the Multi-Ethnic Study of Atherosclerosis (MESA). Diabet Med. 2021;38(5):e14377. Epub 20200922. doi: 10.1111/dme.14377. PubMed PMID: 32750175; PubMed Central PMCID: PMCPMC7858695.

215.	Rodriguez LA, Jin Y, Talegawkar SA, Oliveira Otto MC, Kandula NR, Herrinton DM, et al. Differences in Diet Quality Among Multiple US Racial/Ethnic Groups From the Mediators of Atherosclerosis in South Asians Living in America (MASALA) Study and the Multi-Ethnic Study of Atherosclerosis (MESA. J Nutr. 2020;150(6):1509–15.

216.	Sachs BC, Steenland K, Zhao L, Hughes TM, Weintraub S, Dodge HH, et al. Expanded Demographic Norms for Version 3 of the Alzheimer Disease Centers' Neuropsychological Test Battery in the Uniform Data Set. Alzheimer Dis Assoc Disord. 2020;34(3):191-7. Epub 2020/06/03. doi: 10.1097/WAD.0000000000000388. PubMed PMID: 32483017; PubMed Central PMCID: PMCPMC7842186.

217.	Sanchez DR, Diez Roux AV, Michos ED, Blumenthal RS, Schreiner PJ, Burke GL, et al. Comparison of the racial/ethnic prevalence of regular aspirin use for the primary prevention of coronary heart disease from the multi-ethnic study of atherosclerosis. Am J Cardiol. 2011;107(1):41-6. doi: 10.1016/j.amjcard.2010.08.041. PubMed PMID: 21146684; PubMed Central PMCID: PMCPMC3021117.

218.	Sands SA, Alex RM, Mann D, Vena D, Terrill PI, Gell LK, et al. Pathophysiology Underlying Demographic and Obesity Determinants of Sleep Apnea Severity. Ann Am Thorac Soc. 2022.

219.	Segar MW, Jaeger BC, Patel KV, Nambi V, Ndumele CE, Correa A, et al. Development and Validation of Machine Learning-Based Race-Specific Models to Predict 10-Year Risk of Heart Failure A Multicohort Analysis. Circulation. 2021;143(24):2370-83. doi: 10.1161/Circulationaha.120.053134. PubMed PMID: WOS:000661620000010.

220.	Shah AD, Kandula NR, Lin F, Allison MA, Carr J, Herrington D, et al. Less favorable body composition and adipokines in South Asians compared with other US ethnic groups: results from the MASALA and MESA studies. Int J Obes (Lond). 2016;40(4):639-45. Epub 20151208. doi: 10.1038/ijo.2015.219. PubMed PMID: 26499444; PubMed Central PMCID: PMCPMC4821815.

221.	Shea MK, Booth SL, Nettleton JA, Burke GL, Chen H, Kritchevsky SB. Circulating phylloquinone concentrations of adults in the United States differ according to race and ethnicity. J Nutr. 2012;142(6):1060-6. doi: 10.3945/jn.111.154278. PubMed PMID: 22496402.

222.	Shea S, Lima J, Diez-Roux A, Jorgensen NW, McClelland RL. Socioeconomic Status and Poor Health Outcome at 10 Years of Follow-Up in the Multi-Ethnic Study of Atherosclerosis. PLoS One. 2016;11(11):e0165651. Epub 20161122. doi: 10.1371/journal.pone.0165651. PubMed PMID: 27875557; PubMed Central PMCID: PMCPMC5119729.

223.	Shivpuri S, Gallo LC, Crouse JR, Allison MA. The association between chronic stress type and C-reactive protein in the multi-ethnic study of atherosclerosis: does gender make a difference? J Behav Med. 2012;35(1):74–85.

224.	Silverman MG, Patel B, Blankstein R, Lima JA, Blumenthal RS, Nasir K, et al. Impact of Race, Ethnicity, and Multimodality Biomarkers on the Incidence of New-Onset Heart Failure With Preserved Ejection Fraction (from the Multi-Ethnic Study of Atherosclerosis). Am J Cardiol. 2016;117(9):1474-81. Epub 20160217. doi: 10.1016/j.amjcard.2016.02.017. PubMed PMID: 27001449; PubMed Central PMCID: PMCPMC4999314.

225.	Singleton MJ, German CA, Carnethon M, Soliman EZ, Bertoni AG, Yeboah J. Race, Body Mass Index, and the Risk of Atrial Fibrillation: The Multi-Ethnic Study of Atherosclerosis. J Am Heart Assoc. 2021;10(1):e018592. Epub 20201231. doi: 10.1161/jaha.120.018592. PubMed PMID: 33382342; PubMed Central PMCID: PMCPMC7955459.

226.	Song L, Smith GS, Adar SD, Post WS, Guallar E, Navas-Acien A, et al. Ambient air pollution as a mediator in the pathway linking race/ethnicity to blood pressure elevation: The multi-ethnic study of atherosclerosis (MESA). Environ Res. 2020;180(108776):108776. doi: 10.1016/j.envres.2019.108776. PubMed PMID: 31639655.

227.	Spalt EW, Curl CL, Allen RW, Cohen M, Williams K, Hirsch JA, et al. Factors influencing time-location patterns and their impact on estimates of exposure: the Multi-Ethnic Study of Atherosclerosis and Air Pollution (MESA Air. J Expo Sci Environ Epidemiol. 2016;26(4):341–8.

228.	Steffen BT, Duprez D, Bertoni AG, Guan W, Tsai MY. Lp(a) [Lipoprotein(a)]-Related Risk of Heart Failure Is Evident in Whites by Not in Other Racial/Ethnic Groups. Arterioscler Thromb Vasc Biol. 2018;38(10):2498–504.

229.	Steffen BT, Guan W, Ding J, Nomura SO, Weir NL, Tsai MY. Plasma omega-3 and saturated fatty acids are differentially related to pericardial adipose tissue volume across race/ethnicity: the Multi-ethnic Study of Atherosclerosis. Eur J Clin Nutr. 2021;75(8):1237-44. Epub 20210104. doi: 10.1038/s41430-020-00833-x. PubMed PMID: 33398103; PubMed Central PMCID: PMCPMC8254815.

230.	Steffen BT, Steffen LM, Tracy R, Siscovick D, Jacobs D, Liu K, et al. Ethnicity, plasma phospholipid fatty acid composition and inflammatory/endothelial activation biomarkers in the Multi-Ethnic Study of Atherosclerosis (MESA). Eur J Clin Nutr. 2012;66(5):600-5. Epub 20120104. doi: 10.1038/ejcn.2011.215. PubMed PMID: 22215136; PubMed Central PMCID: PMCPMC4096912.

231.	Steffen BT, Thanassoulis G, Duprez D, Stein JH, Karger AB, Tattersall MC, et al. Race-Based Differences in Lipoprotein(a)-Associated Risk of Carotid Atherosclerosis. Arterioscler Thromb Vasc Biol. 2019;39(3):523-9. doi: 10.1161/atvbaha.118.312267. PubMed PMID: 30727753; PubMed Central PMCID: PMCPMC6393189.

232.	Stern R, Tattersall MC, Gepner AD, Korcarz CE, Kaufman J, Colangelo LA, et al. Sex differences in predictors of longitudinal changes in carotid artery stiffness: the Multi-Ethnic Study of Atherosclerosis. Arterioscler Thromb Vasc Biol. 2015;35(2):478-84. Epub 20141204. doi: 10.1161/atvbaha.114.304870. PubMed PMID: 25477347; PubMed Central PMCID: PMCPMC4304990.

233.	Subramanyam MA, Diez-Roux AV, Pilsner JR, Villamor E, Donohue KM, Liu Y, et al. Social factors and leukocyte DNA methylation of repetitive sequences: the multi-ethnic study of atherosclerosis. PLoS One. 2013;8(1):e54018. Epub 20130108. doi: 10.1371/journal.pone.0054018. PubMed PMID: 23320117; PubMed Central PMCID: PMCPMC3539988.

234.	Tabb LP, McClure LA, Ortiz A, Melly S, Jones MR, Kershaw KN, et al. Assessing the spatial heterogeneity in black-white differences in optimal cardiovascular health and the impact of individual- and neighborhood-level risk factors: The Multi-Ethnic Study of Atherosclerosis (MESA). Spat Spatiotemporal Epidemiol. 2020;33(100332):100332. doi: 10.1016/j.sste.2020.100332. PubMed PMID: 32370943.

235.	Takasu J, Katz R, Nasir K, Carr JJ, Wong N, Detrano R, et al. Relationships of thoracic aortic wall calcification to cardiovascular risk factors: the Multi-Ethnic Study of Atherosclerosis (MESA). Am Heart J. 2008;155(4):765-71. Epub 20080221. doi: 10.1016/j.ahj.2007.11.019. PubMed PMID: 18371491; PubMed Central PMCID: PMCPMC2323597.

236.	Tandri H, Daya SK, Nasir K, Bomma C, Lima JA, Calkins H, et al. Normal reference values for the adult right ventricle by magnetic resonance imaging. Am J Cardiol. 2006;98(12):1660-4. Epub 20061027. doi: 10.1016/j.amjcard.2006.07.049. PubMed PMID: 17145230.

237.	Tanner RM, Woodward M, Peralta C, Warnock DG, Gutiérrez O, Shimbo D, et al. Validation of an Albuminuria Self-assessment Tool in the Multi-Ethnic Study of Atherosclerosis. Ethn Dis. 2015;25(4):427-34. Epub 20151105. doi: 10.18865/ed.25.4.427. PubMed PMID: 26676090; PubMed Central PMCID: PMCPMC4671433.

238.	Tattersall MC, Gassett A, Korcarz CE, Gepner AD, Kaufman JD, Liu KJ, et al. Predictors of carotid thickness and plaque progression during a decade: the Multi-Ethnic Study of Atherosclerosis. Stroke. 2014;45(11):3257-62. Epub 20140911. doi: 10.1161/strokeaha.114.005669. PubMed PMID: 25213342; PubMed Central PMCID: PMCPMC4213289.

239.	Thomas IC, Shiau B, Denenberg JO, McClelland RL, Greenland P, de Boer IH, et al. Association of cardiovascular disease risk factors with coronary artery calcium volume versus density. Heart. 2018;104(2):135-43. Epub 20170816. doi: 10.1136/heartjnl-2017-311536. PubMed PMID: 28814488; PubMed Central PMCID: PMCPMC6004322.

240.	Tota-Maharaj R, Blaha MJ, Zeb I, Katz R, Blankstein R, Blumenthal RS, et al. Ethnic and sex differences in fatty liver on cardiac computed tomography: the multi-ethnic study of atherosclerosis. Mayo Clin Proc. 2014;89(4):493-503. Epub 20140307. doi: 10.1016/j.mayocp.2013.12.015. PubMed PMID: 24613289; PubMed Central PMCID: PMCPMC4410019.

241.	Turkbey EB, Jain A, Johnson C, Redheuil A, Arai AE, Gomes AS, et al. Determinants and normal values of ascending aortic diameter by age, gender, and race/ethnicity in the Multi-Ethnic Study of Atherosclerosis (MESA). J Magn Reson Imaging. 2014;39(2):360-8. Epub 20130516. doi: 10.1002/jmri.24183. PubMed PMID: 23681649; PubMed Central PMCID: PMCPMC3766427.

242.	Turkbey EB, Nacif MS, Guo M, McClelland RL, Teixeira PB, Bild DE, et al. Prevalence and Correlates of Myocardial Scar in a US Cohort. Jama. 2015;314(18):1945-54. doi: 10.1001/jama.2015.14849. PubMed PMID: 26547466; PubMed Central PMCID: PMCPMC4774246.

243.	Vaidya D, Heckbert SR, Wasserman BA, Ouyang P. Sex-specific association of age with carotid artery distensibility: multi-ethnic study of atherosclerosis. J Womens Health (Larchmt). 2012;21(5):516-20. Epub 20120306. doi: 10.1089/jwh.2011.3220. PubMed PMID: 22393881; PubMed Central PMCID: PMCPMC3353825.

244.	Vargas JD, Manichaikul A, Wang XQ, Rich SS, Rotter JI, Post WS, et al. Common genetic variants and subclinical atherosclerosis: The Multi-Ethnic Study of Atherosclerosis (MESA). Atherosclerosis. 2016;245:230-6. Epub 20151208. doi: 10.1016/j.atherosclerosis.2015.11.034. PubMed PMID: 26789557; PubMed Central PMCID: PMCPMC4738145.

245.	Wagenknecht LE, Divers J, Bertoni AG, Langefeld CD, Carr JJ, Bowden DW, et al. Correlates of coronary artery calcified plaque in blacks and whites with type 2 diabetes. Ann Epidemiol. 2011;21(1):34-41. doi: 10.1016/j.annepidem.2010.10.007. PubMed PMID: 21130367; PubMed Central PMCID: PMCPMC3026318.

246.	Weatherspoon DJ, Borrell LN, Johnson CW, Mujahid MS, Neighbors HW, Adar SD. Racial and Ethnic Differences in Self-Reported Periodontal Disease in the Multi-Ethnic Study of Atherosclerosis (MESA). Oral Health Prev Dent. 2016;14(3):249-57. doi: 10.3290/j.ohpd.a35614. PubMed PMID: 26870845; PubMed Central PMCID: PMCPMC4970861.

247.	Weze KO, Obisesan OH, Dardari ZA, Cainzos-Achirica M, Dzaye O, Graham G, et al. The Interplay of Race/Ethnicity and Obesity on the Incidence of Venous Thromboembolism. Am J Prev Med. 2022;63(1):e11-e20. doi: 10.1016/j.amepre.2021.12.023. PubMed PMID: 35260291.

248.	Whitaker KM, Everson-Rose SA, Pankow JS, Rodriguez CJ, Lewis TT, Kershaw KN, et al. Experiences of Discrimination and Incident Type 2 Diabetes Mellitus: The Multi-Ethnic Study of Atherosclerosis (MESA). Am J Epidemiol. 2017;186(4):445-55. doi: 10.1093/aje/kwx047. PubMed PMID: 28407040; PubMed Central PMCID: PMCPMC5860562.

249.	Win TT, Vasconcellos HD, Chamera E, Hong SY, Ambale Venkatesh B, Young P, et al. References Values for Left Atrial Volumes, Emptying Fractions, Strains, and Strain Rates and Their Determinants by Age, Gender, and Ethnicity: The Multiethnic Study of Atherosclerosis (MESA. Acad Radiol. 2021;28(3):356–63.

250.	Winston GJ, Barr RG, Carrasquillo O, Bertoni AG, Shea S. Sex and Racial/Ethnic Differences in Cardiovascular Disease Risk Factor Treatment and Control Among Individuals With Diabetes in the Multi-Ethnic Study of Atherosclerosis (MESA. Diabetes Care. 2009;32(8):1467–9.

251.	Yadlowsky S, Hayward RA, Sussman JB, McClelland RL, Min YI, Basu S. Clinical Implications of Revised Pooled Cohort Equations for Estimating Atherosclerotic Cardiovascular Disease Risk. Annals of Internal Medicine. 2018;169(1):20-+. doi: 10.7326/M17-3011. PubMed PMID: WOS:000436931900004.

252.	Yamagishi K, Ohira T, Nakano H, Bielinski SJ, Sakurai S, Imano H, et al. Cross-cultural comparison of the sleep-disordered breathing prevalence among Americans and Japanese. European Respiratory Journal. 2010;36(2):379-84. doi: 10.1183/09031936.00118609. PubMed PMID: WOS:000281601800023.

253.	Youssef G, Guo M, McClelland RL, Shavelle DM, Nasir K, Rivera J, et al. Risk Factors for the Development and Progression of Thoracic Aorta Calcification: The Multi-Ethnic Study of Atherosclerosis. Acad Radiol. 2015;22(12):1536-45. Epub 20150926. doi: 10.1016/j.acra.2015.08.017. PubMed PMID: 26403646; PubMed Central PMCID: PMCPMC4636912.

254.	Zemrak F, Ambale-Venkatesh B, Captur G, Chrispin J, Chamera E, Habibi M, et al. Left Atrial Structure in Relationship to Age, Sex, Ethnicity, and Cardiovascular Risk Factors: MESA (Multi-Ethnic Study of Atherosclerosis). Circ Cardiovasc Imaging. 2017;10(2):116 005379. doi: 10.1161/CIRCIMAGING.116.005379. PubMed PMID: 28196797.

255.	Zhao W, Ware EB, He Z, Kardia SLR, Faul JD, Smith JA. Interaction between Social/Psychosocial Factors and Genetic Variants on Body Mass Index: A Gene-Environment Interaction Analysis in a Longitudinal Setting. Int J Environ Res Public Health. 2017;14(10):1153. doi: 10.3390/ijerph14101153. PubMed PMID: 28961216.

256.	Zhao Y, Malik S, Budoff MJ, Correa A, Ashley KE, Selvin E, et al. Identification and Predictors for Cardiovascular Disease Risk Equivalents among Adults With Diabetes Mellitus. Diabetes Care. 2021. Epub 20210811. doi: 10.2337/dc21-0431. PubMed PMID: 34380703.

257.	Zhao YY, Javaheri S, Wang R, Guo N, Koo BB, Stein JH, et al. Associations Between Sleep Apnea and Subclinical Carotid Atherosclerosis: The Multi-Ethnic Study of Atherosclerosis. Stroke. 2019;50(12):3340-6. Epub 20191015. doi: 10.1161/strokeaha.118.022184. PubMed PMID: 31610764; PubMed Central PMCID: PMCPMC6878193.

258.	Zipursky RT, Press MC, Srikanthan P, Gornbein J, McClelland R, Watson K, et al. Relation of Stress Hormones (Urinary Catecholamines/Cortisol) to Coronary Artery Calcium in Men Versus Women (from the Multi-Ethnic Study of Atherosclerosis [MESA]). Am J Cardiol. 2017;119(12):1963-71. Epub 20170329. doi: 10.1016/j.amjcard.2017.03.025. PubMed PMID: 28456316.

259.	Besser LM, Hirsch J, Galvin JE, Renne J, Park J, Evenson KR, et al. Associations between neighborhood park space and cognition in older adults vary by US location: The Multi-Ethnic Study of Atherosclerosis. Health Place. 2020;66(102459):102459. doi: 10.1016/j.healthplace.2020.102459. PubMed PMID: 33045671.

260.	Besser LM, Chang LC, Hirsch JA, Rodriguez DA, Renne J, Rapp SR, et al. Longitudinal Associations between the Neighborhood Built Environment and Cognition in US Older Adults: The Multi-Ethnic Study of Atherosclerosis. Int J Environ Res Public Health. 2021;18(15). Epub 2021/08/08. doi: 10.3390/ijerph18157973. PubMed PMID: 34360264; PubMed Central PMCID: PMCPMC8345405.

261.	Sisco S, Gross AL, Shih RA, Sachs BC, Glymour MM, Bangen KJ, et al. The role of early-life educational quality and literacy in explaining racial disparities in cognition in late life. J Gerontol B Psychol Sci Soc Sci. 2015;70(4):557-67. Epub 2014/03/04. doi: 10.1093/geronb/gbt133. PubMed PMID: 24584038; PubMed Central PMCID: PMCPMC4462668.

262.	Glymour MM, Manly JJ. Lifecourse Social Conditions and Racial and Ethnic Patterns of Cognitive Aging. Neuropsychology Review. 2008;18(3):223-54. doi: 10.1007/s11065-008-9064-z. PubMed PMID: WOS:000260068800005.

263.	Aiello AE, Diez-Roux A, Noone AM, Ranjit N, Cushman M, Tsai MY, et al. Socioeconomic and psychosocial gradients in cardiovascular pathogen burden and immune response: the multi-ethnic study of atherosclerosis. Brain Behav Immun. 2009;23(5):663-71. Epub 20081225. doi: 10.1016/j.bbi.2008.12.006. PubMed PMID: 19150399; PubMed Central PMCID: PMCPMC2694852.

264.	Anderson MD, Merkin SS, Everson-Rose SA, Widome R, Seeman T, Magnani JW, et al. Health Literacy Within a Diverse Community-Based Cohort: The Multi-Ethnic Study of Atherosclerosis. J Immigr Minor Health. 2021;23(4):659-67. Epub 20201118. doi: 10.1007/s10903-020-01123-1. PubMed PMID: 33206278; PubMed Central PMCID: PMCPMC8128937.

265.	Auchincloss AH, Diez Roux AV, Brown DG, O'Meara ES, Raghunathan TE. Association of insulin resistance with distance to wealthy areas: the multi-ethnic study of atherosclerosis. Am J Epidemiol. 2007;165(4):389-97. Epub 20061205. doi: 10.1093/aje/kwk028. PubMed PMID: 17148499.

266.	Brenner AB, Diez Roux AV, Barrientos-Gutierrez T, Borrell LN. Associations of Alcohol Availability and Neighborhood Socioeconomic Characteristics With Drinking: Cross-Sectional Results From the Multi-Ethnic Study of Atherosclerosis (MESA). Subst Use Misuse. 2015;50(12):1606-17. Epub 20151118. doi: 10.3109/10826084.2015.1027927. PubMed PMID: 26579610; PubMed Central PMCID: PMCPMC4802501.

267.	Brown KM, Diez-Roux AV, Smith JA, Needham BL, Mukherjee B, Ware EB, et al. Expression of socially sensitive genes: The multi-ethnic study of atherosclerosis. PLoS One. 2019;14(4):e0214061. Epub 20190411. doi: 10.1371/journal.pone.0214061. PubMed PMID: 30973896; PubMed Central PMCID: PMCPMC6459532.

268.	Brown KM, Diez-Roux AV, Smith JA, Needham BL, Mukherjee B, Ware EB, et al. Social regulation of inflammation related gene expression in the multi-ethnic study of atherosclerosis. Psychoneuroendocrinology. 2020;117:104654. Epub 20200507. doi: 10.1016/j.psyneuen.2020.104654. PubMed PMID: 32387875; PubMed Central PMCID: PMCPMC7685527.

269.	Christine PJ, Young R, Adar SD, Bertoni AG, Heisler M, Carnethon MR, et al. Individual- and Area-Level SES in Diabetes Risk Prediction: The Multi-Ethnic Study of Atherosclerosis. Am J Prev Med. 2017;53(2):201-9. doi: 10.1016/j.amepre.2017.04.019. PubMed PMID: 28625713; PubMed Central PMCID: PMCPMC5584566.

270.	Crawford ND, Moore K, Christine PJ, Barrientos-Gutierrez T, Seeman T, Diez Roux AV. Examining the Role of Neighborhood-Level Foreclosure in Smoking and Alcohol Use Among Older Adults in the Multi-Ethnic Study of Atherosclerosis. Am J Epidemiol. 2018;187(9):1863-70. doi: 10.1093/aje/kwy075. PubMed PMID: 29961880; PubMed Central PMCID: PMCPMC6118061.

271.	Curl CL, Beresford SA, Hajat A, Kaufman JD, Moore K, Nettleton JA, et al. Associations of organic produce consumption with socioeconomic status and the local food environment: Multi-Ethnic Study of Atherosclerosis (MESA). PLoS One. 2013;8(7):e69778. Epub 20130731. doi: 10.1371/journal.pone.0069778. PubMed PMID: 23936098; PubMed Central PMCID: PMCPMC3729963.

272.	De Moraes ACF, Carvalho HB, McClelland RL, Diez-Roux AV, Szklo M. Sex and ethnicity modify the associations between individual and contextual socioeconomic indicators and ideal cardiovascular health: MESA study. Journal of Public Health. 2018:fdy145-fdy. doi: 10.1093/pubmed/fdy145.

273.	Delaney JA, Biggs ML, Kronmal RA, Psaty BM. Demographic, medical, and behavioral characteristics associated with over the counter non-steroidal anti-inflammatory drug use in a population-based cohort: results from the Multi-Ethnic Study of Atherosclerosis. Pharmacoepidemiol Drug Saf. 2011;20(1):83-9. Epub 20101111. doi: 10.1002/pds.2065. PubMed PMID: 21182156; PubMed Central PMCID: PMCPMC3014611.

274.	Diez Roux AV, Auchincloss AH, Franklin TG, Raghunathan T, Barr RG, Kaufman J, et al. Long-term exposure to ambient particulate matter and prevalence of subclinical atherosclerosis in the Multi-Ethnic Study of Atherosclerosis. Am J Epidemiol. 2008;167(6):667-75. Epub 20080127. doi: 10.1093/aje/kwm359. PubMed PMID: 18227099.

275.	Diez Roux AV, Detrano R, Jackson S, Jacobs DR, Jr., Schreiner PJ, Shea S, et al. Acculturation and socioeconomic position as predictors of coronary calcification in a multiethnic sample. Circulation. 2005;112(11):1557-65. Epub 20050906. doi: 10.1161/circulationaha.104.530147. PubMed PMID: 16144996.

276.	Diez Roux AV, Evenson KR, McGinn AP, Brown DG, Moore L, Brines S, et al. Availability of recreational resources and physical activity in adults. American Journal of Public Health. 2007;97(3):493-9. Epub 2007/02/03. doi: 10.2105/ajph.2006.087734. PubMed PMID: 17267710; PubMed Central PMCID: PMCPMC1805019.

277.	Evenson KR, Block R, Diez Roux AV, McGinn AP, Wen F, Rodríguez DA. Associations of adult physical activity with perceived safety and police-recorded crime: the Multi-ethnic Study of Atherosclerosis. Int J Behav Nutr Phys Act. 2012;9:146. Epub 20121217. doi: 10.1186/1479-5868-9-146. PubMed PMID: 23245527; PubMed Central PMCID: PMCPMC3543301.

278.	Fitzpatrick AL, Rapp SR, Luchsinger J, Hill-Briggs F, Alonso A, Gottesman R, et al. Sociodemographic Correlates of Cognition in the Multi-Ethnic Study of Atherosclerosis (MESA). The American Journal of Geriatric Psychiatry. 2015;23(7):684-97. doi: http://dx.doi.org/10.1016/j.jagp.2015.01.003.

279.	Fujishiro K, Diez Roux AV, Landsbergis P, Baron S, Barr RG, Kaufman JD, et al. Associations of occupation, job control and job demands with intima-media thickness: the Multi-Ethnic Study of Atherosclerosis (MESA). Occup Environ Med. 2011;68(5):319-26. Epub 20101008. doi: 10.1136/oem.2010.055582. PubMed PMID: 20935285; PubMed Central PMCID: PMCPMC3073024.

280.	Fujishiro K, Diez-Roux AV, Landsbergis PA, Jenny NS, Seeman T. Current employment status, occupational category, occupational hazard exposure and job stress in relation to telomere length: the Multiethnic Study of Atherosclerosis (MESA). Occup Environ Med. 2013;70(8):552-60. doi: 10.1136/oemed-2012-101296. PubMed PMID: 23686115.

281.	Fujishiro K, Hajat A, Landsbergis PA, Meyer JD, Schreiner PJ, Kaufman JD. Explaining racial/ethnic differences in all-cause mortality in the Multi-Ethnic Study of Atherosclerosis (MESA): Substantive complexity and hazardous working conditions as mediating factors. SSM Popul Health. 2017;3:497-505. Epub 20170514. doi: 10.1016/j.ssmph.2017.05.010. PubMed PMID: 29349240; PubMed Central PMCID: PMCPMC5769063.

282.	Fujishiro K, Stukovsky KD, Roux AD, Landsbergis P, Burchfiel C. Occupational gradients in smoking behavior and exposure to workplace environmental tobacco smoke: the multi-ethnic study of atherosclerosis. J Occup Environ Med. 2012;54(2):136-45. doi: 10.1097/JOM.0b013e318244501e. PubMed PMID: 22261926; PubMed Central PMCID: PMCPMC3275688.

283.	Gallo LC, de Los Monteros KE, Allison M, Diez Roux A, Polak JF, Watson KE, et al. Do socioeconomic gradients in subclinical atherosclerosis vary according to acculturation level? Analyses of Mexican-Americans in the multi-ethnic study of atherosclerosis. Psychosom Med. 2009;71(7):756-62. Epub 20090806. doi: 10.1097/PSY.0b013e3181b0d2b4. PubMed PMID: 19661194; PubMed Central PMCID: PMCPMC2761426.

284.	Gold RS, Unkart JT, McClelland RL, Bertoni AG, Allison MA. Health insurance status and type associated with varying levels of glycemic control in the US: The multi-ethnic study of atherosclerosis (MESA). Primary Care Diabetes. 2020. doi: https://doi.org/10.1016/j.pcd.2020.11.011.

285.	Gutiérrez OM, Katz R, Peralta CA, de Boer IH, Siscovick D, Wolf M, et al. Associations of socioeconomic status and processed food intake with serum phosphorus concentration in community-living adults: the Multi-Ethnic Study of Atherosclerosis (MESA). J Ren Nutr. 2012;22(5):480-9. Epub 20120103. doi: 10.1053/j.jrn.2011.08.008. PubMed PMID: 22217539; PubMed Central PMCID: PMCPMC3321388.

286.	Hajat A, Diez-Roux AV, Adar SD, Auchincloss AH, Lovasi GS, O'Neill MS, et al. Air pollution and individual and neighborhood socioeconomic status: evidence from the Multi-Ethnic Study of Atherosclerosis (MESA). Environ Health Perspect. 2013;121(11-12):1325-33. Epub 2013/10/01. doi: 10.1289/ehp.1206337. PubMed PMID: 24076625; PubMed Central PMCID: PMCPMC3855503 in this document are solely those of the authors and the U.S. EPA does not endorse any products or commercial services mentioned in this publication. The authors declare they have no actual or potential competing financial interests.

287.	Hicken MT, Adar SD, Diez Roux AV, O'Neill MS, Magzamen S, Auchincloss AH, et al. Do Psychosocial Stress and Social Disadvantage Modify the Association Between Air Pollution and Blood Pressure?: The Multi-Ethnic Study of Atherosclerosis. American Journal of Epidemiology. 2013;178(10):1550-62. doi: 10.1093/aje/kwt190.

288.	Jones SA, Li Q, Aiello AE, O'Rand AM, Evenson KR. Physical Activity, Sedentary Behavior, and Retirement: The Multi-Ethnic Study of Atherosclerosis. Am J Prev Med. 2018;54(6):786-94. Epub 20180409. doi: 10.1016/j.amepre.2018.02.022. PubMed PMID: 29650285; PubMed Central PMCID: PMCPMC5962425.

289.	Lemelin ET, Diez Roux AV, Franklin TG, Carnethon M, Lutsey PL, Ni H, et al. Life-course socioeconomic positions and subclinical atherosclerosis in the multi-ethnic study of atherosclerosis. Soc Sci Med. 2009;68(3):444-51. Epub 2008/12/17. doi: 10.1016/j.socscimed.2008.10.038. PubMed PMID: 19081660; PubMed Central PMCID: PMCPMC5444463.

290.	Lê-Scherban F, Brenner AB, Hicken MT, Needham BL, Seeman T, Sloan RP, et al. Child and Adult Socioeconomic Status and the Cortisol Response to Acute Stress: Evidence From the Multi-Ethnic Study of Atherosclerosis. Psychosom Med. 2018;80(2):184-92. doi: 10.1097/psy.0000000000000543. PubMed PMID: 29215456; PubMed Central PMCID: PMCPMC5794563.

291.	Lovasi GS, Diez Roux AV, Hoffman EA, Smith LJ, Jiang R, Carr JJ, et al. Socioeconomic status is positively associated with percent emphysema on CT scan: The MESA lung study. Acad Radiol. 2011;18(2):199-204. doi: 10.1016/j.acra.2010.10.010. PubMed PMID: 21232685; PubMed Central PMCID: PMCPMC3038582.

292.	Merkin SS, Karlamangla A, Roux AV, Shrager S, Seeman TE. Life course socioeconomic status and longitudinal accumulation of allostatic load in adulthood: multi-ethnic study of atherosclerosis. Am J Public Health. 2014;104(4):e48-55. Epub 20140213. doi: 10.2105/ajph.2013.301841. PubMed PMID: 24524526; PubMed Central PMCID: PMCPMC4025706.

293.	Nazzal C, Shea S, Castro-Diehl C, Alfaro T, Frenz P, Rodriguez CJ. Educational Inequalities in Cardiovascular Risk Factor and Blood Pressure Control in the Elderly: Comparison of MESA Cohort and Chilean NHS Survey Outcome Measures. Glob Heart. 2018;13(1):19-26. Epub 20171113. doi: 10.1016/j.gheart.2017.09.001. PubMed PMID: 29146490.

294.	Needham BL, Smith JA, Zhao W, Wang X, Mukherjee B, Kardia SL, et al. Life course socioeconomic status and DNA methylation in genes related to stress reactivity and inflammation: The multi-ethnic study of atherosclerosis. Epigenetics. 2015;10(10):958-69. doi: 10.1080/15592294.2015.1085139. PubMed PMID: 26295359; PubMed Central PMCID: PMCPMC4844216.

295.	Park JW, Dulin AJ, Needham BL, Sims M, Loucks EB, Fava JL, et al. Examining Optimism, Psychosocial Risks, and Cardiovascular Health Using Life's Simple 7 Metrics in the Multi-Ethnic Study of Atherosclerosis and the Jackson Heart Study. Front Cardiovasc Med. 2021;8:788194. Epub 20211215. doi: 10.3389/fcvm.2021.788194. PubMed PMID: 34977194; PubMed Central PMCID: PMCPMC8714850.

296.	Park JW, Howe CJ, Dionne LA, Scarpaci MM, Needham BL, Sims M, et al. Social support, psychosocial risks, and cardiovascular health: Using harmonized data from the Jackson Heart Study, Mediators of Atherosclerosis in South Asians Living in America Study, and Multi-Ethnic Study of Atherosclerosis. SSM Popul Health. 2022;20:101284. Epub 2022/11/18. doi: 10.1016/j.ssmph.2022.101284. PubMed PMID: 36387018; PubMed Central PMCID: PMCPMC9646650.

297.	Peplinski B, McClelland R, Szklo M. Associations between socioeconomic status markers and depressive symptoms by race and gender: results from the Multi-Ethnic Study of Atherosclerosis (MESA). Ann Epidemiol. 2018;28(8):535-42.e1. Epub 20180522. doi: 10.1016/j.annepidem.2018.05.005. PubMed PMID: 29934242.

298.	Schmitz LL, Zhao W, Ratliff SM, Goodwin J, Miao J, Lu Q, et al. The Socioeconomic Gradient in Epigenetic Ageing Clocks: Evidence from the Multi-Ethnic Study of Atherosclerosis and the Health and Retirement Study. Epigenetics. 2022;17(6):589-611. Epub 2021/07/07. doi: 10.1080/15592294.2021.1939479. PubMed PMID: 34227900; PubMed Central PMCID: PMCPMC9235889.

299.	Seeman T, Thomas D, Merkin SS, Moore K, Watson K, Karlamangla A. The Great Recession worsened blood pressure and blood glucose levels in American adults. Proc Natl Acad Sci U S A. 2018;115(13):3296-301. Epub 20180312. doi: 10.1073/pnas.1710502115. PubMed PMID: 29531048; PubMed Central PMCID: PMCPMC5879652.

300.	Smith JA, Zhao W, Yasutake K, August C, Ratliff SM, Faul JD, et al. Gene-by-Psychosocial Factor Interactions Influence Diastolic Blood Pressure in European and African Ancestry Populations: Meta-Analysis of Four Cohort Studies. Int J Environ Res Public Health. 2017;14(12). doi: 10.3390/ijerph14121596. PubMed PMID: 29258278.

301.	Spalt EW, Curl CL, Allen RW, Cohen M, Williams K, Hirsch JA, et al. Factors influencing time-location patterns and their impact on estimates of exposure: the Multi-Ethnic Study of Atherosclerosis and Air Pollution (MESA Air). J Expo Sci Environ Epidemiol. 2016;26(4):341-8. Epub 20150429. doi: 10.1038/jes.2015.26. PubMed PMID: 25921081; PubMed Central PMCID: PMCPMC4641036.

302.	Tomey K, Diez Roux AV, Clarke P, Seeman T. Associations between neighborhood characteristics and self-rated health: a cross-sectional investigation in the Multi-Ethnic Study of Atherosclerosis (MESA) cohort. Health Place. 2013;24:267-74. Epub 20131012. doi: 10.1016/j.healthplace.2013.10.001. PubMed PMID: 24211514; PubMed Central PMCID: PMCPMC3874143.

303.	Wang YZ, Zhao W, Ammous F, Song Y, Du J, Shang L, et al. DNA Methylation Mediates the Association Between Individual and Neighborhood Social Disadvantage and Cardiovascular Risk Factors. Front Cardiovasc Med. 2022;9:848768. Epub 20220519. doi: 10.3389/fcvm.2022.848768. PubMed PMID: 35665255; PubMed Central PMCID: PMCPMC9162507.

304.	Zhao W, Ware EB, He Z, Kardia SLR, Faul JD, Smith JA. Interaction between Social/Psychosocial Factors and Genetic Variants on Body Mass Index: A Gene-Environment Interaction Analysis in a Longitudinal Setting. Int J Environ Res Public Health. 2017;14(10). Epub 20170929. doi: 10.3390/ijerph14101153. PubMed PMID: 28961216; PubMed Central PMCID: PMCPMC5664654.

305.	Allen NB, Diez-Roux A, Liu K, Bertoni AG, Szklo M, Daviglus M. Association of health professional shortage areas and cardiovascular risk factor prevalence, awareness, and control in the Multi-Ethnic Study of Atherosclerosis (MESA). Circ Cardiovasc Qual Outcomes. 2011;4(5):565-72. Epub 20110830. doi: 10.1161/circoutcomes.111.960922. PubMed PMID: 21878669; PubMed Central PMCID: PMCPMC3857032.

306.	Auchincloss AH, Diez Roux AV, Mujahid MS, Shen M, Bertoni AG, Carnethon MR. Neighborhood resources for physical activity and healthy foods and incidence of type 2 diabetes mellitus: the Multi-Ethnic study of Atherosclerosis. Arch Intern Med. 2009;169(18):1698-704. doi: 10.1001/archinternmed.2009.302. PubMed PMID: 19822827; PubMed Central PMCID: PMCPMC2828356.

307.	Auchincloss AH, Li J, Moore KA, Franco M, Mujahid MS, Moore LV. Are neighbourhood restaurants related to frequency of restaurant meals and dietary quality? Prevalence and changes over time in the Multi-Ethnic Study of Atherosclerosis. Public Health Nutr. 2021;24(14):4630-41. Epub 20210525. doi: 10.1017/s1368980021002196. PubMed PMID: 34030763; PubMed Central PMCID: PMCPMC8688147.

308.	Auchincloss AH, Mujahid MS, Shen M, Michos ED, Whitt-Glover MC, Diez Roux AV. Neighborhood health-promoting resources and obesity risk (the multi-ethnic study of atherosclerosis). Obesity (Silver Spring). 2013;21(3):621-8. doi: 10.1002/oby.20255. PubMed PMID: 23592671; PubMed Central PMCID: PMCPMC3511654.

309.	Auchincloss AH, Roux AVD, Brown DG, Erdmann CA, Bertoni AG. Neighborhood resources for physical activity and healthy foods and their association with insulin resistance. Epidemiology. 2008;19(1):146-57. doi: 10.1097/EDE.0b013e31815c480. PubMed PMID: WOS:000251889400022.

310.	Baek J, Hirsch JA, Moore K, Tabb LP, Barrientos-Gutierrez T, Lisabeth LD, et al. Statistical Methods to Study Variation in Associations Between Food Store Availability and Body Mass in the Multi-Ethnic Study of Atherosclerosis. Epidemiology. 2017;28(3):403-11. doi: 10.1097/ede.0000000000000631. PubMed PMID: 28145983; PubMed Central PMCID: PMCPMC5378605.

311.	Barrientos-Gutierrez T, Moore KAB, Auchincloss AH, Mujahid MS, August C, Sanchez BN, et al. Neighborhood Physical Environment and Changes in Body Mass Index: Results From the Multi-Ethnic Study of Atherosclerosis. Am J Epidemiol. 2017;186(11):1237-45. doi: 10.1093/aje/kwx186. PubMed PMID: 29206987; PubMed Central PMCID: PMCPMC5860514.

312.	Berchuck SI, Warren JL, Herring AH, Evenson KR, Moore KAB, Ranchod YK, et al. Spatially modelling the association between access to recreational facilities and exercise: the 'Multi-ethnic study of atherosclerosis'. Journal of the Royal Statistical Society Series a-Statistics in Society. 2016;179(1):293-310. doi: 10.1111/rssa.12119. PubMed PMID: WOS:000368352800011.

313.	Besser L, Galvin JE, Rodriguez D, Seeman T, Kukull W, Rapp SR, et al. Associations between neighborhood built environment and cognition vary by apolipoprotein E genotype: Multi-Ethnic Study of Atherosclerosis. Health & Place. 2019;60(102188):102188. doi: ARTN 102188 10.1016/j.healthplace.2019.102188. PubMed PMID: WOS:000500938700004.

314.	Billings ME, Johnson DA, Simonelli G, Moore K, Patel SR, Diez Roux AV, et al. Neighborhood Walking Environment and Activity Level Are Associated With OSA: The Multi-Ethnic Study of Atherosclerosis. Chest. 2016;150(5):1042-9. Epub 20160618. doi: 10.1016/j.chest.2016.06.012. PubMed PMID: 27327117; PubMed Central PMCID: PMCPMC5103016.

315.	Christine PJ, Auchincloss AH, Bertoni AG, Carnethon MR, Sanchez BN, Moore K, et al. Longitudinal Associations Between Neighborhood Physical and Social Environments and Incident Type 2 Diabetes Mellitus: The Multi-Ethnic Study of Atherosclerosis (MESA. JAMA Intern Med. 2015;175(8):1311–20.

316.	Desantis AS, Diez Roux AV, Moore K, Baron KG, Mujahid MS, Nieto FJ. Associations of neighborhood characteristics with sleep timing and quality: the Multi-Ethnic Study Of Atherosclerosis. Sleep. 2013;36(10):1543-51. Epub 20131001. doi: 10.5665/sleep.3054. PubMed PMID: 24082314; PubMed Central PMCID: PMCPMC3773204.

317.	Doubleday A, Knott CJ, Hazlehurst MF, Bertoni AG, Kaufman JD, Hajat A. Neighborhood greenspace and risk of type 2 diabetes in a prospective cohort: the Multi-Ethncity Study of Atherosclerosis. Environ Health. 2022;21(1):18. Epub 20220116. doi: 10.1186/s12940-021-00824-w. PubMed PMID: 35034636; PubMed Central PMCID: PMCPMC8762964.

318.	Franco M, Diez-Roux AV, Nettleton JA, Lazo M, Brancati F, Caballero B, et al. Availability of healthy foods and dietary patterns: the Multi-Ethnic Study of Atherosclerosis. American Journal of Clinical Nutrition. 2009;89(3):897-904. doi: 10.3945/ajcn.2008.26434. PubMed PMID: WOS:000263571300025.

319.	Garg PK, Jorgensen N, Diez-Roux AV, O'Neal W, Mujahid M, Moore K, et al. Neighborhood environments and risk of incident atrial fibrillation: The Multi-Ethnic Study of Atherosclerosis. Eur J Prev Cardiol. 2020;27(19):2116–8.

320.	He Z, Zhang M, Lee S, Smith JA, Kardia SLR, Diez Roux AV, et al. Set-Based Tests for the Gene-Environment Interaction in Longitudinal Studies. J Am Stat Assoc. 2017;112(519):966-78. Epub 20161216. doi: 10.1080/01621459.2016.1252266. PubMed PMID: 29780190; PubMed Central PMCID: PMCPMC5954413.

321.	Hirsch JA, Diez Roux AV, Moore KA, Evenson KR, Rodriguez DA. Change in walking and body mass index following residential relocation: the multi-ethnic study of atherosclerosis. Am J Public Health. 2014;104(3):e49-56. Epub 2014/01/18. doi: 10.2105/AJPH.2013.301773. PubMed PMID: 24432935; PubMed Central PMCID: PMCPMC3953773.

322.	Hirsch JA, Diez Roux AV, Rodriguez DA, Brines SJ, Moore KA. Discrete land uses and transportation walking in two U.S. cities: the Multi-Ethnic Study of Atherosclerosis. Health Place. 2013;24:196-202. Epub 20131008. doi: 10.1016/j.healthplace.2013.09.007. PubMed PMID: 24148201; PubMed Central PMCID: PMCPMC3851044.

323.	Hirsch JA, Moore KA, Barrientos-Gutierrez T, Brines SJ, Zagorski MA, Rodriguez DA, et al. Built environment change and change in BMI and waist circumference: Multi-ethnic Study of Atherosclerosis. Obesity (Silver Spring). 2014;22(11):2450-7. Epub 2014/08/20. doi: 10.1002/oby.20873. PubMed PMID: 25136965; PubMed Central PMCID: PMCPMC4224985.

324.	Hirsch JA, Moore KA, Clarke PJ, Rodriguez DA, Evenson KR, Brines SJ, et al. Changes in the built environment and changes in the amount of walking over time: longitudinal results from the multi-ethnic study of atherosclerosis. Am J Epidemiol. 2014;180(8):799-809. doi: 10.1093/aje/kwu218. PubMed PMID: 25234431.

325.	Hirsch JA, Moore KA, Evenson KR, Rodriguez DA, Diez Roux AV. Walk Score(R) and Transit Score(R) and walking in the multi-ethnic study of atherosclerosis. Am J Prev Med. 2013;45(2):158-66. doi: 10.1016/j.amepre.2013.03.018. PubMed PMID: 23867022.

326.	Kaiser P, Auchincloss AH, Moore K, Sanchez BN, Berrocal V, Allen N, et al. Associations of neighborhood socioeconomic and racial/ethnic characteristics with changes in survey-based neighborhood quality, 2000-2011. Health & Place. 2016;42:30-6. doi: 10.1016/j.healthplace.2016.08.001. PubMed PMID: WOS:000389161000004.

327.	Kaiser P, Diez Roux AV, Mujahid M, Carnethon M, Bertoni A, Adar SD, et al. Neighborhood Environments and Incident Hypertension in the Multi-Ethnic Study of Atherosclerosis. Am J Epidemiol. 2016;183(11):988-97. Epub 20160508. doi: 10.1093/aje/kwv296. PubMed PMID: 27188946; PubMed Central PMCID: PMCPMC4887578.

328.	Li J, Auchincloss AH, Hirsch JA, Melly SJ, Moore KA, Peterson A, et al. Exploring the spatial scale effects of built environments on transport walking: Multi-Ethnic Study of Atherosclerosis. Health Place. 2022;73(102722):102722. doi: 10.1016/j.healthplace.2021.102722. PubMed PMID: 34864555.

329.	Li JJ, Peterson A, Auchincloss AH, Hirsch JA, Rodriguez DA, Melly SJ, et al. Comparing effects of Euclidean buffers and network buffers on associations between built environment and transport walking: the Multi-Ethnic Study of Atherosclerosis. International Journal of Health Geographics. 2022;21(1). doi: ARTN 12 10.1186/s12942-022-00310-7. PubMed PMID: WOS:000854862700001.

330.	Mair C, Diez Roux AV, Golden SH, Rapp S, Seeman T, Shea S. Change in neighborhood environments and depressive symptoms in New York City: the Multi-Ethnic Study of Atherosclerosis. Health Place. 2015;32:93-8. Epub 20150207. doi: 10.1016/j.healthplace.2015.01.003. PubMed PMID: 25665936; PubMed Central PMCID: PMCPMC4381537.

331.	Maurer Braun L, Rodriguez DA, Evenson KR, Hirsch JA, Moore KA, Diez Roux AV. Walkability and cardiometabolic risk factors: Cross-sectional and longitudinal associations from the Multi-Ethnic Study of Atherosclerosis. Health Place. 2016;39:9–17.

332.	Moore K, Diez Roux AV, Auchincloss A, Evenson KR, Kaufman J, Mujahid M, et al. Home and work neighbourhood environments in relation to body mass index: the Multi-Ethnic Study of Atherosclerosis (MESA). J Epidemiol Community Health. 2013;67(10):846-53. Epub 2013/07/23. doi: 10.1136/jech-2013-202682. PubMed PMID: 23868527; PubMed Central PMCID: PMCPMC3779875.

333.	Moore KA, Hirsch JA, August C, Mair C, Sanchez BN, Diez Roux AV. Neighborhood Social Resources and Depressive Symptoms: Longitudinal Results from the Multi-Ethnic Study of Atherosclerosis. J Urban Health. 2016;93(3):572-88. Epub 2016/04/24. doi: 10.1007/s11524-016-0042-0. PubMed PMID: 27106865; PubMed Central PMCID: PMCPMC4899327.

334.	Moore LV, Diez Roux AV, Nettleton JA, Jacobs DR, Jr. Associations of the local food environment with diet quality--a comparison of assessments based on surveys and geographic information systems: the multi-ethnic study of atherosclerosis. Am J Epidemiol. 2008;167(8):917-24. Epub 20080227. doi: 10.1093/aje/kwm394. PubMed PMID: 18304960; PubMed Central PMCID: PMCPMC2587217.

335.	Moore LV, Diez Roux AV, Nettleton JA, Jacobs DR, Franco M. Fast-food consumption, diet quality, and neighborhood exposure to fast food: the multi-ethnic study of atherosclerosis. Am J Epidemiol. 2009;170(1):29-36. Epub 20090508. doi: 10.1093/aje/kwp090. PubMed PMID: 19429879; PubMed Central PMCID: PMCPMC2733038.

336.	Mujahid MS, Diez Roux AV, Morenoff JD, Raghunathan TE, Cooper RS, Ni H, et al. Neighborhood characteristics and hypertension. Epidemiology. 2008;19(4):590-8. Epub 2008/05/16. doi: 10.1097/EDE.0b013e3181772cb2. PubMed PMID: 18480733.

337.	Mujahid MS, Roux AVD, Shen MW, Gowda D, Sanchez B, Shea S, et al. Relation between neighborhood environments and obesity in the multi-ethnic study of atherosclerosis. American Journal of Epidemiology. 2008;167(11):1349-57. doi: 10.1093/aje/kwn047. PubMed PMID: WOS:000256169100011.

338.	Nethery RC, Warren JL, Herring AH, Moore KA, Evenson KR, Diez-Roux AV. A common spatial factor analysis model for measured neighborhood-level characteristics: The Multi-Ethnic Study of Atherosclerosis. Health Place. 2015;36:35-46. Epub 20150912. doi: 10.1016/j.healthplace.2015.08.009. PubMed PMID: 26372887; PubMed Central PMCID: PMCPMC4679666.

339.	Ranchod YK, Roux AVD, Evenson KR, Sanchez BN, Moore K. Longitudinal Associations Between Neighborhood Recreational Facilities and Change in Recreational Physical Activity in the Multi-Ethnic Study of Atherosclerosis, 2000-2007. American Journal of Epidemiology. 2014;179(3):335-43. doi: 10.1093/aje/kwt263. PubMed PMID: WOS:000330190600008.

340.	Remigio-Baker RA, Roux AVD, Szklo M, Crum RM, Leoutsakos JM, Franco M, et al. Physical Environment May Modify the Association Between Depressive Symptoms and Change in Waist Circumference: The Multi-Ethnic Study of Atherosclerosis. Psychosomatics. 2014;55(2):144-54. doi: DOI 10.1016/j.psym.2013.10.008. PubMed PMID: WOS:000333085600004.

341.	Rodriguez DA, Evenson KR, Diez Roux AV, Brines SJ. Land use, residential density, and walking. The multi-ethnic study of atherosclerosis. Am J Prev Med. 2009;37(5):397-404. doi: 10.1016/j.amepre.2009.07.008. PubMed PMID: 19840694.

342.	Tomey K, Roux AVD, Clarke P, Seeman T. Associations between neighborhood characteristics and self-rated health: A cross-sectional investigation in the Multi-Ethnic Study of Atherosclerosis (MESA) cohort. Health & Place. 2013;24:267-74. doi: 10.1016/j.healthplace.2013.10.001. PubMed PMID: WOS:000326985300034.

343.	Tristão Parra M, De Moraes ACF, Nascimento-Ferreira MV, Mills PJ, Allison M. Longitudinal Associations of Physical Activity Patterns and the Environment: An 18-Year Follow-Up to the MESA Study. Int J Environ Res Public Health. 2022;19(17). Epub 20220901. doi: 10.3390/ijerph191710925. PubMed PMID: 36078632; PubMed Central PMCID: PMCPMC9518075.

344.	Unger E, Diez-Roux AV, Lloyd-Jones DM, Mujahid MS, Nettleton JA, Bertoni A, et al. Association of neighborhood characteristics with cardiovascular health in the multi-ethnic study of atherosclerosis. Circ Cardiovasc Qual Outcomes. 2014;7(4):524-31. Epub 2014/07/10. doi: 10.1161/CIRCOUTCOMES.113.000698. PubMed PMID: 25006187; PubMed Central PMCID: PMCPMC4172357.

345.	Wing JJ, August E, Adar SD, Dannenberg AL, Hajat A, Sanchez BN, et al. Change in Neighborhood Characteristics and Change in Coronary Artery Calcium A Longitudinal Investigation in the MESA (Multi-Ethnic Study of Atherosclerosis) Cohort. Circulation. 2016;134(7):504-13. doi: 10.1161/Circulationaha.115.020534. PubMed PMID: WOS:000382286000005.

346.	Diez Roux AV, Mujahid MS, Hirsch JA, Moore K, Moore LV. The Impact of Neighborhoods on CV Risk. Glob Heart. 2016;11(3):353-63. doi: 10.1016/j.gheart.2016.08.002. PubMed PMID: 27741982; PubMed Central PMCID: PMCPMC5098701.

347.	Franco M, Diez Roux AV, Glass TA, Caballero B, Brancati FL. Neighborhood characteristics and availability of healthy foods in Baltimore. Am J Prev Med. 2008;35(6):561-7. Epub 2008/10/10. doi: 10.1016/j.amepre.2008.07.003. PubMed PMID: 18842389; PubMed Central PMCID: PMCPMC4348113.

348.	Franco M, Diez-Roux AV, Nettleton JA, Lazo M, Brancati F, Caballero B, et al. Availability of healthy foods and dietary patterns: the Multi-Ethnic Study of Atherosclerosis. Am J Clin Nutr. 2009;89(3):897-904. Epub 20090114. doi: 10.3945/ajcn.2008.26434. PubMed PMID: 19144728; PubMed Central PMCID: PMCPMC2667662.

349.	Hirsch JA, Moore KA, Evenson KR, Rodriguez DA, Diez Roux AV. Walk Score® and Transit Score® and walking in the multi-ethnic study of atherosclerosis. Am J Prev Med. 2013;45(2):158-66. doi: 10.1016/j.amepre.2013.03.018. PubMed PMID: 23867022; PubMed Central PMCID: PMCPMC3769092.

350.	Johnson DA, Hirsch JA, Moore KA, Redline S, Diez Roux AV. Associations Between the Built Environment and Objective Measures of Sleep: The Multi-Ethnic Study of Atherosclerosis. Am J Epidemiol. 2018;187(5):941-50. Epub 2018/03/17. doi: 10.1093/aje/kwx302. PubMed PMID: 29547912; PubMed Central PMCID: PMCPMC5928457.

351.	Johnson DA, Simonelli G, Moore K, Billings M, Mujahid MS, Rueschman M, et al. The Neighborhood Social Environment and Objective Measures of Sleep in the Multi-Ethnic Study of Atherosclerosis. Sleep. 2017;40(1). doi: 10.1093/sleep/zsw016. PubMed PMID: 28364474; PubMed Central PMCID: PMCPMC6084744.

352.	Braun LM, Rodríguez DA, Evenson KR, Hirsch JA, Moore KA, Diez Roux AV. Walkability and cardiometabolic risk factors: Cross-sectional and longitudinal associations from the Multi-Ethnic Study of Atherosclerosis. Health Place. 2016;39:9-17. Epub 20160301. doi: 10.1016/j.healthplace.2016.02.006. PubMed PMID: 26922513; PubMed Central PMCID: PMCPMC5015685.

353.	Auchincloss AH, Diez Roux AV, Brown DG, Erdmann CA, Bertoni AG. Neighborhood resources for physical activity and healthy foods and their association with insulin resistance. Epidemiology. 2008;19(1):146-57. doi: 10.1097/EDE.0b013e31815c480. PubMed PMID: 18091002.

354.	Besser LM, Chang LC, Evenson KR, Hirsch JA, Michael YL, Galvin JE, et al. Associations Between Neighborhood Park Access and Longitudinal Change in Cognition in Older Adults: The Multi-Ethnic Study of Atherosclerosis. J Alzheimers Dis. 2021;82(1):221-33. doi: 10.3233/jad-210370. PubMed PMID: 34024841; PubMed Central PMCID: PMCPMC8579707.

355.	Besser LM, Hirsch J, Galvin JE, Renne J, Park J, Evenson KR, et al. Associations between neighborhood park space and cognition in older adults vary by US location: The Multi-Ethnic Study of Atherosclerosis. Health Place. 2020;66:102459. Epub 2020/10/13. doi: 10.1016/j.healthplace.2020.102459. PubMed PMID: 33045671.

356.	Hirsch JA, Grunwald HE, Miles KL, Michael YL. Development of an instrument to measure perceived gentrification for health research: Perceptions about changes in environments and residents (PACER). SSM Popul Health. 2021;15:100900. Epub 2021/09/07. doi: 10.1016/j.ssmph.2021.100900. PubMed PMID: 34485674; PubMed Central PMCID: PMCPMC8399084.

357.	Do DP, Diez Roux AV, Hajat A, Auchincloss AH, Merkin SS, Ranjit N, et al. Circadian rhythm of cortisol and neighborhood characteristics in a population-based sample: the Multi-Ethnic Study of Atherosclerosis. Health Place. 2011;17(2):625-32. Epub 2011/02/05. doi: 10.1016/j.healthplace.2010.12.019. PubMed PMID: 21292535; PubMed Central PMCID: PMCPMC3189702.

358.	Mayne SL, Auchincloss AH, Moore KA, Michael YL, Tabb LP, Echeverria SE, et al. Cross-sectional and longitudinal associations of neighbourhood social environment and smoking behaviour: the multiethnic study of atherosclerosis. J Epidemiol Community Health. 2017;71(4):396-403. Epub 2016/11/26. doi: 10.1136/jech-2016-207990. PubMed PMID: 27885050; PubMed Central PMCID: PMCPMC5772966.

359.	Wing JJ, August E, Adar SD, Dannenberg AL, Hajat A, Sanchez BN, et al. Change in Neighborhood Characteristics and Change in Coronary Artery Calcium: A Longitudinal Investigation in the MESA (Multi-Ethnic Study of Atherosclerosis) Cohort. Circulation. 2016;134(7):504-13. Epub 2016/08/17. doi: 10.1161/CIRCULATIONAHA.115.020534. PubMed PMID: 27528645; PubMed Central PMCID: PMCPMC4991627.

360.	Hailu EM, Lewis TT, Needham BL, Lin J, Seeman TE, Mujahid MS. Longitudinal Associations between Discrimination, Neighborhood Social Cohesion, and Telomere Length: The Multi-Ethnic Study of Atherosclerosis (MESA). J Gerontol A Biol Sci Med Sci. 2021. Epub 20210720. doi: 10.1093/gerona/glab193. PubMed PMID: 34282826.

361.	Ware EB, Smith JA, Mukherjee B, Lee S, Kardia SL, Diez-Roux AV. Applying Novel Methods for Assessing Individual- and Neighborhood-Level Social and Psychosocial Environment Interactions with Genetic Factors in the Prediction of Depressive Symptoms in the Multi-Ethnic Study of Atherosclerosis. Behav Genet. 2016;46(1):89-99. Epub 20150809. doi: 10.1007/s10519-015-9734-6. PubMed PMID: 26254610; PubMed Central PMCID: PMCPMC4720563.

362.	Brown R, Hailu EM, Needham BL, Roux AD, Seeman TE, Lin J, et al. Neighborhood social environment and changes in leukocyte telomere length: The Multi-Ethnic Study of Atherosclerosis (MESA). Health Place. 2021;67:102488. Epub 20201201. doi: 10.1016/j.healthplace.2020.102488. PubMed PMID: 33276262.

363.	Smith JA, Zhao W, Wang X, Ratliff SM, Mukherjee B, Kardia SLR, et al. Neighborhood characteristics influence DNA methylation of genes involved in stress response and inflammation: The Multi-Ethnic Study of Atherosclerosis. Epigenetics. 2017;12(8):662-73. Epub 20170705. doi: 10.1080/15592294.2017.1341026. PubMed PMID: 28678593; PubMed Central PMCID: PMCPMC5687339.

364.	Garg PK, Jorgensen N, Diez-Roux AV, O'Neal W, Mujahid M, Moore K, et al. Neighborhood environments and risk of incident atrial fibrillation: The Multi-Ethnic Study of Atherosclerosis. Eur J Prev Cardiol. 2020;27(19):2116-8. Epub 20190726. doi: 10.1177/2047487319866020. PubMed PMID: 31349772.

365.	Merkin SS, Karlamangla A, Roux AD, Shrager S, Watson K, Seeman T. Race/ethnicity, neighborhood socioeconomic status and cardio-metabolic risk. SSM Popul Health. 2020;11:100634. Epub 2020/08/11. doi: 10.1016/j.ssmph.2020.100634. PubMed PMID: 32775593; PubMed Central PMCID: PMCPMC7397689.

366.	Lê-Scherban F, Albrecht SS, Osypuk TL, Sánchez BN, Diez Roux AV. Neighborhood ethnic composition, spatial assimilation, and change in body mass index over time among Hispanic and Chinese immigrants: Multi-Ethnic Study of Atherosclerosis. Am J Public Health. 2014;104(11):2138-46. Epub 20140911. doi: 10.2105/ajph.2014.302154. PubMed PMID: 25211724; PubMed Central PMCID: PMCPMC4192094.

367.	Hicken MT, Katz R, Crews DC, Kramer HJ, Peralta CA. Neighborhood Social Context and Kidney Function Over Time: The Multi-Ethnic Study of Atherosclerosis. Am J Kidney Dis. 2019;73(5):585-95. Epub 20190114. doi: 10.1053/j.ajkd.2018.10.015. PubMed PMID: 30655114; PubMed Central PMCID: PMCPMC6594400.

368.	Needham BL, Carroll JE, Diez Roux AV, Fitzpatrick AL, Moore K, Seeman TE. Neighborhood characteristics and leukocyte telomere length: the Multi-Ethnic Study of Atherosclerosis. Health Place. 2014;28:167-72. Epub 20140522. doi: 10.1016/j.healthplace.2014.04.009. PubMed PMID: 24859373; PubMed Central PMCID: PMCPMC4096814.

369.	Murray ET, Diez Roux AV, Carnethon M, Lutsey PL, Ni H, O'Meara ES. Trajectories of neighborhood poverty and associations with subclinical atherosclerosis and associated risk factors: the multi-ethnic study of atherosclerosis. Am J Epidemiol. 2010;171(10):1099-108. doi: 10.1093/aje/kwq044. PubMed PMID: 20423931.

370.	Mair C, Diez Roux AV, Osypuk TL, Rapp SR, Seeman T, Watson KE. Is neighborhood racial/ethnic composition associated with depressive symptoms? The multi-ethnic study of atherosclerosis. Soc Sci Med. 2010;71(3):541-50. Epub 20100512. doi: 10.1016/j.socscimed.2010.04.014. PubMed PMID: 20541303; PubMed Central PMCID: PMCPMC2922985.

371.	Mujahid MS, Diez Roux AV, Shen M, Gowda D, Sánchez B, Shea S, et al. Relation between neighborhood environments and obesity in the Multi-Ethnic Study of Atherosclerosis. Am J Epidemiol. 2008;167(11):1349-57. Epub 20080325. doi: 10.1093/aje/kwn047. PubMed PMID: 18367469.

372.	Nazmi A, Diez Roux A, Ranjit N, Seeman TE, Jenny NS. Cross-sectional and longitudinal associations of neighborhood characteristics with inflammatory markers: findings from the multi-ethnic study of atherosclerosis. Health Place. 2010;16(6):1104-12. Epub 20100718. doi: 10.1016/j.healthplace.2010.07.001. PubMed PMID: 20667763; PubMed Central PMCID: PMCPMC2952703.

373.	Kaiser P, Auchincloss AH, Moore K, Sanchez BN, Berrocal V, Allen N, et al. Associations of neighborhood socioeconomic and racial/ethnic characteristics with changes in survey-based neighborhood quality, 2000-2011. Health Place. 2016;42:30-6. Epub 2016/09/11. doi: 10.1016/j.healthplace.2016.08.001. PubMed PMID: 27614064; PubMed Central PMCID: PMCPMC5116413.

374.	Hailu EM, Lewis TT, Needham BL, Lin J, Seeman TE, Mujahid MS. Longitudinal Associations Between Discrimination, Neighborhood Social Cohesion, and Telomere Length: The Multi-Ethnic Study of Atherosclerosis. J Gerontol A Biol Sci Med Sci. 2021. Epub 2021/08/31. doi: 10.1093/gerona/glab193. PubMed PMID: 34458916.

375.	Aaron CP, Hoffman EA, Kawut SM, Austin JHM, Budoff M, Michos ED, et al. Ambient air pollution and pulmonary vascular volume on computed tomography: the MESA Air Pollution and Lung cohort studies. Eur Respir J. 2019;53(6). Epub 20190605. doi: 10.1183/13993003.02116-2018. PubMed PMID: 31167881; PubMed Central PMCID: PMCPMC6910868.

376.	Adar SD, Chen YH, D'Souza JC, O'Neill MS, Szpiro AA, Auchincloss AH, et al. Longitudinal Analysis of Long-Term Air Pollution Levels and Blood Pressure: A Cautionary Tale from the Multi-Ethnic Study of Atherosclerosis. Environ Health Perspect. 2018;126(10):107003. doi: 10.1289/ehp2966. PubMed PMID: 30392401; PubMed Central PMCID: PMCPMC6371645.

377.	Adar SD, D'Souza J, Mendelsohn-Victor K, Jacobs DR, Cushman M, Sheppard L, et al. Markers of inflammation and coagulation after long-term exposure to coarse particulate matter: a cross-sectional analysis from the multi-ethnic study of atherosclerosis. Environ Health Perspect. 2015;123(6):541-8. Epub 20150120. doi: 10.1289/ehp.1308069. PubMed PMID: 25616153; PubMed Central PMCID: PMCPMC4455582.

378.	Adar SD, Kaufman JD, Diez-Roux AV, Hoffman EA, D'Souza J, Stukovsky KH, et al. Air pollution and percent emphysema identified by computed tomography in the Multi-Ethnic study of Atherosclerosis. Environ Health Perspect. 2015;123(2):144-51. Epub 20141010. doi: 10.1289/ehp.1307951. PubMed PMID: 25302408; PubMed Central PMCID: PMCPMC4314244.

379.	Adar SD, Klein R, Klein BE, Szpiro AA, Cotch MF, Wong TY, et al. Air Pollution and the microvasculature: a cross-sectional assessment of in vivo retinal images in the population-based multi-ethnic study of atherosclerosis (MESA). PLoS Med. 2010;7(11):e1000372. Epub 20101130. doi: 10.1371/journal.pmed.1000372. PubMed PMID: 21152417; PubMed Central PMCID: PMCPMC2994677.

380.	Adar SD, Sheppard L, Vedal S, Polak JF, Sampson PD, Diez Roux AV, et al. Fine particulate air pollution and the progression of carotid intima-medial thickness: a prospective cohort study from the multi-ethnic study of atherosclerosis and air pollution. PLoS Med. 2013;10(4):e1001430. Epub 20130423. doi: 10.1371/journal.pmed.1001430. PubMed PMID: 23637576; PubMed Central PMCID: PMCPMC3637008.

381.	Adhikari R, D'Souza J, Soliman EZ, Burke GL, Daviglus M, Dr J, Jr., et al. Long-term Course Particulate Matter Exposure and Heart Rate Variability in the Multi-ethnic Study of Atherosclerosis. Epidemiology. 2016;27(3):405–13.

382.	Al Rifai M, Greenland P, Blaha MJ, Michos ED, Nasir K, Miedema MD, et al. Factors of health in the protection against death and cardiovascular disease among adults with subclinical atherosclerosis. Am Heart J. 2018;198:180–8.

383.	Allen RW, Adar SD, Avol E, Cohen M, Curl CL, Larson T, et al. Modeling the residential infiltration of outdoor PM(2.5) in the Multi-Ethnic Study of Atherosclerosis and Air Pollution (MESA Air). Environ Health Perspect. 2012;120(6):824-30. Epub 20120222. doi: 10.1289/ehp.1104447. PubMed PMID: 22534026; PubMed Central PMCID: PMCPMC3385439.

384.	Allen RW, Criqui MH, Diez Roux AV, Allison M, Shea S, Detrano R, et al. Fine particulate matter air pollution, proximity to traffic, and aortic atherosclerosis. Epidemiology. 2009;20(2):254-64. doi: 10.1097/EDE.0b013e31819644cc. PubMed PMID: 19129730; PubMed Central PMCID: PMCPMC3292428.

385.	Allen RW, Davies H, Cohen MA, Mallach G, Kaufman JD, Adar SD. The spatial relationship between traffic-generated air pollution and noise in 2 US cities. Environmental Research. 2009;109(3):334-42. doi: 10.1016/j.envres.2008.12.006. PubMed PMID: WOS:000264704800018.

386.	Auchincloss AH, Roux AV, Dvonch JT, Brown PL, Barr RG, Daviglus ML, et al. Associations between Recent Exposure to Ambient Fine Particulate Matter and Blood Pressure in the Multi-Ethnic Study of Atherosclerosis (MESA. Environ Health Perspect. 2008;116(4):486–91.

387.	Av DR, Ah A, B A, Rg B, M C, T D, et al. Recent Exposure to Particulate Matter and C-reactive Protein Concentration in the Multi-Ethnic Study of Atherosclerosis. Am J Epidemiol. 2006;164(5):437–48.

388.	Bell G, Mora S, Greenland P, Tsai M, Gill E, Kaufman JD. Association of Air Pollution Exposures With High-Density Lipoprotein Cholesterol and Particle Number: The Multi-Ethnic Study of Atherosclerosis. Arterioscler Thromb Vasc Biol. 2017;37(5):976-82. Epub 20170413. doi: 10.1161/atvbaha.116.308193. PubMed PMID: 28408373; PubMed Central PMCID: PMCPMC5407952.

389.	Bergen S, Sheppard L, Sampson PD, Kim SY, Richards M, Vedal S, et al. A national prediction model for PM2.5 component exposures and measurement error-corrected health effect inference. Environ Health Perspect. 2013;121(9):1017-25. Epub 20130611. doi: 10.1289/ehp.1206010. PubMed PMID: 23757600; PubMed Central PMCID: PMCPMC3764074.

390.	Billings ME, Gold D, Szpiro A, Aaron CP, Jorgensen N, Gassett A, et al. The Association of Ambient Air Pollution with Sleep Apnea: The Multi-Ethnic Study of Atherosclerosis. Ann Am Thorac Soc. 2019;16(3):363-70. doi: 10.1513/AnnalsATS.201804-248OC. PubMed PMID: 30571166; PubMed Central PMCID: PMCPMC6394120.

391.	Brown KM, Diez-Roux AV, Smith JA, Needham BL, Mukherjee B, Ware EB, et al. Social regulation of inflammation related gene expression in the multi-ethnic study of atherosclerosis. Psychoneuroendocrinology. 2020;117(104654):104654. doi: 10.1016/j.psyneuen.2020.104654. PubMed PMID: 32387875.

392.	Brown R, Hailu EM, Needham BL, Roux AD, Seeman TE, Lin J, et al. Neighborhood social environment and changes in leukocyte telomere length: The Multi-Ethnic Study of Atherosclerosis (MESA). Health Place. 2021;67(102488):102488. doi: 10.1016/j.healthplace.2020.102488. PubMed PMID: 33276262.

393.	Chi GC, Liu Y, MacDonald JW, Barr RG, Donohue KM, Hensley MD, et al. Long-term outdoor air pollution and DNA methylation in circulating monocytes: results from the Multi-Ethnic Study of Atherosclerosis (MESA). Environ Health. 2016;15(1):119. Epub 20161201. doi: 10.1186/s12940-016-0202-4. PubMed PMID: 27903268; PubMed Central PMCID: PMCPMC5131503.

394.	Chi GC, Liu Y, MacDonald JW, Reynolds LM, Enquobahrie DA, Fitzpatrick AL, et al. Epigenome-wide analysis of long-term air pollution exposure and DNA methylation in monocytes: results from the Multi-Ethnic Study of Atherosclerosis. Epigenetics. 2022;17(3):297–313.

395.	Christine PJ, Moore K, Crawford ND, Barrientos-Gutierrez T, Sánchez BN, Seeman T, et al. Exposure to Neighborhood Foreclosures and Changes in Cardiometabolic Health: Results From MESA. Am J Epidemiol. 2017;185(2):106-14. Epub 20161216. doi: 10.1093/aje/kww186. PubMed PMID: 27986705; PubMed Central PMCID: PMCPMC5253973.

396.	Cohen MA, Adar SD, Allen RW, Avol E, Curl CL, Gould T, et al. Approach to estimating participant pollutant exposures in the Multi-Ethnic Study of Atherosclerosis and Air Pollution (MESA Air). Environ Sci Technol. 2009;43(13):4687-93. doi: 10.1021/es8030837. PubMed PMID: 19673252; PubMed Central PMCID: PMCPMC2727607.

397.	Cohen R, Gasca NC, McClelland RL, Alcantara C, Dr J, Jr., Diez Roux A, et al. Effect of Physical Activity on the Relation Between Psychosocial Factors and Cardiovascular Events (from the Multi-Ethnic Study of Atherosclerosis. Am J Cardiol. 2016;117(10):1545–51.

398.	Crawford ND, Moore K, Christine PJ, Barrientos-Gutierrez T, Seeman T, Roux AVD. Examining the Role of Neighborhood-Level Foreclosure in Smoking and Alcohol Use Among Older Adults in the Multi-Ethnic Study of Atherosclerosis. American Journal of Epidemiology. 2018;187(9):1863-70. doi: 10.1093/aje/kwy075. PubMed PMID: WOS:000443542000005.

399.	D'Souza J, Weuve J, Brook RD, Evans DA, Kaufman JD, Adar SD. Long-Term Exposures to Urban Noise and Blood Pressure Levels and Control Among Older Adults. Hypertension. 2021;78(6):1801-8. Epub 2021/10/26. doi: 10.1161/HYPERTENSIONAHA.121.17708. PubMed PMID: 34689591; PubMed Central PMCID: PMCPMC8585701.

400.	Delker E, AlYami B, Gallo LC, Ruiz JM, Szklo M, Allison MA. Chronic Stress Burden, Visceral Adipose Tissue, and Adiposity-Related Inflammation: The Multi-Ethnic Study of Atherosclerosis. Psychosom Med. 2021;83(8):834-42. doi: 10.1097/psy.0000000000000983. PubMed PMID: 34292207; PubMed Central PMCID: PMCPMC8490301.

401.	Diez Roux AV, Ranjit N, Powell L, Jackson S, Lewis TT, Shea S, et al. Psychosocial Factors and Coronary Calcium in Adults without Clinical Cardiovascular Disease. Ann Intern Med. 2006;144(11):822–31.

402.	Doney B, Hnizdo E, Graziani M, Kullman G, Burchfiel C, Baron S, et al. Occupational risk factors for COPD phenotypes in the Multi-Ethnic Study of Atherosclerosis (MESA) Lung Study. Copd. 2014;11(4):368-80. Epub 20140225. doi: 10.3109/15412555.2013.813448. PubMed PMID: 24568208; PubMed Central PMCID: PMCPMC4096066.

403.	Evenson KR, Block R, Diez Roux AV, McGinn AP, Wen F, Rodriguez DA. Associations of adult physical activity with perceived safety and police-recorded crime: the Multi-ethnic Study of Atherosclerosis. Int J Behav Nutr Phys Act. 2012;9(146):146. doi: 10.1186/1479-5868-9-146. PubMed PMID: 23245527.

404.	Everson-Rose SA, Roetker NS, Lutsey PL, Kershaw KN, Longstreth WT, Jr., Sacco RL, et al. Chronic stress, depressive symptoms, anger, hostility, and risk of stroke and transient ischemic attack in the multi-ethnic study of atherosclerosis. Stroke. 2014;45(8):2318-23. Epub 20140710. doi: 10.1161/strokeaha.114.004815. PubMed PMID: 25013018; PubMed Central PMCID: PMCPMC4131200.

405.	Garg PK, O'Neal WT, Diez-Roux AV, Alonso A, Soliman EZ, Heckbert S. Negative Affect and Risk of Atrial Fibrillation: MESA. J Am Heart Assoc. 2019;8(1):e010603. doi: 10.1161/jaha.118.010603. PubMed PMID: 30563392; PubMed Central PMCID: PMCPMC6405728.

406.	Gill EA, Curl CL, Adar SD, Allen RW, Auchincloss AH, O'Neill MS, et al. Air pollution and cardiovascular disease in the Multi-Ethnic Study of Atherosclerosis. Prog Cardiovasc Dis. 2011;53(5):353-60. doi: 10.1016/j.pcad.2011.02.001. PubMed PMID: 21414470; PubMed Central PMCID: PMCPMC4016948.

407.	Gronlund CJ, Sheppard L, Adar SD, O'Neill MS, Auchincloss A, Madrigano J, et al. Vulnerability to the Cardiovascular Effects of Ambient Heat in Six US Cities: Results from the Multi-Ethnic Study of Atherosclerosis (MESA). Epidemiology. 2018;29(6):756-64. doi: 10.1097/ede.0000000000000910. PubMed PMID: 30113342; PubMed Central PMCID: PMCPMC6233295.

408.	Hailu EM, Lewis TT, Needham BL, Lin J, Seeman TE, Mujahid MS. Longitudinal Associations between Discrimination, Neighborhood Social Cohesion, and Telomere Length: The Multi-Ethnic Study of Atherosclerosis (MESA. J Gerontol A Biol Sci Med Sci. 2021.

409.	Hailu EM, Needham BL, Lewis TT, Lin J, Seeman TE, Roux AD, et al. Discrimination, social support, and telomere length: the Multi-Ethnic Study of Atherosclerosis (MESA). Ann Epidemiol. 2020;42:58-63 e2. doi: 10.1016/j.annepidem.2019.12.009. PubMed PMID: 32070634.

410.	Hajat A, Allison M, Diez-Roux AV, Jenny NS, Jorgensen NW, Szpiro AA, et al. Long-term exposure to air pollution and markers of inflammation, coagulation, and endothelial activation: a repeat-measures analysis in the Multi-Ethnic Study of Atherosclerosis (MESA). Epidemiology. 2015;26(3):310-20. doi: 10.1097/ede.0000000000000267. PubMed PMID: 25710246; PubMed Central PMCID: PMCPMC4455899.

411.	Hajat A, Diez Roux AV, Castro-Diehl C, Cosselman K, Golden SH, Hazlehurst MF, et al. The Association between Long-Term Air Pollution and Urinary Catecholamines: Evidence from the Multi-Ethnic Study of Atherosclerosis. Environ Health Perspect. 2019;127(5):57007. doi: 10.1289/ehp3286. PubMed PMID: 31095432; PubMed Central PMCID: PMCPMC6791118.

412.	Hajat A, Hazlehurst MF, Golden SH, Merkin SS, Seeman T, Szpiro AA, et al. The cross-sectional and longitudinal association between air pollution and salivary cortisol: Evidence from the Multi-Ethnic Study of Atherosclerosis. Environ Int. 2019;131(105062):105062. doi: 10.1016/j.envint.2019.105062. PubMed PMID: 31491811.

413.	Hajat A, Moore K, Do DP, Merkin SS, Punjabi NM, Sanchez BN, et al. Examining the cross-sectional and longitudinal association between diurnal cortisol and neighborhood characteristics: Evidence from the multi-ethnic study of atherosclerosis. Health & Place. 2015;34:199-206. doi: 10.1016/j.healthplace.2015.05.017. PubMed PMID: WOS:000357475600025.

414.	Hazlehurst MF, Spalt EW, Nicholas TP, Curl CL, Davey ME, Burke GL, et al. Contribution of the in-vehicle microenvironment to individual ambient-source nitrogen dioxide exposure: the Multi-Ethnic Study of Atherosclerosis and Air Pollution. J Expo Sci Environ Epidemiol. 2018;28(4):371-80. Epub 20180306. doi: 10.1038/s41370-018-0025-1. PubMed PMID: 29511286; PubMed Central PMCID: PMCPMC6013355.

415.	Hicken MT, Adar SD, Diez Roux AV, O'Neill MS, Magzamen S, Auchincloss AH, et al. Do psychosocial stress and social disadvantage modify the association between air pollution and blood pressure?: the multi-ethnic study of atherosclerosis. Am J Epidemiol. 2013;178(10):1550–62.

416.	Jensen RA, Shea S, Ranjit N, Diez-Roux A, Wong TY, Klein R, et al. Psychosocial risk factors and retinal microvascular signs: the multi-ethnic study of atherosclerosis. Am J Epidemiol. 2010;171(5):522-31. Epub 20091224. doi: 10.1093/aje/kwp414. PubMed PMID: 20035010; PubMed Central PMCID: PMCPMC2842225.

417.	Jensen SS, Larson T, Deepti KC, Kaufman JD. Modeling traffic air pollution in street canyons in New York City for intra-urban exposure assessment in the US Multi-Ethnic Study of atherosclerosis and air pollution. Atmospheric Environment. 2009;43(30):4544-56. doi: 10.1016/j.atmosenv.2009.06.042. PubMed PMID: WOS:000270319400004.

418.	Jones MR, Magid HS, Al-Rifai M, McEvoy JW, Kaufman JD, Hinckley Stukovsky KD, et al. Secondhand Smoke Exposure and Subclinical Cardiovascular Disease: The Multi-Ethnic Study of Atherosclerosis. J Am Heart Assoc. 2016;5(12). Epub 20161219. doi: 10.1161/jaha.115.002965. PubMed PMID: 27993830; PubMed Central PMCID: PMCPMC5210438.

419.	Kaufman JD, Adar SD, Allen RW, Barr RG, Budoff MJ, Burke GL, et al. Prospective study of particulate air pollution exposures, subclinical atherosclerosis, and clinical cardiovascular disease: The Multi-Ethnic Study of Atherosclerosis and Air Pollution (MESA Air). Am J Epidemiol. 2012;176(9):825-37. Epub 20121004. doi: 10.1093/aje/kws169. PubMed PMID: 23043127; PubMed Central PMCID: PMCPMC3571256.

420.	Kaufman JD, Adar SD, Barr RG, Budoff M, Burke GL, Curl CL, et al. Association between air pollution and coronary artery calcification within six metropolitan areas in the USA (the Multi-Ethnic Study of Atherosclerosis and Air Pollution): a longitudinal cohort study. Lancet. 2016;388(10045):696-704. doi: 10.1016/S0140-6736(16)00378-0. PubMed PMID: WOS:000381268700029.

421.	Keller JP, Larson TV, Austin E, Barr RG, Sheppard L, Vedal S, et al. Pollutant composition modification of the effect of air pollution on progression of coronary artery calcium: the Multi-Ethnic Study of Atherosclerosis. Environ Epidemiol. 2018;2(3):e024. Epub 20180709. doi: 10.1097/ee9.0000000000000024. PubMed PMID: 30854505; PubMed Central PMCID: PMCPMC6402342.

422.	Keller JP, Olives C, Kim S, Sheppard L, Sampson PD, Szpiro AA, et al. A unified spatiotemporal modeling approach for predicting concentrations of multiple air pollutants in the multi-ethnic study of atherosclerosis and air pollution. Environ Health Perspect. 2015;123(4):301–9.

423.	Kerr Z, Evenson KR, Moore K, Block R, Diez Roux AV. Changes in walking associated with perceived neighborhood safety and police-recorded crime: The multi-ethnic study of atherosclerosis. Prev Med. 2015;73:88-93. Epub 20150124. doi: 10.1016/j.ypmed.2015.01.017. PubMed PMID: 25625690; PubMed Central PMCID: PMCPMC4937793.

424.	Kershaw KN, Lane-Cordova AD, Carnethon MR, Tindle HA, Liu K. Chronic Stress and Endothelial Dysfunction: The Multi-Ethnic Study of Atherosclerosis (MESA). Am J Hypertens. 2017;30(1):75-80. Epub 20160901. doi: 10.1093/ajh/hpw103. PubMed PMID: 27585566; PubMed Central PMCID: PMCPMC5155567.

425.	Kershaw KN, Lewis TT, Roux AVD, Jenny NS, Liu K, Penedo FJ, et al. Self-Reported Experiences of Discrimination and Inflammation Among Men and Women: The Multi-Ethnic Study of Atherosclerosis. Health Psychology. 2016;35(4):343-50. doi: 10.1037/hea0000331. PubMed PMID: WOS:000373477600005.

426.	Kim SY, Sheppard L, Bergen S, Szpiro AA, Sampson PD, Kaufman JD, et al. Prediction of fine particulate matter chemical components with a spatio-temporal model for the Multi-Ethnic Study of Atherosclerosis cohort. J Expo Sci Environ Epidemiol. 2016;26(5):520-8. Epub 20160518. doi: 10.1038/jes.2016.29. PubMed PMID: 27189258; PubMed Central PMCID: PMCPMC5104659.

427.	Kim SY, Sheppard L, Kaufman JD, Bergen S, Szpiro AA, Larson TV, et al. Individual-level concentrations of fine particulate matter chemical components and subclinical atherosclerosis: a cross-sectional analysis based on 2 advanced exposure prediction models in the multi-ethnic study of atherosclerosis. Am J Epidemiol. 2014;180(7):718-28. Epub 20140826. doi: 10.1093/aje/kwu186. PubMed PMID: 25164422; PubMed Central PMCID: PMCPMC4172166.

428.	Kirwa K, Eckert CM, Vedal S, Hajat A, Kaufman JD. Ambient air pollution and risk of respiratory infection among adults: evidence from the multiethnic study of atherosclerosis (MESA. BMJ Open Respir Res. 2021. doi: 10.1136/bmjresp-2020-00866.

429.	Kirwa K, Szpiro AA, Sheppard L, Sampson PD, Wang M, Keller JP, et al. Fine-Scale Air Pollution Models for Epidemiologic Research: Insights From Approaches Developed in the Multi-ethnic Study of Atherosclerosis and Air Pollution (MESA Air). Curr Environ Health Rep. 2021;8(2):113-26. doi: 10.1007/s40572-021-00310-y. PubMed PMID: 34086258; PubMed Central PMCID: PMCPMC8278964.

430.	Ko YA, Mukherjee B, Smith JA, Kardia SL, Allison M, Diez Roux AV. Classification and Clustering Methods for Multiple Environmental Factors in Gene-Environment Interaction: Application to the Multi-Ethnic Study of Atherosclerosis. Epidemiology. 2016;27(6):870-8. doi: 10.1097/ede.0000000000000548. PubMed PMID: 27479650; PubMed Central PMCID: PMCPMC5039086.

431.	Krishnan RM, Adar SD, Szpiro AA, Jorgensen NW, Van Hee VC, Barr RG, et al. Vascular responses to long- and short-term exposure to fine particulate matter: MESA Air (Multi-Ethnic Study of Atherosclerosis and Air Pollution). J Am Coll Cardiol. 2012;60(21):2158-66. Epub 20121024. doi: 10.1016/j.jacc.2012.08.973. PubMed PMID: 23103035; PubMed Central PMCID: PMCPMC3665082.

432.	Leary PJ, Kaufman JD, Barr RG, Bluemke DA, Curl CL, Hough CL, et al. Traffic-related air pollution and the right ventricle. The multi-ethnic study of atherosclerosis. Am J Respir Crit Care Med. 2014;189(9):1093-100. doi: 10.1164/rccm.201312-2298OC. PubMed PMID: 24593877; PubMed Central PMCID: PMCPMC4098110.

433.	Lin GM, Lloyd-Jones DM, Colangelo LA, Szklo M, Heckbert SR, Chen LY, et al. Secondhand tobacco smoke exposure, urine cotinine, and risk of incident atrial fibrillation: The multi-ethnic study of atherosclerosis. Prog Cardiovasc Dis. 2022;74:38-44. Epub 20221021. doi: 10.1016/j.pcad.2022.10.006. PubMed PMID: 36279945.

434.	Lindström J, Szpiro AA, Sampson PD, Oron AP, Richards M, Larson TV, et al. A Flexible Spatio-Temporal Model for Air Pollution with Spatial and Spatio-Temporal Covariates. Environ Ecol Stat. 2014;21(3):411-33. doi: 10.1007/s10651-013-0261-4. PubMed PMID: 25264424; PubMed Central PMCID: PMCPMC4174563.

435.	Liu YL, Szklo M, Davidson KW, Bathon JM, Giles JT. Differential Association of Psychosocial Comorbidities With Subclinical Atherosclerosis in Rheumatoid Arthritis. Arthritis Care Res (Hoboken). 2015;67(10):1335-44. doi: 10.1002/acr.22635. PubMed PMID: 26274015.

436.	Lovasi GS, Diez Roux AV, Hoffman EA, Kawut SM, Dr J, Jr., Barr RG. Association of Environmental Tobacco Smoke Exposure in Childhood With Early Emphysema in Adulthood Among Nonsmokers: The MESA-Lung Study. Am J Epidimiol. 2010;171(1):54–62.

437.	Mair C, Diez Roux AV, Shen M, Shea S, Seeman T, Echeverria S, et al. Cross-sectional and longitudinal associations of neighborhood cohesion and stressors with depressive symptoms in the multiethnic study of atherosclerosis. Ann Epidemiol. 2009;19(1):49-57. doi: 10.1016/j.annepidem.2008.10.002. PubMed PMID: 19064189.

438.	Mayne SL, Auchincloss AH, Moore KA, Michael YL, Tabb LP, Echeverria SE, et al. Cross-sectional and longitudinal associations of neighbourhood social environment and smoking behaviour: the multiethnic study of atherosclerosis. Journal of Epidemiology and Community Health. 2017;71(4):396-403. doi: 10.1136/jech-2016-207990. PubMed PMID: WOS:000399324800015.

439.	Meier HCS, Hussein M, Needham B, Barber S, Lin J, Seeman T, et al. Cellular response to chronic psychosocial stress: Ten-year longitudinal changes in telomere length in the Multi-Ethnic Study of Atherosclerosis. Psychoneuroendocrinology. 2019;107:70-81. Epub 20190429. doi: 10.1016/j.psyneuen.2019.04.018. PubMed PMID: 31112903; PubMed Central PMCID: PMCPMC6635040.

440.	Mezuk B, Diez Roux AV, Seeman T. Evaluating the buffering vs. direct effects hypotheses of emotional social support on inflammatory markers: the multi-ethnic study of atherosclerosis. Brain Behav Immun. 2010;24(8):1294-300. Epub 20100625. doi: 10.1016/j.bbi.2010.06.006. PubMed PMID: 20600815; PubMed Central PMCID: PMCPMC2949452.

441.	Miller KA, Spalt EW, Gassett AJ, Curl CL, Larson TV, Avol E, et al. Estimating ambient-origin PM(2.5) exposure for epidemiology: observations, prediction, and validation using personal sampling in the Multi-Ethnic Study of Atherosclerosis. J Expo Sci Environ Epidemiol. 2019;29(2):227-37. Epub 20180830. doi: 10.1038/s41370-018-0053-x. PubMed PMID: 30166581; PubMed Central PMCID: PMCPMC6380932.

442.	Ni Y, Tracy RP, Cornell E, Kaufman JD, Szpiro AA, Campen MJ, et al. Short-term exposure to air pollution and biomarkers of cardiovascular effect: A repeated measures study. Environ Pollut. 2021;279(116893):116893. doi: 10.1016/j.envpol.2021.116893. PubMed PMID: 33765506.

443.	O'Neill MS, Diez-Roux AV, Auchincloss AH, Franklin TG, Jacobs DR, Jr., Astor BC, et al. Airborne particulate matter exposure and urinary albumin excretion: the Multi-Ethnic Study of Atherosclerosis. Occup Environ Med. 2008;65(8):534-40. Epub 20071121. doi: 10.1136/oem.2007.035238. PubMed PMID: 18032533; PubMed Central PMCID: PMCPMC2893565.

444.	O'Neill MS, Diez-Roux AV, Auchincloss AH, Shen M, Lima JA, Polak JF, et al. Long-term exposure to airborne particles and arterial stiffness: the Multi-Ethnic Study of Atherosclerosis (MESA). Environ Health Perspect. 2011;119(6):844-51. Epub 20110118. doi: 10.1289/ehp.0901524. PubMed PMID: 21245016; PubMed Central PMCID: PMCPMC3114821.

445.	Pang Y, Jones MR, Tellez-Plaza M, Guallar E, Vaidya D, Post WS, et al. Association of Geography and Ambient Air Pollution with Urine Metal Concentrations in Six US Cities: The Multi-Ethnic Study of Atherosclerosis. Int J Environ Res Public Health. 2016;13(3). Epub 20160315. doi: 10.3390/ijerph13030324. PubMed PMID: 26999173; PubMed Central PMCID: PMCPMC4808987.

446.	Park JW, Dulin AJ, Needham BL, Sims M, Loucks EB, Fava JL, et al. Examining Optimism, Psychosocial Risks, and Cardiovascular Health Using Life's Simple 7 Metrics in the Multi-Ethnic Study of Atherosclerosis and the Jackson Heart Study. Front Cardiovasc Med. 2021;8(788195):788194. doi: 10.3389/fcvm.2021.788194. PubMed PMID: 34977194.

447.	Park JW, Howe CJ, Dionne LA, Scarpaci MM, Needham BL, Sims M, et al. Social support, psychosocial risks, and cardiovascular health: Using harmonized data from the Jackson Heart Study, Mediators of Atherosclerosis in South Asians Living in America Study, and Multi-Ethnic Study of Atherosclerosis. SSM Popul Health. 2022;20(101284):101284. doi: 10.1016/j.ssmph.2022.101284. PubMed PMID: 36387018.

448.	Park SK, Auchincloss AH, O'Neill MS, Prineas R, Correa JC, Keeler J, et al. Particulate air pollution, metabolic syndrome, and heart rate variability: the multi-ethnic study of atherosclerosis (MESA). Environ Health Perspect. 2010;118(10):1406-11. Epub 20100608. doi: 10.1289/ehp.0901778. PubMed PMID: 20529761; PubMed Central PMCID: PMCPMC2957920.

449.	Powell-Wiley TM, Moore K, Allen N, Block R, Evenson KR, Mujahid M, et al. Associations of Neighborhood Crime and Safety and With Changes in Body Mass Index and Waist Circumference: The Multi-Ethnic Study of Atherosclerosis. Am J Epidemiol. 2017;186(3):280-8. doi: 10.1093/aje/kwx082. PubMed PMID: 28472256; PubMed Central PMCID: PMCPMC5860351.

450.	Ranjit N, Diez-Roux AV, Shea S, Cushman M, Seeman T, Jackson SA, et al. Psychosocial factors and inflammation in the multi-ethnic study of atherosclerosis. Arch Intern Med. 2007;167(2):174-81. doi: 10.1001/archinte.167.2.174. PubMed PMID: 17242319.

451.	Reynolds LM, Magid HS, Chi GC, Lohman K, Barr RG, Kaufman JD, et al. Secondhand Tobacco Smoke Exposure Associations With DNA Methylation of the Aryl Hydrocarbon Receptor Repressor. Nicotine Tob Res. 2017;19(4):442-51. doi: 10.1093/ntr/ntw219. PubMed PMID: 27613907; PubMed Central PMCID: PMCPMC6075517.

452.	Rudolph KE, Sanchez BN, Stuart EA, Greenberg B, Fujishiro K, Wand GS, et al. Job Strain and Cortisol Diurnal Cycle in MESA: Accounting for Between- and Within-Day Variability. Am J Epidemiol. 2016;183(5):497–506.

453.	Sack C, Vedal S, Sheppard L, Raghu G, Barr RG, Podolanczuk A, et al. Air pollution and subclinical interstitial lung disease: the Multi-Ethnic Study of Atherosclerosis (MESA) air-lung study. Eur Respir J. 2017;50(6):1700 559. doi: 10.1183/13993003.00559-2017. PubMed PMID: 29217611.

454.	Sack CS, Doney BC, Podolanczuk AJ, Hooper LG, Seixas NS, Hoffman EA, et al. Occupational Exposures and Subclinical Interstitial Lung Disease. The MESA (Multi-Ethnic Study of Atherosclerosis) Air and Lung Studies. Am J Respir Crit Care Med. 2017;196(8):1031-9. doi: 10.1164/rccm.201612-2431OC. PubMed PMID: 28753039; PubMed Central PMCID: PMCPMC5649983.

455.	Sampson PD, Szpiro AA, Sheppard L, Lindstrom J, Kaufman JD. Pragmatic estimation of a spatio-temporal air quality model with irregular monitoring data. Atmospheric Environment. 2011;45(36):6593-606. doi: 10.1016/j.atmosenv.2011.04.073. PubMed PMID: WOS:000296220200011.

456.	Sun M, Kaufman JD, Kim SY, Larson TV, Gould TR, Polak JF, et al. Particulate matter components and subclinical atherosclerosis: common approaches to estimating exposure in a Multi-Ethnic Study of Atherosclerosis cross-sectional study. Environ Health. 2013;12(1):39. doi: 10.1186/1476-069X-12-39. PubMed PMID: 23641873.

457.	Tibuakuu M, Jones MR, Navas-Acien A, Zhao D, Guallar E, Gassett AJ, et al. Exposure to ambient air pollution and calcification of the mitral annulus and aortic valve: the multi-ethnic study of atherosclerosis (MESA). Environ Health. 2017;16(1):133. Epub 20171221. doi: 10.1186/s12940-017-0346-x. PubMed PMID: 29268751; PubMed Central PMCID: PMCPMC5740967.

458.	van Hee VC, Adar SD, Szpiro AA, Barr RG, Bluemke DA, Diez Roux AV, et al. Exposure to Traffic and Left Ventricular Mass and Function The Multi-Ethnic Study of Atherosclerosis. Am J Respir Crit Care Med. 2009;179(9):827–34.

459.	Van Hee VC, Adar SD, Szpiro AA, Barr RG, Roux AD, Bluemke DA, et al. Common Genetic Variation, Residential Proximity to Traffic Exposure, and Left Ventricular Mass: The Multi-Ethnic Study of Atherosclerosis. Environ Health Perspect. 2010;118(7):962–9.

460.	van Hee VC, Szpiro AA, Prineas R, Neyer J, Watson K, Siscovick D, et al. Association of long-term air pollution with ventricular conduction and repolarization abnormalities. Epidemiology. 2011;22(6):773–80.

461.	Wang M, Aaron CP, Madrigano J, Hoffman EA, Angelini E, Yang J, et al. Association Between Long-term Exposure to Ambient Air Pollution and Change in Quantitatively Assessed Emphysema and Lung Function. Jama-Journal of the American Medical Association. 2019;322(6):546-56. doi: 10.1001/jama.2019.10255. PubMed PMID: WOS:000481799500019.

462.	Wang M, Keller JP, Adar SD, Kim SY, Larson TV, Olives C, et al. Development of Long-term Spatiotemporal Models for Ambient Ozone in Six Metropolitan regions of the United States: The MSA Air Study. Atmos Environ. 1994;2015;123(A):79-87.

463.	Wang M, Sampson PD, Hu J, Kleeman M, Keller JP, Olives C, et al. Combining Land-Use Regression and Chemical Transport Modeling in a Spatiotemporal Geostatistical Model for Ozone and PM2.5. Environ Sci Technol. 2016;50(10):5111-8. doi: 10.1021/acs.est.5b06001. PubMed PMID: 27074524.

464.	Wang M, Sampson PD, Sheppard LE, Stein JH, Vedal S, Kaufman JD. Long-Term Exposure to Ambient Ozone and Progression of Subclinical Arterial Disease: The Multi-Ethnic Study of Atherosclerosis and Air Pollution. Environ Health Perspect. 2019;127(5):57001. doi: 10.1289/ehp3325. PubMed PMID: 31063398; PubMed Central PMCID: PMCPMC6791411.

465.	Wilton D, Szpiro A, Gould T, Larson T. Improving spatial concentration estimates for nitrogen oxides using a hybrid meteorological dispersion/land use regression model in Los Angeles, CA and Seattle, WA. Science of the Total Environment. 2010;408(5):1120-30. doi: 10.1016/j.scitotenv.2009.11.033. PubMed PMID: WOS:000274365000015.

466.	Zhang K, Larson TV, Gassett A, Szpiro AA, Daviglus M, Burke GL, et al. Characterizing spatial patterns of airborne coarse particulate (PM10-2.5) mass and chemical components in three cities: the multi-ethnic study of atherosclerosis. Environ Health Perspect. 2014;122(8):823-30. Epub 20140318. doi: 10.1289/ehp.1307287. PubMed PMID: 24642481; PubMed Central PMCID: PMCPMC4123025.

467.	Kaufman JD, Adar SD, Barr RG, Budoff M, Burke GL, Curl CL, et al. Association between air pollution and coronary artery calcification within six metropolitan areas in the USA (the Multi-Ethnic Study of Atherosclerosis and Air Pollution): a longitudinal cohort study. Lancet. 2016;388(10045):696-704. Epub 20160524. doi: 10.1016/s0140-6736(16)00378-0. PubMed PMID: 27233746; PubMed Central PMCID: PMCPMC5019949.

468.	Al Rifai M, Greenland P, Blaha MJ, Michos ED, Nasir K, Miedema MD, et al. Factors of health in the protection against death and cardiovascular disease among adults with subclinical atherosclerosis. Am Heart J. 2018;198:180-8. Epub 20171111. doi: 10.1016/j.ahj.2017.10.026. PubMed PMID: 29653643; PubMed Central PMCID: PMCPMC5901883.

469.	Feinstein M, Liu K, Ning H, Fitchett G, Lloyd-Jones DM. Burden of cardiovascular risk factors, subclinical atherosclerosis, and incident cardiovascular events across dimensions of religiosity: The multi-ethnic study of atherosclerosis. Circulation. 2010;121(5):659-66. Epub 20100125. doi: 10.1161/circulationaha.109.879973. PubMed PMID: 20100975; PubMed Central PMCID: PMCPMC2871276.

470.	Cohen R, Gasca NC, McClelland RL, Alcántara C, Jacobs DR, Jr., Diez Roux A, et al. Effect of Physical Activity on the Relation Between Psychosocial Factors and Cardiovascular Events (from the Multi-Ethnic Study of Atherosclerosis). Am J Cardiol. 2016;117(10):1545-51. Epub 20160302. doi: 10.1016/j.amjcard.2016.02.026. PubMed PMID: 27017324; PubMed Central PMCID: PMCPMC4856538.

471.	Samuel LJ, Dennison Himmelfarb CR, Szklo M, Seeman TE, Echeverria SE, Diez Roux AV. Social engagement and chronic disease risk behaviors: the Multi-Ethnic Study of Atherosclerosis. Prev Med. 2015;71:61-6. Epub 20141215. doi: 10.1016/j.ypmed.2014.12.008. PubMed PMID: 25524614; PubMed Central PMCID: PMCPMC4329061.

472.	Mezuk B, Choi M, DeSantis AS, Rapp SR, Diez Roux AV, Seeman T. Loneliness, Depression, and Inflammation: Evidence from the Multi-Ethnic Study of Atherosclerosis. PLoS One. 2016;11(7):e0158056. Epub 20160701. doi: 10.1371/journal.pone.0158056. PubMed PMID: 27367428; PubMed Central PMCID: PMCPMC4930171.

473.	Carroll JE, Diez Roux AV, Fitzpatrick AL, Seeman T. Low social support is associated with shorter leukocyte telomere length in late life: multi-ethnic study of atherosclerosis. Psychosom Med. 2013;75(2):171-7. Epub 20130131. doi: 10.1097/PSY.0b013e31828233bf. PubMed PMID: 23370895; PubMed Central PMCID: PMCPMC3881963.

474.	Hailu EM, Needham BL, Lewis TT, Lin J, Seeman TE, Roux AD, et al. Discrimination, social support, and telomere length: the Multi-Ethnic Study of Atherosclerosis (MESA). Ann Epidemiol. 2020;42:58-63.e2. Epub 20191231. doi: 10.1016/j.annepidem.2019.12.009. PubMed PMID: 32070634.

475.	Ohira T, Diez Roux AV, Prineas RJ, Kizilbash MA, Carnethon MR, Folsom AR. Associations of psychosocial factors with heart rate and its short-term variability: multi-ethnic study of atherosclerosis. Psychosom Med. 2008;70(2):141-6. Epub 20080206. doi: 10.1097/PSY.0b013e318160686a. PubMed PMID: 18256350.

476.	Patel J, Mehta A, Rifai MA, Blaha MJ, Nasir K, McEvoy JW, et al. Hypertension guidelines and coronary artery calcification among South Asians: Results from MASALA and MESA. Am J Prev Cardiol. 2021;6:100158. Epub 20210212. doi: 10.1016/j.ajpc.2021.100158. PubMed PMID: 34327495; PubMed Central PMCID: PMCPMC8315395.

477.	Fujiyoshi A, Miura K, Ohkubo T, Kadowaki T, Kadowaki S, Zaid M, et al. Cross-sectional comparison of coronary artery calcium scores between Caucasian men in the United States and Japanese men in Japan: the multi-ethnic study of atherosclerosis and the Shiga epidemiological study of subclinical atherosclerosis. Am J Epidemiol. 2014;180(6):590-8. Epub 20140813. doi: 10.1093/aje/kwu169. PubMed PMID: 25125689; PubMed Central PMCID: PMCPMC4157702.

478.	Yamagishi K, Ohira T, Nakano H, Bielinski SJ, Sakurai S, Imano H, et al. Cross-cultural comparison of the sleep-disordered breathing prevalence among Americans and Japanese. Eur Respir J. 2010;36(2):379-84. Epub 20100128. doi: 10.1183/09031936.00118609. PubMed PMID: 20110399; PubMed Central PMCID: PMCPMC2895689.

479.	Barfield R, Wang H, Liu Y, Brody JA, Swenson B, Li R, et al. Epigenome-wide association analysis of daytime sleepiness in the Multi-Ethnic Study of Atherosclerosis reveals African-American-specific associations. Sleep. 2019;42(8). doi: 10.1093/sleep/zsz101. PubMed PMID: 31139831; PubMed Central PMCID: PMCPMC6685317.

480.	Kanaya AM, Kandula NR, Ewing SK, Herrington D, Liu K, Blaha MJ, et al. Comparing coronary artery calcium among U.S. South Asians with four racial/ethnic groups: the MASALA and MESA studies. Atherosclerosis. 2014;234(1):102-7. Epub 20140228. doi: 10.1016/j.atherosclerosis.2014.02.017. PubMed PMID: 24632509; PubMed Central PMCID: PMCPMC4005416.

481.	Ware EB, Mukherjee B, Sun YV, Diez-Roux AV, Kardia SL, Smith JA. Comparative genome-wide association studies of a depressive symptom phenotype in a repeated measures setting by race/ethnicity in the Multi-Ethnic Study of Atherosclerosis. BMC Genet. 2015;16:118. Epub 20151012. doi: 10.1186/s12863-015-0274-0. PubMed PMID: 26459564; PubMed Central PMCID: PMCPMC4603946.

482.	Gujral UP, Vittinghoff E, Mongraw-Chaffin M, Vaidya D, Kandula NR, Allison M, et al. Cardiometabolic Abnormalities Among Normal-Weight Persons From Five Racial/Ethnic Groups in the United States: A Cross-sectional Analysis of Two Cohort Studies. Ann Intern Med. 2017;166(9):628-36. Epub 20170404. doi: 10.7326/m16-1895. PubMed PMID: 28384781; PubMed Central PMCID: PMCPMC5545925.

483.	Yadlowsky S, Hayward RA, Sussman JB, McClelland RL, Min YI, Basu S. Clinical Implications of Revised Pooled Cohort Equations for Estimating Atherosclerotic Cardiovascular Disease Risk. Ann Intern Med. 2018;169(1):20-9. Epub 2018/06/06. doi: 10.7326/M17-3011. PubMed PMID: 29868850.

484.	Garg SK, Lin F, Kandula N, Ding J, Carr J, Allison M, et al. Ectopic Fat Depots and Coronary Artery Calcium in South Asians Compared With Other Racial/Ethnic Groups. J Am Heart Assoc. 2016;5(11). Epub 20161117. doi: 10.1161/jaha.116.004257. PubMed PMID: 27856485; PubMed Central PMCID: PMCPMC5210365.

485.	Hu J, Yao J, Deng S, Balasubramanian R, Jiménez MC, Li J, et al. Differences in Metabolomic Profiles Between Black and White Women and Risk of Coronary Heart Disease: an Observational Study of Women From Four US Cohorts. Circ Res. 2022;131(7):601-15. Epub 20220902. doi: 10.1161/circresaha.121.320134. PubMed PMID: 36052690; PubMed Central PMCID: PMCPMC9473718.

486.	Breeze CE, Batorsky A, Lee MK, Szeto MD, Xu X, McCartney DL, et al. Epigenome-wide association study of kidney function identifies trans-ethnic and ethnic-specific loci. Genome Med. 2021;13(1):74. Epub 2021/05/02. doi: 10.1186/s13073-021-00877-z. PubMed PMID: 33931109; PubMed Central PMCID: PMCPMC8088054.

487.	Rodriguez LA, Jin Y, Talegawkar SA, Otto MCO, Kandula NR, Herrington DM, et al. Differences in Diet Quality among Multiple US Racial/Ethnic Groups from the Mediators of Atherosclerosis in South Asians Living in America (MASALA) Study and the Multi-Ethnic Study of Atherosclerosis (MESA). J Nutr. 2020;150(6):1509-15. doi: 10.1093/jn/nxaa050. PubMed PMID: 32133497; PubMed Central PMCID: PMCPMC7269747.

488.	Segar MW, Jaeger BC, Patel KV, Nambi V, Ndumele CE, Correa A, et al. Development and Validation of Machine Learning-Based Race-Specific Models to Predict 10-Year Risk of Heart Failure: A Multicohort Analysis. Circulation. 2021;143(24):2370-83. Epub 20210413. doi: 10.1161/circulationaha.120.053134. PubMed PMID: 33845593.

489.	Al Rifai M, Kanaya AM, Kandula NR, Cainzos-Achirica M, Patel J, Budoff M, et al. Distribution of calcium volume, density, number, and type of coronary vessel with calcified plaque in South Asians in the US and other race/ethnic groups: The MASALA and MESA studies. Atherosclerosis. 2021;317:16-21. Epub 20201207. doi: 10.1016/j.atherosclerosis.2020.12.003. PubMed PMID: 33333344; PubMed Central PMCID: PMCPMC7790973.

490.	Al Rifai M, Cainzos-Achirica M, Kanaya AM, Kandula NR, Dardardi Z, Joshi PH, et al. Discordance between 10-year cardiovascular risk estimates using the ACC/AHA 2013 estimator and coronary artery calcium in individuals from 5 racial/ethnic groups: Comparing MASALA and MESA. Atherosclerosis. 2018;279:122-9. Epub 20180915. doi: 10.1016/j.atherosclerosis.2018.09.015. PubMed PMID: 30262414; PubMed Central PMCID: PMCPMC6295226.

491.	US Surgeon General. Our Epidemic of Loneliness and Isolation. 2023.

492.	Li J, Vitiello MV, Gooneratne NS. Sleep in Normal Aging. Sleep Med Clin. 2018;13(1):1-11. Epub 2018/02/08. doi: 10.1016/j.jsmc.2017.09.001. PubMed PMID: 29412976; PubMed Central PMCID: PMCPMC5841578.

493.	Misiura MB, Butts B, Hammerschlag B, Munkombwe C, Bird A, Fyffe M, et al. Intersectionality in Alzheimer's Disease: The Role of Female Sex and Black American Race in the Development and Prevalence of Alzheimer's Disease. Neurotherapeutics. 2023;20(4):1019-36. Epub 2023/07/25. doi: 10.1007/s13311-023-01408-x. PubMed PMID: 37490246; PubMed Central PMCID: PMCPMC10457280.

494.	Baiden D, Parry M, Nerenberg K, Hillan EM, Dogba MJ. Connecting the Dots: Structural Racism, Intersectionality, and Cardiovascular Health Outcomes for African, Caribbean, and Black Mothers. Health Equity. 2022;6(1):402-5. Epub 2022/07/09. doi: 10.1089/heq.2021.0077. PubMed PMID: 35801154; PubMed Central PMCID: PMCPMC9257546.

495.	American Association of Retired Persons. AARG Livability Index: Great neighborhoods for all ages [5/4/2024]. Available from: https://livabilityindex.aarp.org.
