# Supplementary material for: Structural and social determinants of health: The multi-ethnic study of atherosclerosis
Source: PLoS One. 2024 Nov 18;19(11):e0313625. doi: 10.1371/journal.pone.0313625 (PMC11573213; doi:10.1371/journal.pone.0313625)
Supplement: Attachment — Submitted filename: ResponsetoReviewers_Oct22.docx [file pone.0313625.s017.docx]

**Response to Reviewers**

**Reviewer #1:**

1.The manuscript is technically sound, and the data do support the conclusions.
2. The statistical analysis has been performed appropriately and rigorously.
3. The authors have made all data underlying the findings in their manuscript fully available.
4. The manuscript is presented in an intelligible fashion and written in standard English.

**Response**: Thank you for your review and comments.

**Reviewer #2:**

This is a well written paper. It has addressed the objectives well. The review used a big data with many variables, which is the strength of the study. However is there any theoritical framework that support the domains used.

**Response**: Thank you for your review and comment. Yes, we used the Schulz framework for Social Determinants of Health and Environmental Health Promotion, which we made more explicit in lines 95-102: “Our literature review is informed by the Schulz framework of Social Determinants of Health and Environmental Health Promotion, and thus we categorized the SSDOH according the categories outlined in the framework as follows: 1) macrosocial/structural factors (e.g., residential segregation and ideologies such as racism); 2) individual-level inequalities (e.g., employment, income, health insurance, and education); 3) built environment (e.g., land use, transportation access, and park space); 4) social context (e.g., neighborhood social cohesion and neighborhood socioeconomic status); 5) stressors (e.g., air pollution, chronic stress, violence and crime); 6) social integration and social support (e.g., social participation and social support) [13]”.

**Reviewer #4:**

This paper includes all categories of SDOH as defined by the CDC, except for "health care and quality." This would be an important SDOH to look at as it is intertwined with the "inequalities" assessed in the paper.

**Response**: We originally included papers that examine health insurance coverage under the “Inequalities” category. The results for those studies are reported on lines 275-276: “Consistently, access to health insurance was associated with better health outcomes [82, 195, 278, 284].”

For clarity, we have added health insurance as an example of the inequalities category listed in the methods section: lines 95-102: “Our literature review is informed by the Schulz framework of Social Determinants of Health and Environmental Health Promotion, and thus we categorized the SSDOH according the categories outlined in the framework as follows: 1) macrosocial/structural factors (e.g., residential segregation and ideologies such as racism); 2) individual-level inequalities (e.g., employment, income, health insurance, and education); 3) built environment (e.g., land use, transportation access, and park space); 4) social context (e.g., neighborhood social cohesion and neighborhood socioeconomic status); 5) stressors (e.g., air pollution, chronic stress, violence and crime); 6) social integration and social support (e.g., social participation and social support) [13]”.

None of the studies included data on health care access, which we have now added to the results section on Built Environment (lines 319-320) as follows: “No papers focused on health care access (e.g., density of health care facilities in the neighborhood).”

I like that the paper uses very simple methods to identify gaps in research that can be utilized and further studied through MESA.

**Response**: Thank you.

I would be curious to see how the variables in the 47 studies that combine MESA with other cohort studies differ in SDOH variables compared to the MESA-only studies (sub-analysis).

**Response**: We have added the following text to the results section (lines 517-524): “Among these 47 papers, 85% included macrosocial measures, 15% included inequality measures, 0% included built environment measures, 11% included social context measures, 15% included stressor measures, and 6% included social support measures (percentages not mutually exclusive since some papers include more than one SSDOH). In comparison to the full sample of papers, those combining MESA data with other cohorts more often included macrosocial measures (85% vs 57%), fewer (none) included built environment measures (0% versus 14%), and fewer included stressor measures (15% versus 34%) (Table 2).”
